# Absent from DNA and protein: genomic characterization of nullomers and nullpeptides across functional categories and evolution

**DOI:** 10.1186/s13059-021-02459-z

**Published:** 2021-08-25

**Authors:** Ilias Georgakopoulos-Soares, Ofer Yizhar-Barnea, Ioannis Mouratidis, Martin Hemberg, Nadav Ahituv

**Affiliations:** 1 Department of Bioengineering and Therapeutic Sciences, University of California San Francisco, San Francisco, CA USA; 2 Institute for Human Genetics, University of California San Francisco, San Francisco, CA USA; 3 Department of Computer Science, Katholieke Universiteit Leuven, Leuven, Belgium; 4 Evergrande Center for Immunologic Diseases, Harvard Medical School and Brigham and Women’s Hospital, Boston, MA USA; 5 Wellcome Sanger Institute, Wellcome Genome Campus, Hinxton, UK

**Keywords:** Nullomers, Nullpeptides, Primes, Negative selection, Transposable elements, Human population, Phylogenetics

## Abstract

**Abstract:**

Nullomers and nullpeptides are short DNA or amino acid sequences that are absent from a genome or proteome, respectively. One potential cause for their absence could be their having a detrimental impact on an organism.

**Results:**

Here, we identify all possible nullomers and nullpeptides in the genomes and proteomes of thirty eukaryotes and demonstrate that a significant proportion of these sequences are under negative selection. We also identify nullomers that are unique to specific functional categories: coding sequences, exons, introns, 5′UTR, 3′UTR, promoters, and show that coding sequence and promoter nullomers are most likely to be selected against. By analyzing all protein sequences across the tree of life, we further identify 36,081 peptides up to six amino acids in length that do not exist in any known organism, termed primes. We next characterize all possible single base pair mutations that can lead to the appearance of a nullomer in the human genome, observing a significantly higher number of mutations than expected by chance for specific nullomer sequences in transposable elements, likely due to their suppression. We also annotate nullomers that appear due to naturally occurring variants and show that a subset of them can be used to distinguish between different human populations. Analysis of nullomers and nullpeptides across vertebrate evolution shows they can also be used as phylogenetic classifiers.

**Conclusions:**

We provide a catalog of nullomers and nullpeptides in distinct functional categories, develop methods to systematically study them, and highlight the use of variability in these sequences in other analyses

**Supplementary Information:**

The online version contains supplementary material available at 10.1186/s13059-021-02459-z.

## Background

Nullomers are short DNA sequences, defined in this manuscript as being up to 15 base pairs (bp) in length, that do not exist within a certain genome [1]. While the absence of these sequences could be coincidental, studies of mammalian genomes have shown that a larger number of > 10 bp genetic sequences are identified as being nullomers than what would be expected by chance [1–3]. One hypothesis that was suggested for their genomic absence was having multiple CpGs which could lead to higher mutation rates [4]. Previous work looking at dinucleotide content has excluded this possibility and suggested that natural selection is a more probable explanation for their absence [2]. This could be due to deleterious properties of a peptide, or if the sequence is noncoding, through an effect on gene regulation, DNA shape, DNA stability, or other unknown causes. To date, only three human nullomers have been functionally characterized, two of which were shown to lead to lethality in several cancerous cell types when delivered as exogenic synthetic peptides [5, 6]. It was also shown that some nucleotide or amino acid sequences are still missing when examining closely related species, for example gorilla, chimp, and mouse [1, 2]. A more extreme case of evolutionary exclusion are nullomer primes, kmers that are found absent from all examined species [1]. Previous work utilized the genome of twelve species (human, chimp, and ten other non-primates) to identify 60,370 nullomers absent across them for 15 bp length. Recent work that compared minimal absent words (MAW) between human, bacteria, and viruses showed that human MAWs are frequently present in bacteria, suggesting a role in immune function [3]. In viruses, these sequences were generally missing likely due to host mimicry.

To find nullomers that could be deleterious due to protein coding function, a complementary approach could be used; identifying amino acid sequences that are missing from the proteome, termed nullpeptides. Here, we define nullpeptides as sequences that are up to seven amino acids (aa) in length that are absent from the proteome and nullpeptide primes as peptides that are absent from all known protein sequences. Previous work, carried out in 2009, identified 417 five amino acid primes that are not present in the universal proteome (over 6 million proteins analyzed at that time )[7]. Recent work characterizing MAWs from the proteome found that a single substitution in them is predicted to be harmful for the protein [3]. Functional characterization of a 5mer peptide (KWCEC), that is extremely rare within the universal proteome and absent from the human proteome, showed that it could potentially enhance immunogenicity when administered alongside an antigen [8].

With the plethora of available genomes, we set out to comprehensively characterize nullomers and nullpeptides in thirty different species (Additional file 2: Table S1). In addition, taking advantage of the annotations available for these genomes, we characterized nullomers in specific functional categories: coding sequence, exons, introns, 5′UTR, 3′UTR, and promoters as well as in enhancer regions, CTCF sites, and open chromatin regions. Using various ranking metrics, we showed that nullomers and nullpeptides are under negative selection. By analyzing the large resources available for human variation, such as the Genome Aggregation Database (gnomAD) [9], we characterized how these variants can lead to the materialization of nullomers, termed variant-associated nullomers. Finally, we showed how these sequences could be used as phylogenetic classifiers.

## Results

### Nullomer annotation

**Fig. 1:**
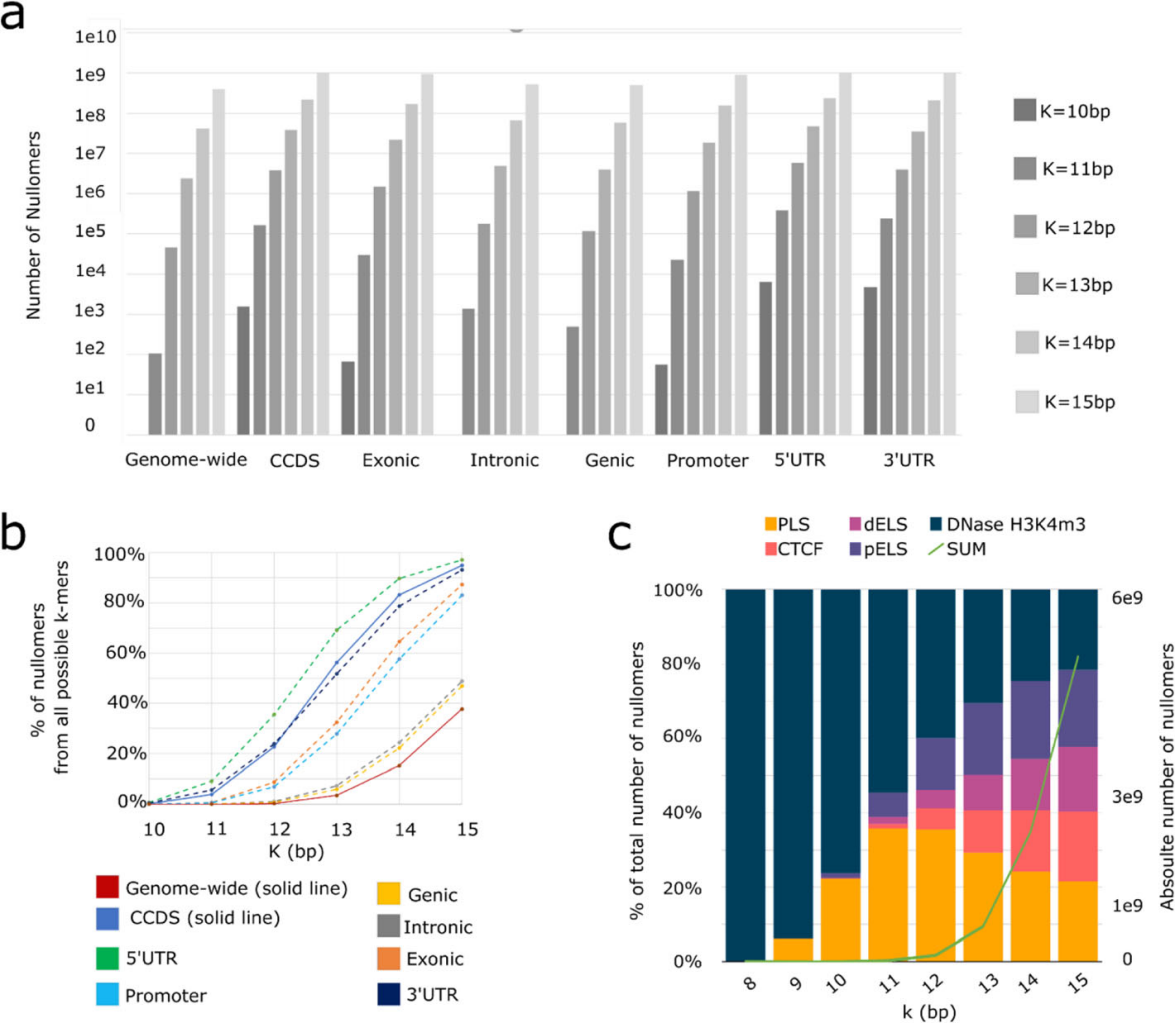
Distribution of nullomers in the human genome and its functional subcompartments. **a** The number of nullomers in the human genome and its sub-categories as a function of nullomer length. **b** Proportion of kmer space being nullomers for nullomers *K* = 10–15 bp in the human genome and its functional subcompartments. **c** Proportion of nullomer space across reference *cis*-regulatory regions for nullomers *K* = 10–15 bp. PLS corresponds to promoter-like signature, pELS to proximal enhancer-like signature, dELS to distal enhancer-like signature, DNase H3K4me3 to DNase and H3K4me3 sites, and CTCF to CTCF-only and CTCF-bound sites

We first set out to generate a comprehensive list of all human DNA nullomers for each kmer length up to 15 bp (Fig. 1a) (see “Methods”). The shortest nullomer length studied was the minimal length at which nullomers appeared. The upper nullomer length was selected as the largest kmer length for which the number of kmers found in the genome is higher than the number of nullomers. For larger lengths, the majority of possible kmers do not appear in the genome, making it harder to characterize the subset of nullomers that are biologically relevant. The shortest nullomers we found in the human genome were 11 bp long (Table 1 and Fig. 1a), with a total of 104 nullomers at this length, consistent with a previous report [2]. The number of nullomers grows rapidly with increasing kmer length (Fig. 1a, Table 1). For example, we find ~ 40 million nullomers at *K* = 14 and ~ 400 million at *K* = 15 in the human genome. Moreover, for *K* = 12 the nullomer space represents only 0.26% of all possible 12mers, whereas for *K* = 15, the nullomer space represents 37.8% of all possible 15mers (Fig. 1b).

**Table 1.** Number of nullomers found in the human genome and in various functional categories for kmer length up to 15 bp

Length	Genome	Genic	CCDS	Exonic	Intronic	Promoter	5′UTR	3′UTR
Size of sequence space (bp)	30493128927 (100%)	1500831648 (46.77%)	34823267 1.09%	97533778 3.04%	1432130176 44.62%	151349111 4.72%	19990533 0.62%	49256976 1.53%
10	0	0	1553	65	0	55	6321	4783
11	104	480	159,400	29,908	1342	22,390	38,0030	235,512
12	44,287	115,211	3,798,220	1,459,145	179,991	1,157,400	5,954,005	4,008,652
13	2,347,664	3,986,232	37,728,254	21,795,714	4,889,886	18,632,872	46,458,594	34,749,772
14	40,798,250	59,680,430	222,865,418	173,484,802	65,434,475	155,022,567	240,489,521	211,238,387
15	405,373,474	504,426,674	1,018,873,404	935,392,848	523,843,630	889,865,066	1,042,269,144	999,624,368

We next characterized nullomers in different functional categories. These include genic regions [mRNA sequence from transcription start site (TSS) to transcription end site (TES)], consensus coding sequences (CCDS), exons (both coding and noncoding), introns, 5′UTR, 3′UTR, and promoters (defined as − 2500 to + 500 around the TSS) (see “Methods”). Because these regions are subsets of the whole genome, the number of nullomers identified is larger. Analogous to nullomers within the entire human genome, we did not find genic or intronic nullomers for *K* < 11. However, we identified 1553 CCDS, 65 exonic, 6321 5′UTR, 4783 3′UTR, and 55 promoter nullomers at a length of *K* = 10. Similar to our nullomer analyses for the entire human genome, we also observed an exponential increase in the number of identified nullomers with increasing kmer length for the various functional categories (Fig. 1a, Table 1). As expected, when increasing the kmer length, nullomers represented a larger portion of the kmer space (Fig. 1b).

We further characterized nullomers in the noncoding regulatory sequence space by searching ENCODE’s candidate Cis-Regulatory Elements (cCREs) which were sub-divided in the following categories: promoter-like, proximal and distal enhancer-like elements, CTCF sites, and DNase-H3K4me3 sites which are indicative of promoters (Fig. 1c, Additional file 1: Fig. S1c-d) [10, 11]. This repository of human DNA nullomers serves as the basis for all subsequent downstream analyses and comparisons in this article.

### Nullomers are under negative selection

**Fig. 2:**
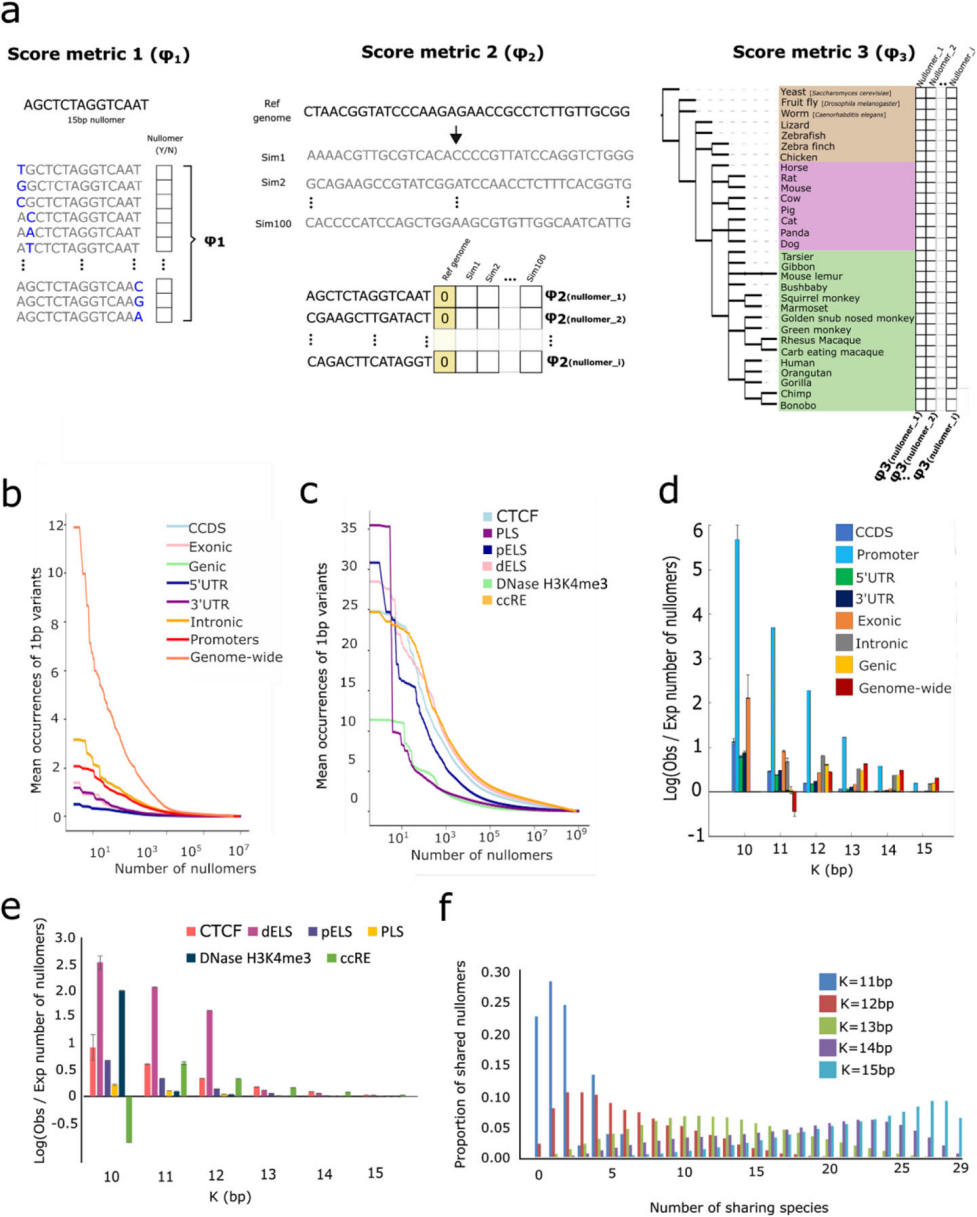
Nullomers are observed more frequently than expected by chance in the human genome and various functional components. **a** Schematic representation of the three nullomer scoring metrics. **b** *φ*1 metric score for each nullomer across the genome and the genomic subcompartments for *K* = 15. **c** *φ*1 metric score for each nullomer in the ENCODE cCRE categories for *K* = 15. **d** *φ*2 metric score, log transformed number of occurrences of *N* nullomers in the genome and various functional categories relative to their occurrences in simulations across *K* = 10–15 bp. **e** *φ*2 metric score log transformed number of occurrences of nullomers in the ENCODE cCRE categories relative to their occurrences in simulations as a function of nullomer length for *K* = 10–15 bp. For **d, e**, simulations were performed controlling for trinucleotide content. Error bars represent standard deviation of the number of nullomers obtained from *n* = 100 simulations of each genomic annotation category and has been log transformed. **f** *φ*3 metric score showing the proportion of human nullomers found across 29 other species as a function of nullomer length for *K* = 11–15 bp. For panels **c** and **e**, PLS corresponds to promoter-like signature, pELS to proximal enhancer-like signature, dELS to distal enhancer-like signature, DNase H3K4me3 to DNase and H3K4me3 sites, and CTCF to CTCF-only and CTCF-bound sites

We next set out to test if these sequences are absent due to mere chance or if they are under negative selection. Lacking a conventional statistical measure to determine the significance of a nullomer, we created our own scoring matrix, *φ*N. The *φ*N score encompasses three tiers of ranking (Fig. 2a), described in detail in the “Methods” section. All three tiers estimate deviations from the number of the expected occurrences for each kmer motif (not necessarily a nullomer) in order to identify kmers that occur more or less frequently than expected by chance. Our first score metric (*φ*1), is the mean number of occurrences of all the 1 bp possible substitution kmers for each nullomer sequence across the search space. The second scoring metric (*φ*2), is based on simulations of the human genome, its genomic subcompartments controlling for mono-, di-, or trinucleotide content. Our third score metric (*φ*3) is evolutionary driven, based on nullomers in the genome of 30 species (including humans) across vast evolutionary distances.

With our first metric, *φ*1, we found that for a subset of nullomers, single base pair substitution variants were very frequent (Fig. 2b, c), enabling us to prioritize nullomers controlling for nucleotide composition. As a second metric, we performed simulations controlling for mono- (*n* = 100) / di- (*n* = 100) / trinucleotide (*n* = 100) content of each regulatory component to estimate the number of occurrences of nullomers by chance in the human genome and its subcompartments. Ιn every simulation, each sequence was shuffled controlling for mono- / di- / trinucleotide content. We determined the ratio between the number of observed versus the number of simulated (expected) nullomers, finding a higher number of nullomers than expected by chance (Fig. 2d, e). Simulation scores (*φ*2) showed a higher number of nullomers in the human genome for every nullomer length between *K* = 12 and 15, than expected by chance (Fig. 2d), which contradict previous results obtained with a Poisson model [2]. For *K* = 11, we find more sequences in the simulations than in the genome, likely due to the small number of nullomers (*N* = 104). We observed the most pronounced number of nullomers compared to the simulations for promoters, genic regions, and introns (Fig. 2d). We also performed the same analysis separating the different nullomer lengths, finding larger differences between the expected and the observed number of nullomers for shorter nullomers in the genomic subcompartments (Fig. 2e). This is likely the result of a larger kmer space for nullomers with increasing kmer length (Fig. 1c). Leveraging *φ*3, the average occurrences in 30 species (Fig. 2f), we identified a total of 124 genome nullomers absent across the species of 13 bp length, consisting of only 0.00022% of the human genome nullomer space of the same length. Combined, our scoring metrics (*φN*) show that nullomers are under selective pressure.

### Higher-order nullomers as a ranking criterion

An alternate approach to prioritize nullomers that are more likely to have a functional consequence is the characterization of higher-order nullomers, as shown previously [2]. These are sequences where more than one nucleotide change is needed for them to cease being a nullomer. The hypothesis is that if a sequence is under strong negative selection, similar sequences that only differ by a one base pair substitution will also be absent from the genome. This approach could also be used to rank nullomers, thereby highlighting nullomers under strong negative selection. We mapped the distribution of first-order nullomers in the human genome and its functional subcompartments, where more than one nucleotide substitution is required to disrupt a nullomer (Table 2). Examination of nullomers in the entire genome reveals 7874 nullomers of 14 bp to be the shortest first-order nullomers found, and their number increases by three orders of magnitude at 15 bp with over 2.5 million first-order nullomers (Table 2). Focusing on specific functional categories, we find the most first-order nullomers were identified in 5′UTRs (Table 2), possibly due to selection against stop codons and upstream open reading frames (uORFs) that can have a large effect on protein translation [12].

**Table 2.** Number of first-order nullomers found at each genomic element for kmer length up to 15 bp

Length	Genome	Genic	CCDS	Exonic	Intronic	Promoter	5′UTR	3′UTR
10	0	0	0	0	0	0	0	0
11	0	0	0	0	0	0	0	0
12	0	0	210	4	0	0	1989	0
13	0	10	167,362	9400	56	5172	682,524	378,186
14	7874	39,268	13,963,269	2,542,806	93,417	1,727,683	31,484,149	16,310,842
15	2,502,376	6,141,882	292,832,416	104,630,926	9,288,682	77,629,000	447,490,130	267,624,098

### Genome-wide maps of resurfacing nullomers

**Fig. 3:**
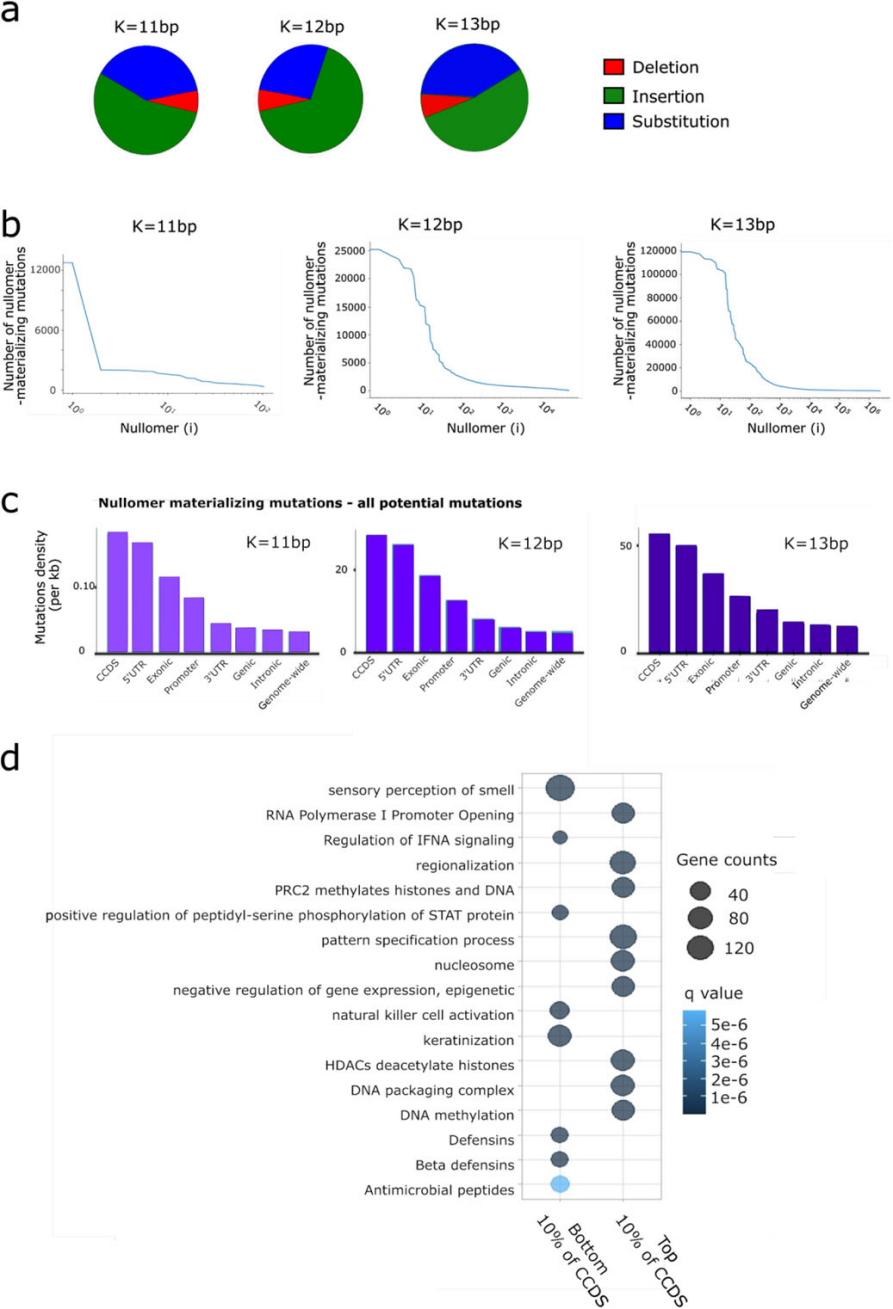
Variant-associated nullomers in the human genome. **a** Pie charts representing the proportion of nullomer materializing mutations in the genome that are substitutions and single base pair insertions and deletions for *K* = 11–13 bp. **b** Number of mutations that materialize each nullomer for *K* = 11-13 bp nullomer lengths across the human genome. **c** Density of mutations that can materialize nullomers across genomic subcompartments for *K* = 11–13 bp nullomer lengths. **d** Gene Ontology (GO) analysis of the top 10% nullomer-resurfacing (*K* = 11 bp) mutation dense CCDS and the bottom 10% (least dense CCDS). Circle diameter correlates to the number of contributing genes and the circle shade correlates to GO term *q*-value

Nullomers that are absent from the human genome could resurface due to genetic variation. To test this, we generated genome-wide maps that annotate where nullomers could materialize due to nucleotide variation, including both nucleotide substitutions and one base pair insertions and deletions. We found 100,587 potential variants that materialize nullomers of 11 bp length, 15,822,585 potential variants of 12 bp length, and 413,803,913 potential variants of 13 bp length. The most frequent mutation type was insertions followed by substitutions (Fig. 3a). Next, we corrected for the number of possible kmer substitutions, insertions, and deletions that can generate a nullomer to investigate preferences dependent on the mutation category. For 13 bp nullomers, there are 13 possible deletions (13% of mutations), 39 possible substitutions (39% of possible mutations), and 48 possible insertions (48% of mutations). When comparing the expected to the observed number of mutations for each mutation type, we found enrichments of 0.56-fold, 1.04-fold, and 1.09-fold respectively. This indicated a relative depletion of nullomer-generating deletions in the human genome. Substitutions were further analyzed and we found that A->C, T->C and G->C are the most frequent substitution types (Additional file 1: Fig. S2a-c). These substitution types are in accordance with the mutation model presented in Sved and Bird [13] and could suggest that reverse mutations occurred during evolution, in turn resulting in nullomer formation. This could partially explain the mechanism driving nullomer sequence content. Considering trinucleotide context, we found that CTG and CAG are the most common, both before and after correction for trinucleotide frequency across the genome (Additional file 1: Fig. S2d-f).

We examined the number of possible mutations that can create each nullomer, finding substantial differences with more than 120,000 mutations generating a small subset of nullomers (Fig. 3b). For *K* = 11, 12, and 13 the nullomers that can resurface in most places in the human genome were “CCGACGATCGT,” “ACCGTCGTTAGC,” and “TCGTTGCGAAACG” with 12,948, 25,339, and 120,838 occurrences respectively. Interestingly, 95.6% of mutations resurfacing “CCGACGATCGT” overlapped SINE elements and specifically Alu repeats (95.5%). The vast majority of the mutations (93.9%) that could create this nullomer is through the insertion of a C between “CCGA” and “GATCGT.” Similarly, mutations creating “ACCGTCGTTAGC” and “TCGTTGCGAAACG” nullomers were also insertions, most often observed in Alu repeats (98.0% and 95.6% respectively). The absence of these sequences given the number of possible mutations that can generate them suggests selection against the re-activation of endogenous retroelements or activity of cellular mechanisms that deactivated them during evolution.

Throughout the genome, we observed that nullomer materializing mutations were more frequent in genic regions compared to intergenic (between genes) (Fig. 3c). We also investigated if the putative mutations were more likely to overlap the aforementioned functional categories (genic, CCDS, exons, introns, 5′UTR, 3′UTR, promoters). We found that CCDS, exonic, 5′UTR, and promoter regions had the highest density of variant-associated nullomers, followed by 3′UTR, genic, and intronic (Fig. 3c). The observation that CCDS regions have a higher density of potential nullomer materializing variants further suggests that these sequences are under strong selective constraints. We performed the same analysis using the ENCODE cCRE functional partitioning of the regulatory genome and found that promoters exhibited the highest density of potential nullomer materializing mutations among regulatory regions (Additional file 1: Fig. S3a).

We wanted to assess whether specific functional categories are associated with genes that have higher and lower putative nullomer-resurfacing mutations. We measured the density of putative nullomer-resurfacing mutations in CCDS regions for each gene and performed gene ontology (GO) term analysis (see “Methods”). We found that genes with the highest density of nullomer materializing mutations were associated with epigenetic regulatory processes and DNA molecule organization, whereas genes with the lowest density of nullomer materializing mutations were associated with processes such as cell to cell contact, detection of chemical stimuli, receptors, and axonogenesis among others (Fig. 3d, Additional file 1: Fig. S4).

### Characterization of naturally occurring variants that cause nullomers to resurface

**Fig. 4:**
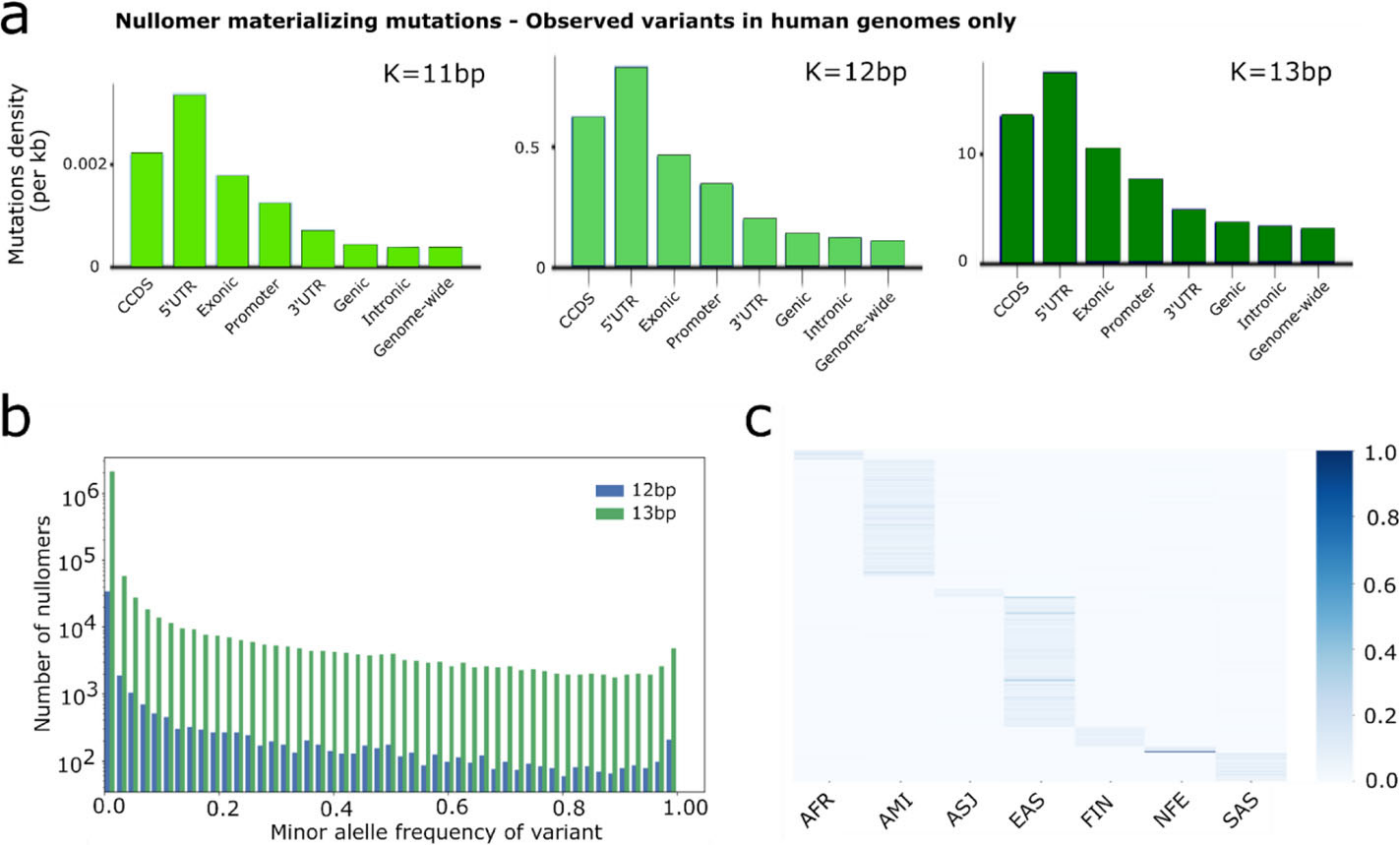
Characterization of variant materializing nullomers relative to minor allele frequencies and population stratifications. **a** Density of substitution variants that materialize nullomers for *K* = 11–13 bp across genomic subcompartments. **b** Number of human nullomers materializing from substitution variants represented as a histogram for each 2% increments in variant minor allele frequencies. **c** Minor allele frequency of population-specific nullomer-generating substitution variants across eight populations

We next set out to test whether nullomers could materialize due to naturally occurring substitution variants in the human population, termed here as variant-associated nullomers. To investigate this, we took advantage of a collection of over 707 million variants annotated in gnomAD [9]. For *K* = 11, we found 107 substitution variants that result in 67 nullomers no longer being absent from the human genome. We mapped these variants and their density in the whole genome and in each functional unit (Fig. 4a). We found that the two categories with the highest density of variants that materialize nullomers were 5′UTR and CCDS regions (Fig. 4a), similar to the findings we observed for the genome-wide maps of potential mutation sites (Fig. 3c). We performed the same process using the ENCODE cCRE functional partitioning of the regulatory genome and found that promoters exhibited the highest density of variants that materialize nullomers among regulatory regions (Fig. S3b).

We analyzed the allele frequency of these variants and found that many variants are rare (< 1%). For *K* = 11, we estimate that 22 of the nullomers (21% of all nullomers) have a probability > 5% of being present due to polymorphisms and 33 (32% of all nullomers) are present with a probability < 1% (Fig. 4b). For *K* = 12, there are 319,347 variants that result in 43,610 nullomers (98% of all nullomers) no longer being absent. Analysis of the allele frequencies suggests that 8315 nullomers (19% of all nullomers) are likely to be found in > 5% of the population and 31,809 (72% of all nullomers) in < 1%. For *K* = 13, many variants will lead to the creation of more than one nullomer, and we find a total of 5,591,595 variants that result in 2,131,502 nullomers resurfacing (91% of all nullomers).

We estimated the proportion of nullomers that resurface in the human population through common variants (aggregate probability of resurfacing > 0.05). We find that for 12mers 35,972 nullomers do not appear through population variants representing 81% of the nullomers for this length. Similarly, for 13mers, we find that 2,126,810 of the nullomers do not appear, representing 91% of nullomers for that length. We also estimate the likelihood of nullomers resurfacing with a probability threshold of > 0.01 and find that 70% of 12mer nullomers do not resurface, while for 13mer nullomers 84% do not resurface. Therefore, we conclude that the majority of nullomers for lengths *K* = 11 to *K* = 13 do not appear from common variants, which likely reflects selection constraints against those sequences and which is in accordance with our earlier observations.

As some nullomers are most likely to materialize due to variants that are prevalent in specific populations, we tested whether certain nullomers are indicative of specific groups. Considering eight different populations (African, Amish, Admixed American, Ashkenazi Jews, East Asians, Finnish, Non-Finish European, South Asian) for *K* = 13, we found a total of 4273 nullomers that were common (> 5%) in one population and rare (< 1%) in all others (Fig. 4c).

In order to finalize our overall scoring metric for nullomers, we employed this population variability analysis and excluded nullomers that can emerge from human population variance. We combined all three scores mentioned thus far, and after excluding the common variant resulting nullomers, each nullomer(i) was assigned a summarized score, which was used as the basis for future experiments.

### Nullpeptide annotation

A complementary approach to identifying CCDS nullomers is to identify amino acid sequences that do not appear in the human proteome, defined as nullpeptides (Additional file 2: Tables S3-S4). We scanned the UniProt human reference proteome database [14] for nullpeptides of up to 7aa in length. The shortest nullpeptides we found were 4aa in length, totaling 207 across the human proteome. As expected, this number increased exponentially with length. For nullpeptides of lengths 5, 6, or 7aa, we identified 792,913, 55,524,544 and 1,269,204,068 nullpeptides, respectively (Additional file 2: Table S3).

### Nullpeptides are under negative selection

**Fig. 5:**
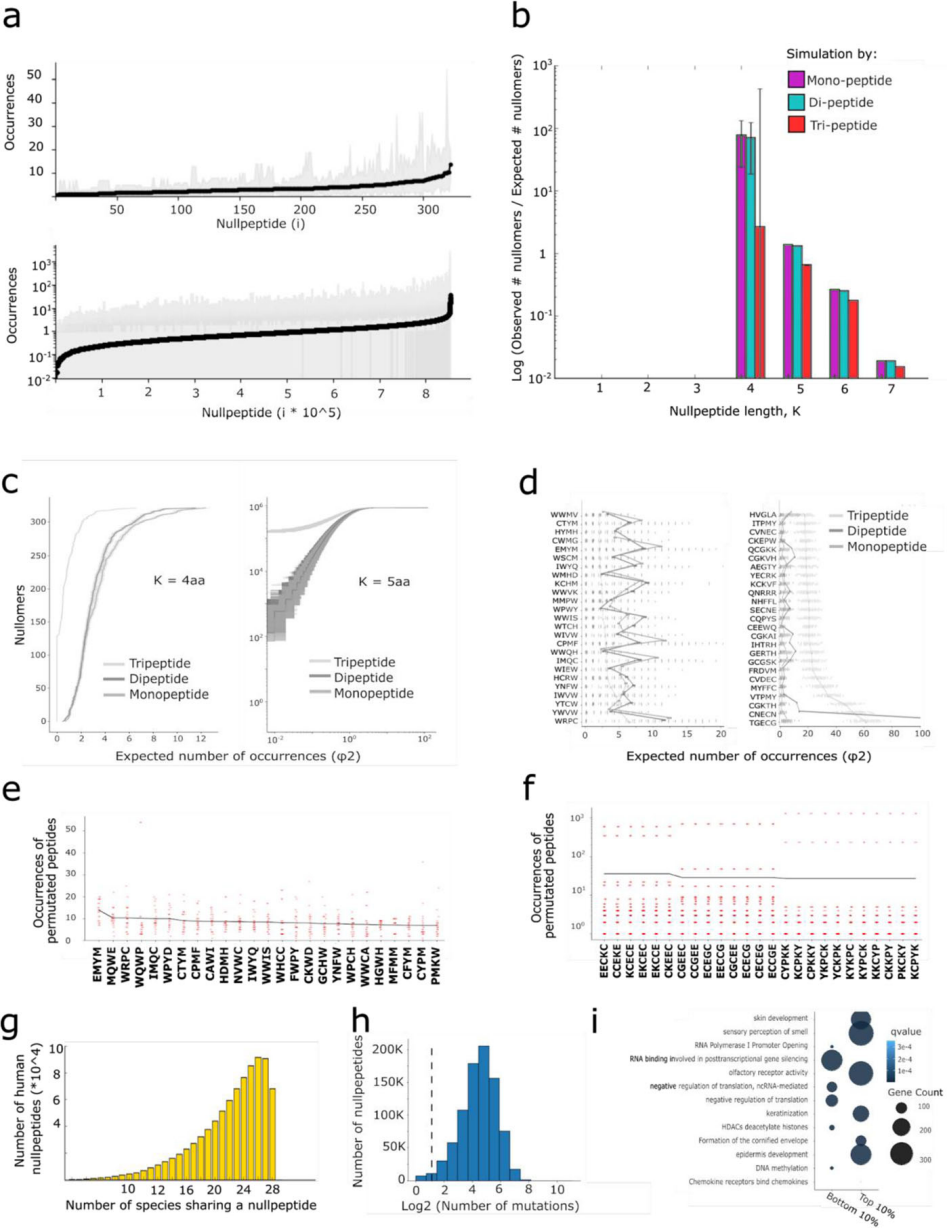
Human nullpeptide characterization. **a** Nullpeptide prioritization based on the number of occurrences of all permuted peptides of length 4aa (top) and 5aa (bottom). Black line indicates the mean number of occurrences of all permuted peptides with upper and lower lines indicating minimum and maximum number of occurrences across the permuted peptides. Nullpeptides were ordered based on the average number of times they were found in the simulations. **b** *φ*2 metric score number of occurrences of nullpeptides in the proteome relative to their occurrences in simulations controlled for mono-, di-, and tripeptide content of the proteome plotted as a function of nullomer length for *K* = 10–15 bp. Purple, turquoise, and red colors represent occurrences of nullpeptides in the simulations controlling mono-, di-, and tripeptide content, respectively. **c** Simulations showing that a large proportion of peptide nullomers (*K* = 4aa and *K* = 5aa) should be frequently observed in the human proteome and are thus likely under negative selection. **d** Depiction of the twenty-five most frequent nullomers in the simulations for *K* = 4 and *K* = 5 aa. **e, f** Number of occurrences of permuted peptides for each nullpeptide for *K* = 4 aa and *K* = 5aa. Black line represents the median occurrences across the permuted peptides. Only the twenty-five top ranked nullpeptides of 4- and 5-amino acid length are shown. **g** Number of species in which human nullpeptides of 4–5-amino acid length were identified. **h** Distribution of nullpeptides across the number of all possible substitutions that allow a nullpeptide to materialize. **i** Gene Ontology (GO) analysis of the top and bottom 10% genes based on the putative nullpeptide-resurfacing mutation density for *K* = 5aa nullpeptides. The circle diameter correlates to the number of contributing genes and the circle shade correlates to GO term *q*-value

Similar to nullomers, we next examined whether nullpeptides are under negative selection. Due to large differences in the frequency of amino acids in the proteome, a more suitable metric to prioritize nullpeptides would be to rank each of them by the mean number of occurrences of all their possible permutations, rather than all possible single amino acid changes. We reasoned that nullpeptides whose permuted amino acid sequences are on average more frequent would reflect the nullpeptides under selection pressures and that this method would at the same time correct for the imbalance in amino acid frequencies between kmer peptides. We found that for a subset of nullpeptides, their permuted peptides are frequently occurring in the human proteome. For 4aa nullpeptides, the average number of times the permuted peptides were found ranged between 0.666 and 13.818 occurrences, while for 5aa nullpeptides they ranged between 0 and 35.86 occurrences (Fig. 5a).

As a second measurement of selection against nullpeptides, we carried out simulations for each protein sequence of the reference proteome, controlling for monopeptide, dipeptide, and tripeptide content (100-fold simulations for each). We compared the expected number of nullpeptides across the simulations and found that in all cases we observed a larger number of nullpeptides in the reference proteome than in the simulations (Fig. 5b, Additional file 1: Fig. S5a-b), suggesting selection pressures at the protein level. The observed number of nullpeptides was significantly higher than expected based on the simulations, consistent with the results at the DNA level (Additional file 1: Fig. S5a-b). We defined the enrichment as the number of nullpeptides observed over the average number of nullomers identified across the proteome simulations. For nullpeptide length of 4aa, the enrichments were 1.96-fold, 1.84-fold, and 1.22-fold relative to simulations controlling for mono-, di-, and tripeptide content and for 5aa the enrichments were 1.19-fold, 1.14-fold, and 1.08-fold relative to mono-, di-, and tripeptide controls (Additional file 1: Fig. S5a-b). Comparing the frequency with which we observed each nullpeptide in the simulations, we found that the majority of nullpeptides appeared in the simulations, even when controlling for tripeptide content (Fig. 5c, d, Additional file 1: Fig.S5c-d). Importantly, we observed consistency between our two first metrics, with nullpeptides that scored as most likely under selection in one metric (*φ*1) also having a concordant score for our second metric (*φ*2) for *K* = 4 and 5aa (Additional file 1: Fig. S6). In particular, we sorted nullpeptides based on permutation and simulation scores. The nullpeptides in the top quantile, as sorted by the two separate scoring measures, were in all cases more likely to be shared between the two groups than expected by chance (*N* = 10,000, empirical *p* value < 0.0001). As an example, among the 25 top 4aa nullpeptides, multiple sequences were shared between the two metrics including EMYM, WRPC, IMQC, CTYM, CPMF, IWYQ, WWIS, and YNFW (Wilcoxon Signed-Rank, *p* value < 0.005 for top 25 nullpeptides for monopeptide, dipeptide, and tripeptide comparisons) (Fig. 5d,e). Similar results were obtained when performing a hypergeometric test for the top 10% of 5aa nullpeptides in permutations and simulations (Wilcoxon Signed-Rank, *p* value<e−100). When analyzing the amino acid composition of the annotated nullpeptides, we identified the amino acids Tryptophan (W), Methionine (M), and Cysteine (C) to appear consistently more in nullpeptides than in non-nullpeptide kmers of the same length (Additional file 1: Fig. S7a). This might be due to the importance of the M codon for translation initiation and that a single base pair change can cause W and C codons to become a stop codon, TAA [15, 16]. We also observed pronounced biases in dipeptide usage for nullpeptides relative to non-nullpeptides. For instance, we observed odds ratios of 11.67, 9.70, and 7.50, for WW, WM, and MW respectively (Additional file 1: Fig. S7b). Similarly to nullomers, the third metric we used was the number of species in which we identified each human nullpeptide in their proteome (Fig. 5g, Additional file 2: Table S2). As expected, a larger proportion of human nullpeptides of 5aa were also absent across multiple other species relative to 4aa nullpeptides, including multiple absent across all species studied (Additional file 1: Fig. S7c-d). Finally, we were also able to generate proteome-wide maps of all possible nucleotide substitutions that can materialize a nullpeptide and found substantial differences in the number of mutations that can generate each nullpeptide (Fig. 5h). Interestingly, we found that there are 3128 nullpeptides that cannot be created through single base pair substitutions. The most frequent nullpeptides that could be reached by a single substitution were QRIDT, WGKSF, and CKEWG with 671, 657, and 640 potential substitutions that could generate them. Combined, these results show that nullpeptides are likely under negative selection and can also be used to rank sequences for follow-up functional assays.

We measured the density of putative nullpeptide-resurfacing mutations in each protein and performed a GO term analysis. We found that genes with the highest density of potential nullpeptide materializing mutations were associated with sensory perception and skin-related terms, whereas genes with the lowest density of potential nullpeptide materializing mutations were associated with processes such as synapse organization, synaptic transmission, and synaptogenesis (Fig. 5i, Additional file 1: Fig. S8). A REACTOME pathway enrichment analysis augments the GO analysis by having a defined pathway oriented point of reference [17]. Pathway enrichment analysis identified that genes with the highest density of potential nullpeptide materializing mutations were associated with the olfactory signaling pathway, keratinization, and GPCR ligand binding, whereas those with the lowest density were associated with the neuronal system and transmission across chemical synapses (Additional file 1: Fig. S8).

We estimated the proportion of nullpeptides that resurface in the human population through common variants using the gnomAD cohort [9]. For an aggregate probability of resurfacing > 0.05, we find that for 4aa nullpeptides 307 do not appear through population variants representing 97% of the nullpeptides for this length. Similarly, for 5mers, we find that 603,910 of the nullpeptides do not appear, representing 98% of nullpeptides for that length. We also estimate the likelihood of nullpeptides resurfacing with a probability threshold of > 0.01 and find that 94% of 4aa nullpeptides do not resurface, while for 5aa nullpeptides 96% do not resurface.

### Nullomer annotation across evolution

**Fig. 6:**
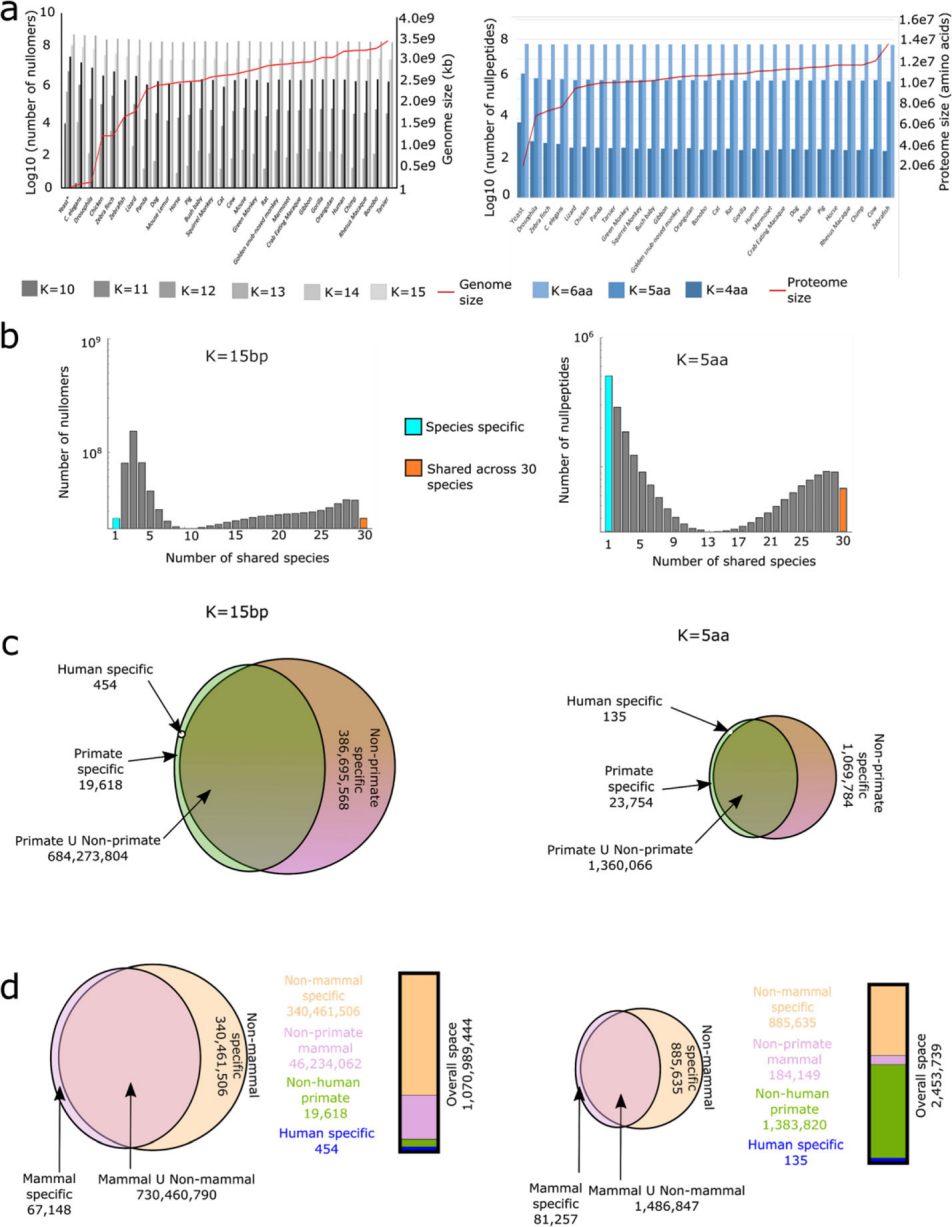
Characterization of nullomers across the genome of 30 species. **a** A bar plot representing the number of nullomers and nullpeptides found per species as a function of kmer length. The red line represents the genome and proteome size of each species in left and right panels, respectively. **b** Number of nullomers shared across multiple species for *K* = 15 bp and *K* = 5aa. **c** Intersection of nullomers (left) and nullpeptides (right) between humans, all other primates aside from human and non-primates species. **d** Intersection between mammalian and non-mammalian species for nullomers (left Venn diagram) and nullpeptides (right Venn diagram); adjacent to each is a staggered bar plot with cumulative number of nullomers/nullpeptides. The diameter of the circles and bar height are roughly correlated to differences in numbers

To identify nullomers that are persistently missing across evolution and in specific classes, order, or species, we repeated our analyses on 29 additional eukaryotic species. These include 14 primate genomes and 15 non-primate genomes (see Additional file 2: Table S1 for list of species). In addition, we also annotated nullomers in these genomes using the various functional categories (genic, CCDS, exonic, intronic, 5′UTR, 3′UTR, and promoters). As would be expected, we observed a negative correlation between genome size and number of nullomers (Fig. 6a, Pearson *r* = − 0.89). We also saw that the longer the nullomer, the larger the proportion was shared between species (Fig. 6b, Additional file 1: Fig. S9a-b). We next set out to identify nullomers that do not exist in any of the 30 organisms and ones that are unique to each species (Fig. 6b, Additional file 1: Fig. S9b). For *K* = 12, we did not find any shared nullomers between species. We found 124 nullomers absent from all 30 species at length 13 bp (0.00022% of all nullomers of similar length), 272,085 nullomers absent from all 30 species at length 14 bp (0.1014% of all nullomers of similar length), and 26,010,370 nullomers absent from all 30 species at length 15 bp (2.4% of all nullomers of similar length). Our comparison across species revealed 994 human-specific nullomers of *K* = 12 bp (0.0129% of all *K* = 12 bp nullomers) and 455 nullomers of *K* = 15 bp (0.000042% of all *K* = 15 bp nullomers) (Fig. 6b, c, Additional file 1: Fig. S9b-c, Additional file 2: Tables S3-S5).

Our analysis also allowed us to annotate, class, and order specific nullomers. For example, there are 240,321 and 684,293,876 nullomers found in at least one primate for 12 bp and 15 bp length respectively across their genomes (Fig. 6c, Additional file 1: Fig. S8b). For *K* = 15 bp, there are also 10,510 nullomers found only in all non-primate species. We also found 1 and 908,896 nullomers for 12 bp and 15 bp that were shared across all non-mammals but absent in all mammals (Fig. 6d, Additional file 1: Fig. S9c). For nullomers of *K* = 12 bp, we observed nullomers found only in non-mammals comprised the majority of the annotated nullomers (~ 96.2%), while within the mammalian nullomers, only 18% were not found in at least one primate species (Additional file 1: Fig. S9b-c). These results suggest that closely related species share a larger portion of their genome nullomers.

### Characterization of nullpeptides across evolution

We next characterized nullpeptides in 29 species in which we previously characterized nullomers, excluding mouse lemur for which a reference proteome was not available in UniProt (Additional file 2: Tables S3). We observed a negative correlation between proteome size and number of nullpeptides (Fig. 6a, Pearson *r* = − 0.97). We also saw that the longer the nullpeptide, the larger the proportion was shared between species (Fig. 6b, Additional file 1: Fig. S9a). For 4aa, we did not find any shared nullpeptides between species, similarly to what we had seen for nullomers of *K* = 12 bp. We annotated the number of nullpeptides that are only in humans but not in any other species studied, and found none for *K* = 4aa, 135 for *K* = 5aa, and 33 for *K* = 6aa (Additional file 2: Table S6). When expanding to primate-specific nullpeptides, i.e., missing from all primate proteomes, but could be found in at least one non-primate proteome, we found 16, 283,075 and 16,243,260 for lengths of 4aa, 5aa, and 6aa. Moreover, we found 0, 14, and 12 nullpeptides shared in all primates and absent across all non-primates for *K* of 4aa, 5aa, and 6aa respectively. We believe that the drop in either human- or primate-specific nullpeptides when comparing *K* = 5 and *K* = 6 is a result of the sharp increase in shared nullpeptides from only 9.4% of 4aa nullpeptides shared between primates and non-primates as opposed to 55.4% for *K* = 5aa and 95.3% of the *K* = 6aa (Fig. 6c, Additional file 1: Fig. S9b). For mammal-specific nullpeptides, we found 0, 133, and 314 nullpeptides that were shared across mammals and absent in all non-mammals for *K* = 4–6aa. We observed that primate nullpeptides increase from 74.5% of all mammalian nullpeptides at *K* = 4aa, to ~ 88% at *K* = 5aa, while taking a larger portion of the total nullpeptide space at the higher kmer length (56.4%, Fig. 6d, Additional file 1: Fig. S9c).

We also annotated amino acid sequences that are absent from all known species (not just the 29 analyzed proteomes) using the UniParc database (which had 1,030,456,800 proteins), termed nullpeptide primes. We found a total of 140,308,851 nullpeptide primes, with 36,081 and 140,272,770 for six and seven amino acids in length, respectively. No nullpeptide primes were observed for *K* < 6. To measure which amino acids are more common in nullpeptides, we calculated the frequency of each amino acid across prime and non-prime sequences from which we obtained enrichment patterns. We found that the amino acids W, M, C, similar to nullpeptides, along with Tyrosine (Y) and Histidine (H) are enriched in primes relative to non-primes, with C and W showing the strongest relative enrichment (Additional file 1: Fig. S7e).

### Absent kmers in the genome or proteome can serve as a phylogenetic classifier

**Fig. 7:**
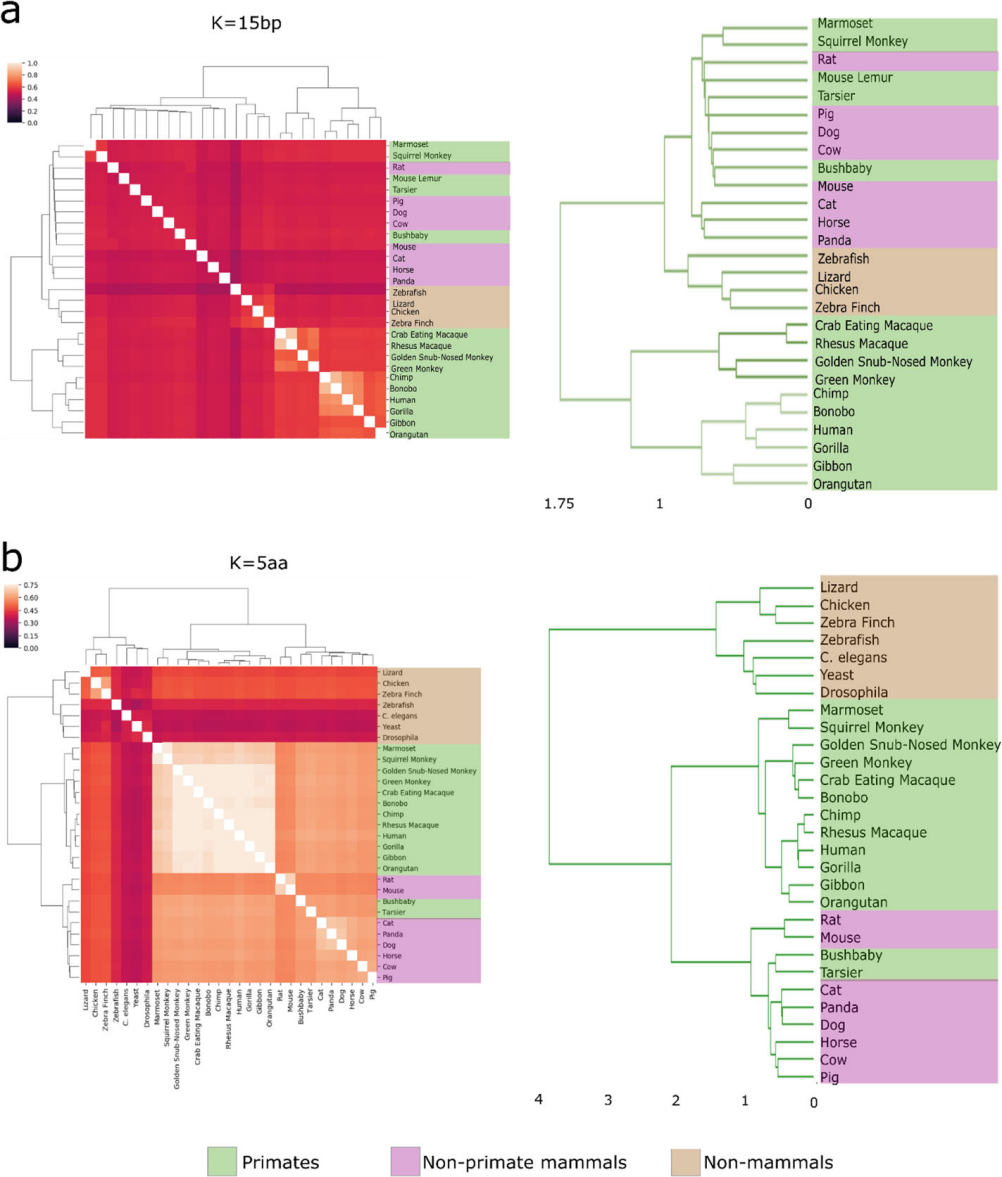
Evolutionary relationship of nullomers across 27 species and of nullpeptides across 29 species. **a, b** Hierarchical clustering displaying the Jaccard index based on genome nullomers and nullpeptides shared between pairs of species and the associated phylogenetic dendrogram for *K* = 15 bp (**a**), *K* = 5aa (**b**). Colors in the dendrograms represent primate (light green), non-primate mammals (light red), and non-mammals (brown)

Phylogenetic trees are usually built based on similarities and differences of existing sequences. Here, we wanted to test whether these trees could be built in an appropriate manner based on the absence of DNA and peptide sequences, i.e., nullomers and nullpeptides. We thus utilized our annotated nullomers and nullpeptides from the 30 eukaryotic species to build phylogenetic trees. In accordance with previous work that has shown clustering of species based on nullomers and first-order nullomers [2, 18, 19], we obtained an overall expected tree structure when clustering based on the Jaccard index, which accordingly clustered together primates, mammals, and all the other organisms (Fig. 7a, Additional file 1: Fig. S10). Of note, by definition, genomes with widely different sizes will inevitably have a very low Jaccard similarity score. Although these differences make it difficult to interpret the results, we consider this consistent with their biological differences. The phylogenetic tree greatly improved with the length of the nullomer used. For example, we observed a significant improvement for nullomers *K* = 15 compared to *K* = 12 (Fig. 7a, Additional file 1: Fig. S10a) and nullpeptides *K* = 5aa versus *K* = 4aa (Fig. 7b, Additional file 1: Fig. S10b), likely due to having increased numbers of sequences

## Discussion

Nullomers and nullpeptides are intriguing sequences whose absence in the genome or proteome could be due to their deleterious effect on the organism. Here, we characterized these sequences in the reference human genome and proteome and within specific functional categories. The latter focused on coding sequences, exons, introns, 5′UTR, 3′UTR, promoters, and other noncoding functional elements, and was unique, as only looked at nullomers within these functional units. We observed that coding (CCDS) regions have a higher density of potential nullomer-resurfacing mutations (Fig. 3c), when compared to the other functional units. This is likely a result of protein coding sequence restrictions that are less flexible compared to noncoding gene regulatory sequence constrictions. However, the high enrichment of nullomers in promoters and enhancers suggest that many may have important regulatory roles, and this could be followed up with functional assays. In accordance with previous studies [1–3] and using different approaches, we show that nullomers and nullpeptides are under negative selection. We show that interindividual nucleotide variation can lead to the materialization of nullomers and that a subset of nullomers are population-specific. We also observed that the vast majority of nullomers and nullpeptides do not resurface or resurface with a low probability in the human population through common variants. The subset of nullomers and nullpeptides that frequently resurface due to common variants are likely not deleterious. Moreover, utilizing an additional 29 genomes and 28 proteomes from diverse species, we annotated both nullomers and nullpeptides that are shared among clades (primates vs. non-primates) and ones that are unique to each species (e.g., human-specific). We show that this annotation can be used to build phylogenetic trees that are similar to those using existing sequences. To date, only three nullpeptides: WCMNW, NWMWC, and WFMHW, have been tested for their effect on cell growth and apoptosis, and it was shown that the former two had an impact on cells [5, 6]. In our analyses, these three sequences were all absent from the 30 species analysis, but were found 100, 93, and 241 times in the UniParc dataset in other organisms. Additionally, we found that the WCMNW pentapeptide could be generated only by one single nucleotide substitution throughout the human CCDS. Recent work has also shown that a quarter of MAW peptides resurfacing due to a single change could be harmful and that several of these could lead to the formation of a phosphorylation site [3]. Utilizing our repository and combined scoring criteria and expanding them by also considering nullomer length, species, and genomic functional subsets could assist in identifying nullomers and nullpeptides that impact organismal fitness, through various possible mechanisms resulting in this phenotype.

Previous studies have all focused on reference sequences and whole genome analysis when defining nullomers [2, 3, 19]. Here, we took into account natural human variation, finding that a significant proportion of nullomers are actually present in a subset of individuals, and in some cases associated with common variants. Building on this finding, we show that nullomers could be utilized to distinguish between specific human populations and between species. A carefully selected panel of nullomers could potentially be used to distinguish subpopulations. We also discovered numerous species-specific nullomers and nullpeptides. As these sequences do not exist in the other populations/species, development of tools that screen for these sequences could provide a rapid test to determine the presence or absence of populations/species, e.g., for metagenomic studies. It will be intriguing to test the functional properties of these nullomers/nullpeptides, if they exist, and whether they might have any mechanistic effects on specific phenotypes.

Phylogenetic trees are routinely constructed using existing DNA sequences, for example 16S. However, these can be complicated to construct due to horizontal/lateral gene transfer, the transfer of genetic material between unrelated organisms [20]. The use of sequences that are absent in some genomes might facilitate tree construction. While the transfer of sequences between organisms will also affect the genetic makeup of sequences that are absent, i.e., nullomers and nullpeptides, this effect might be less pronounced and as such sequence absence might pose as a useful tool to build these trees. It would also be interesting to carry out phylogenetic classifiers that use a combination of both existing and absent sequences (i.e., nullomers and nullpeptides) to see if these might improve these classifiers. Combined, our results suggest that nullomers and nullpeptides can assist in generating phylogenetic classifiers.

## Conclusions

In summary, our work provides a list of missing DNA and amino acid sequences in over 30 genomes and in seven different functional categories. It also provides various scoring metrics to rank their potential deleterious effect on the organism and suggests that both nullomers and nullpeptides are subject to negative selection. It will be interesting to check the functional effects of these sequences in the various categories. While coding nullomers may be more straightforward, it will be extremely intriguing to decipher the function of these sequences in the noncoding space. Past work has analyzed the occurrence of 7 bp sequences in 11,257 whole human genomes to identify constrained noncoding regions and shows that they are enriched for pathogenic variation [21]. While there are no existing 7 bp nullomers, it would be interesting to see whether these constrained sequences are also more constrained against nullomers, and if so, how might these missing sequences affect their function when introduced.

## Methods

### Nullomer and nullpeptide identification

We developed an algorithm in Python that performs an exhaustive search across input nucleotide sequences and identifies the number of occurrences of each kmer for a selected range of kmer lengths (Fig. 1a). The set of all possible kmers is used to compare against and deduce all nullomers for the input nucleotide sequences. The algorithm can also be used to identify nullpeptides using the standard twenty-amino acid code, and it ignores any rare amino acids such as selenocysteine. Here, we ran the algorithm for nucleotide lengths up to 15 base pairs (bp) and peptide lengths up to 7 amino acids (aa) from which we derived the list of nullomers and nullpeptides in the reference human genome (hg38) and proteome (UP000005640), respectively.

To assign functional categories in nucleotide nullomers, we utilized the GENCODEv28 annotation [22] to identify genic and non-genic sequences, with the former being defined as the sequence from the transcription start site (TSS) to the transcription end site (TES). The genic portion was further broken down to 5′UTR, exonic, intronic, 3′UTR, and consensus coding sequences (CCDS). Candidate cis-Regulatory Elements were extracted as defined by ENCODE using the reference dataset and the cell-type-specific datasets of K562 and HEPG2 cell lines [10, 11]. For the reference dataset, the categories included promoter-like, proximal and distal enhancer-like, CTCF-only and CTCF-bound, and DNase-H3K4me3 sequences, whereas for K562 and HepG2 cell lines the sub-classifications included promoter-like, proximal enhancer-like, distal enhancer-like, CTCF-only, low-DNase, and DNase-only sequences. Transposable element analysis in the human genome was performed using RepeatMasker (Smit Hubley Green, www.repeatmasker.org).

A nullomer of order *i* indicates that any base substitutions in *i* places along the kmer still result in a nullomeric sequence. Consequently, nullomers of order *i + 1* are also nullomers of order *i*. First-order nullomers in the human genome and its functional subdivisions were subsequently identified. For each nullomer, the algorithm compared the number of occurrences of all possible kmers of one base pair Hamming distance. If the sum of the occurrences of all possible kmers across the search space was zero, the nullomer was scored as a first-order nullomer.

### Analysis of nullomers across diverse organisms

Nullomer extraction was performed across a range of species using their reference genomes and proteomes (Additional file 2: Table S1). We prioritized all primate species with a good publically available reference genome: chimp, bonobo, gorilla, gibbon, gray mouse lemur, crab-eating macaque, green monkey, golden snub-nosed monkey, rhesus macaque, Sumatran orangutan, common marmoset, northern greater galago (bushbaby), Philippine tarsier, and black-capped squirrel monkey. We added the following additional mammals: pig, horse, cat, dog, and cow; and the following rodents: mouse and rat. Finally, we expanded our search beyond mammals to include the house chicken, zebra finch, zebrafish, drosophila (*D. melanogaster*), lizard, nematode (*C. elegans*), and yeast (*S. cerevisiae*). For mouse lemur, a reference proteome was not available and thus it was excluded from the nullpeptide analysis.

UniProt^10^, which represents a comprehensive and non-redundant database of all known protein sequences across all biological organisms, was used for our nullpeptide and peptide prime analyses. It was downloaded on October 8th, 2019, and at the time contained 1,030,456,800 protein sequences, spanning 98,369,395,754 amino acids.

### Statistical evaluation of nullomers

Expanding on the notion of nullomers of order 1 or higher, we developed a set of statistical methods to prioritize nullomers and as metrics of potential negative selection (Fig. 2).

For the first metric, *φ*1_,_ we examine all possible 1 bp substitutions of a nullomer *N*. Then, we calculated how many times the resulting kmer occurs in the genome. Finally, we define *φ*1 for the given nullomer as the mean number of appearances of the resulting nullomers over all possible substitutions:

$$ {\phi}_1(N)={\sum}_{i=1}^k\left({\sum}_{j=\left\{A,T,C,G\right\}\setminus {N}_i}A{N}_{i,j}\right)/3k $$

where *k* is the length of the nullomer *N*, *N*_*i*_ is the *i*th bp of nullomer *N* and *AN*_*i*, *j*_  is the number of appearances of the kmer that results by substituting the *i*th bp of N by *j*.

A higher value of *φ*1 might signify a stronger negative pressure at play to avoid the nullomer occurring in the genome. Because the variance in the frequency of each amino acid usage in the proteome is large, for peptides we used a variant of *φ*1 based on the mean of all possible permutation occurrences of a nullpeptide rather than for all possible 1 bp substitutions.

Our second score metric (*φ*2) is based on a 100-fold Monte Carlo simulation permuting each chromosome of the human genome, or each sequence in the simulated subcompartment, controlling for mononucleotide, dinucleotide, or trinucleotide content for each simulation (*n* = 100 simulations were performed in each case). Simulations were performed using the Ushuffle package [23]. By permuting the genome or its genomic subcompartments, we address issues associated with the intrinsic rarity of GC-rich kmers in the human genome. We define *φ*2 as the mean number of appearances of nullomer *N* in our permuted genome sequences.

$$ \varphi 2(N)={\sum}_{i=1}^{100}\left({N}_i\right)/100 $$

where *N*_*i*_is the number of appearances of nullomer *N* in the *i*th simulation.

Our third metric adds an evolutionary perspective to the score. Strong natural selection against a sequence will be implicated by the absence from the genomes of many species. We define the species-based nullomer score *φ3* as centered on the occurrence of a kmer motif in the genome of different species (see above) and it is defined as the ratio of species other than human that present this human nullomer in their genome. In our work, we looked at 29 species, in addition to humans.

$$ \varphi 3(N)=M/n $$

where *M* = number of species that include nullomer N, *n* = total number of species examined.

Furthermore, for each of the metrics above, we created a sorted list of nullomers according to their score in ascending order, as we postulate that a lower score in one of the three metrics indicates a higher likelihood of negative selection. In order to identify the nullomers that are most likely candidates for negative selection, we defined the aggregate metric *φN* as the average rank of nullomers in the three sorted lists. We hypothesize that the nullomers with the lowest *φN* score are those under strong negative evolutionary pressure.

$$ \varphi N(N)= RANK\left(\varphi 1(N)\right)+ RANK\left(\varphi 2(N)\right)+ RANK\left(\varphi 3(N)\right) $$

where RANK(φi(N)) is the position of nullomer N in the list of all identified nullomers sorted in ascending order according to their φi values. In case several nullomers share the same value at a specific score, their ranking will be the same as well per that specific scoring metric.

### Population variation and materialization of nullomers

The short human variation annotation file for the human genome was downloaded from (https://storage.googleapis.com/gnomad-public/release/2.1.1/liftover_grch38/vcf/genomes/gnomad.genomes.r2.1.1.sites.liftover_grch38.vcf.bgz). We downloaded the gnomAD data from https://gnomad.broadinstitute.org/. The population breakdown of variants was performed for African /African-American (AFR), Amish (AMI), Latino / Admixed American (AMR), Ashkenazi Jewish (ASJ), East-Asian (EAS), Finnish (FIN), Non-Finnish European (NFE), and South Asian (SAS) as defined by the 1000 Genomes consortium [10]. A custom Julia script was used to identify every single base pair deletion, insertion, and substitution that can result in the creation of a kmer which was designated as a nullomer based on the reference sequence. The list of nullomer creating substitutions was compared to the naturally occurring variants annotated by the gnomAD consortium to calculate the probability of individual nullomers resurfacing.

The density of putative nullomer and nullpeptide materializing mutations was calculated across genic regions, at CCDS regions and for each protein. Gene ontology analysis (see details in section below) was performed for the top 10% and bottom 10% of genes in each case, with *K* = 11–13 bp for nullomers and *K* = 4 and *K* = 5 for nullpeptides. A reactome analysis was also performed in each case.

### Phylogenetic analyses of nullomers and nullpeptides

Nullomers were identified separately for each species and for each kmer length. The Jaccard index, which is calculated as the size of the intersection of shared nullomers between a pair of species divided by the size of the union of nullomers in the two species, was used as a similarity metric for the construction of phylogenetic trees. Phylogenetic trees were constructed in Python using the package “scipy” [24] and the functions “dendrogram” and “linkage” using Ward’s method as a criterion. The package “seaborn” and the function “clustermap” were used to construct the hierarchical clustering dendrograms and the associated heatmaps using Ward’s method. The same analysis was performed for the construction of phylogenetic trees using sequences.

### Gene Ontology (GO) analysis

For each gene (transcript), the number of possible nullomer-resurfacing mutations was divided by the transcript length to yield a “density” measure. When several transcripts were available per gene according to the ENSMBL transcripts annotation, the transcript (isoform) presenting the highest density of mutation of all the gene’s annotated transcripts was picked and carried over. Each gene was represented by a single density value. Gene Ontology enrichment was done using clusterProfiler::enrichGO() R package [25] and using the ReactomePA:enrichPathway() R package [26]. For the REACTOME database [17] analysis, significance threshold was set as q-value < 0.05. We created two sets per sample—the top 10% densest genes and the bottom 10% least dense genes. For visualization purposes, we focused on the 10 enriched terms in either analysis, and used either the top 10% most common nullomer-resurfacing mutation dense genes or the bottom 10% less dense ones. Redundant terms were removed for visualization purposes and the term with the lower *q*-value was kept and displayed.

The algorithms used in this manuscript can be accessed at:

https://github.com/Ahituv-lab/Nullomers or https://zenodo.org/record/5148239 [27]

## Supplementary Information

**Additional file 1.**

**Additional file 2.**

**Additional file 3.**

## Data Availability

The datasets supporting the conclusions of this article are included within the article (and its additional files). Additionally, the nullomer datasets generated for this manuscript will be available at: https://pharm.ucsf.edu/nullomers
