# Supplementary material for: Absent from DNA and protein: genomic characterization of nullomers and nullpeptides across functional categories and evolution
Source: Genome Biol. 2021 Aug 25;22:245. doi: 10.1186/s13059-021-02459-z (PMC8386077; doi:10.1186/s13059-021-02459-z)
Supplement: Supplementary file 1 — Additional file 1. [file 13059_2021_2459_MOESM1_ESM.docx]

**Supplementary Material**

**
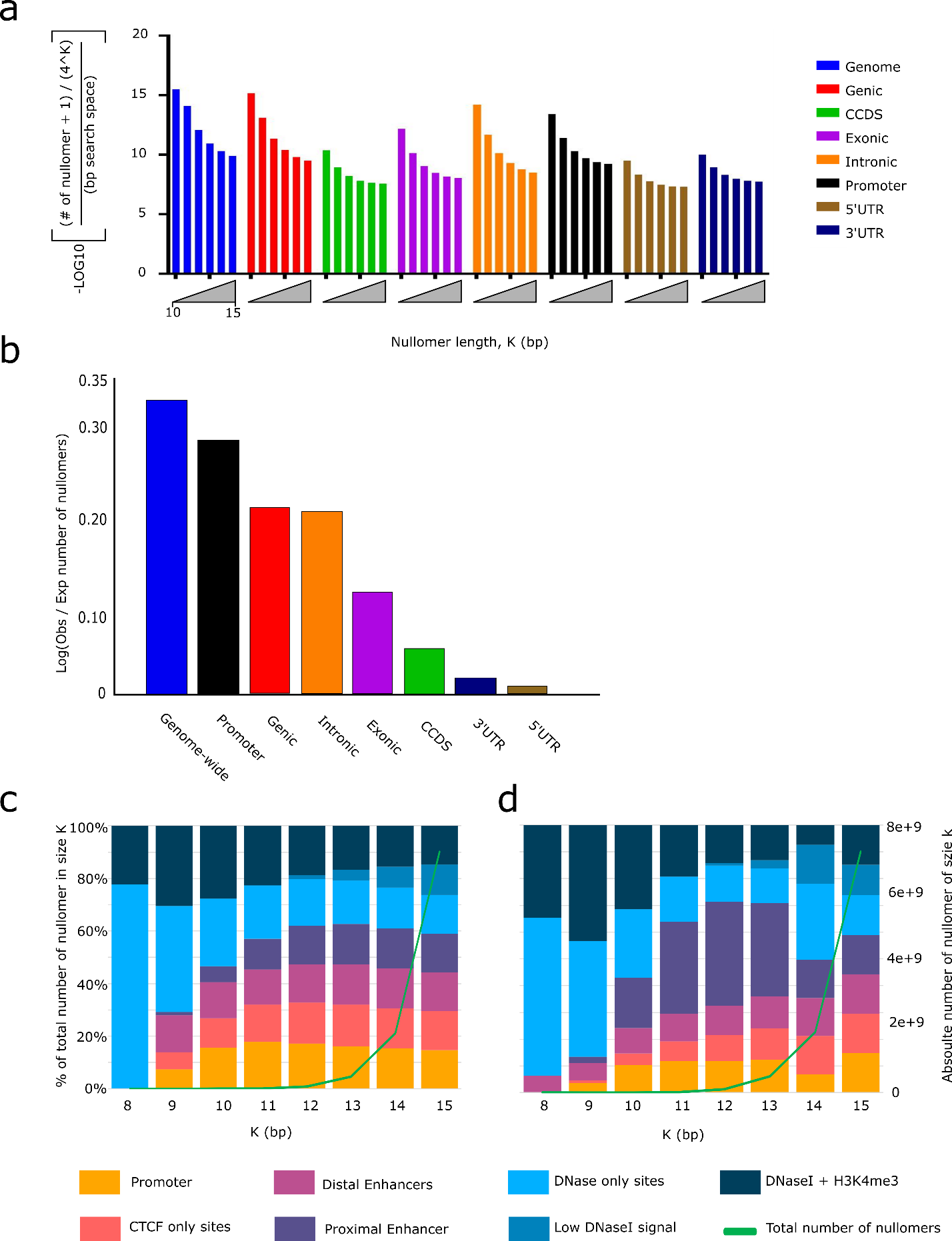
**

**Fig. S1 Distribution of nullomers in the human genome by functional subcompartments.** a Proportions of kmers annotated as nullomers corrected for the overall kmer population at sike K bp, and for the subcompartment total search space (in bp). The calculation is made as: -log10 [ (# of nullomers of K bp in search space X +1) / (4^K) ] / [bp search space]. **b** $\varphi2$metric score associated enrichment of nullomers relative to simulations in the genome and various functional categories across K=10-15 bp**. c-d** Proportion of kmer space for nullomers K=10-15bp in the human genome and its functional sub-compartments HepG2 (**c**) and K562 (**d**) cells.

**
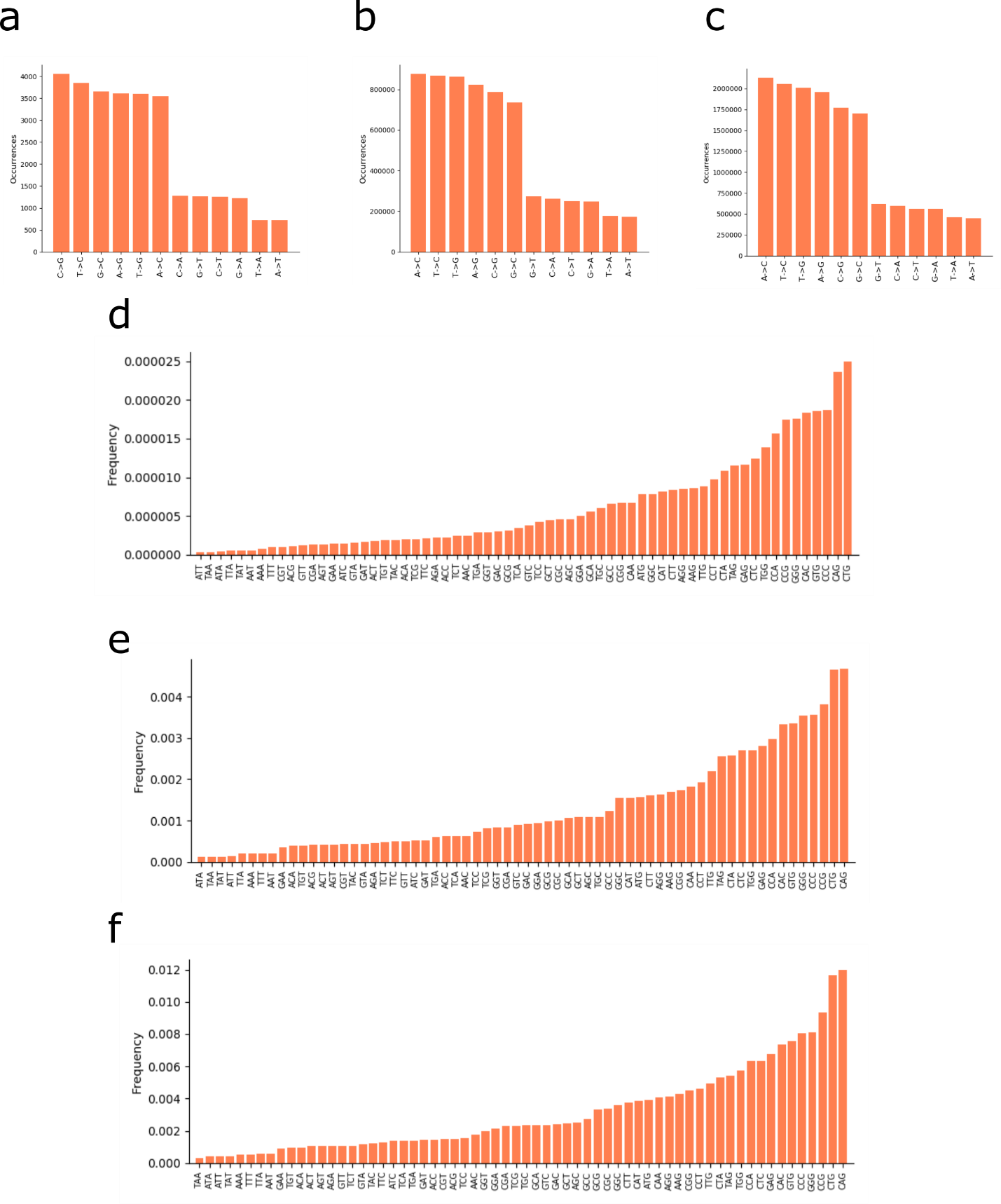
**

**Fig. S2 Nucleotide patterns at mutation sites that lead to the materialization of nullomers in the human genome. a-c** The number of occurrences for each substitution type for nullomer lengths of K=11 (**a**), K=12 (**b**), K=13 (**c**). **d-f** Frequency of trinucleotide context of substitutions for nullomer length K=11 (**d**), K=12 (**e**), K=13 (**f**) controlling for trinucleotide composition of the human genome.


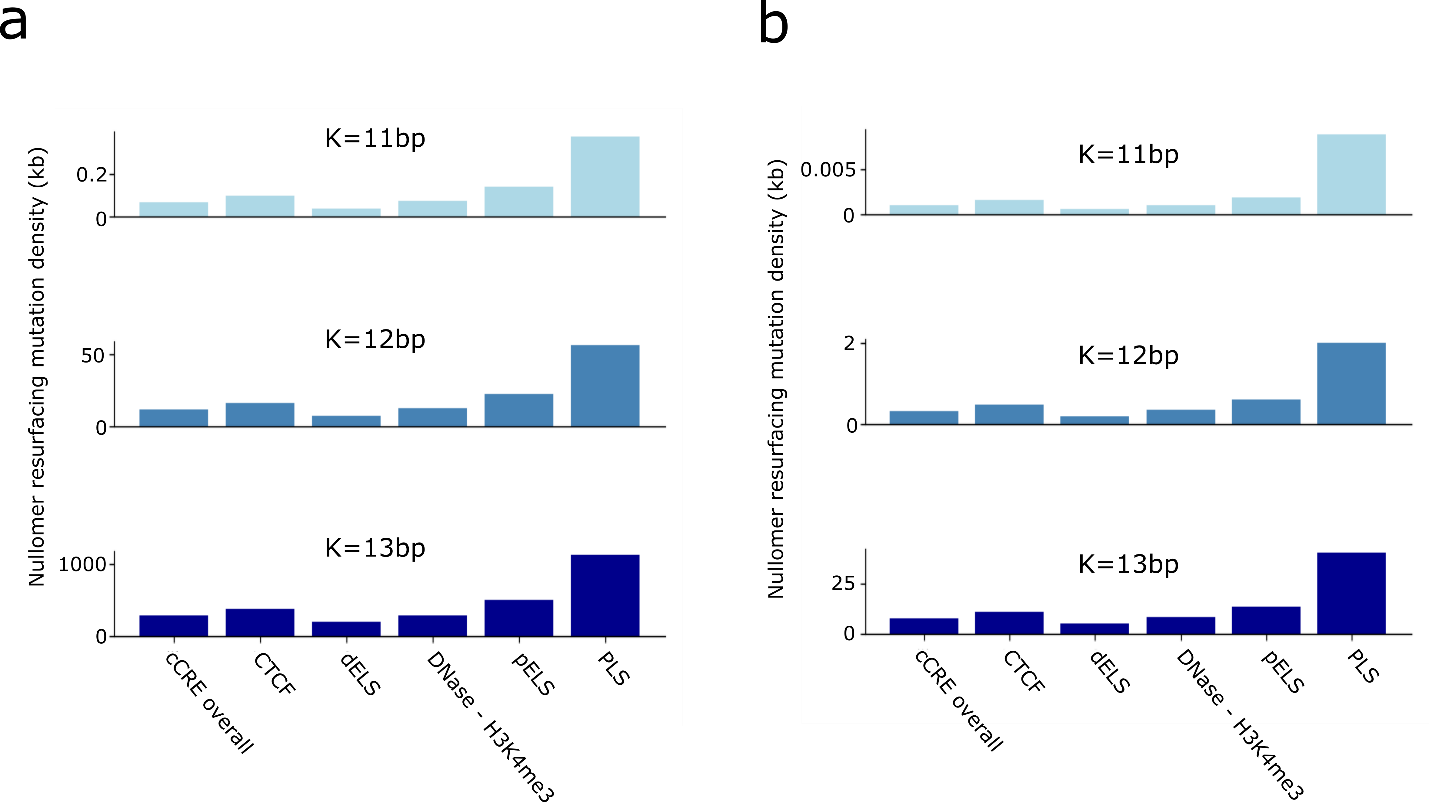


**Fig. S3 a** Density of putative mutations that can materialize nullomers across ccRE categories for K=11-13bp nullomer lengths. **b** Density of human population variants that can materialize nullomers across ccRE categories for K=11-13bp nullomer lengths. PLS corresponds to promoter-like signature, pELS to proximal enhancer-like signature, dELS to distal enhancer-like signature, DNase H3K4me3 to DNase and H3K4me3 sites and CTCF to CTCF-only and CTCF-bound sites.


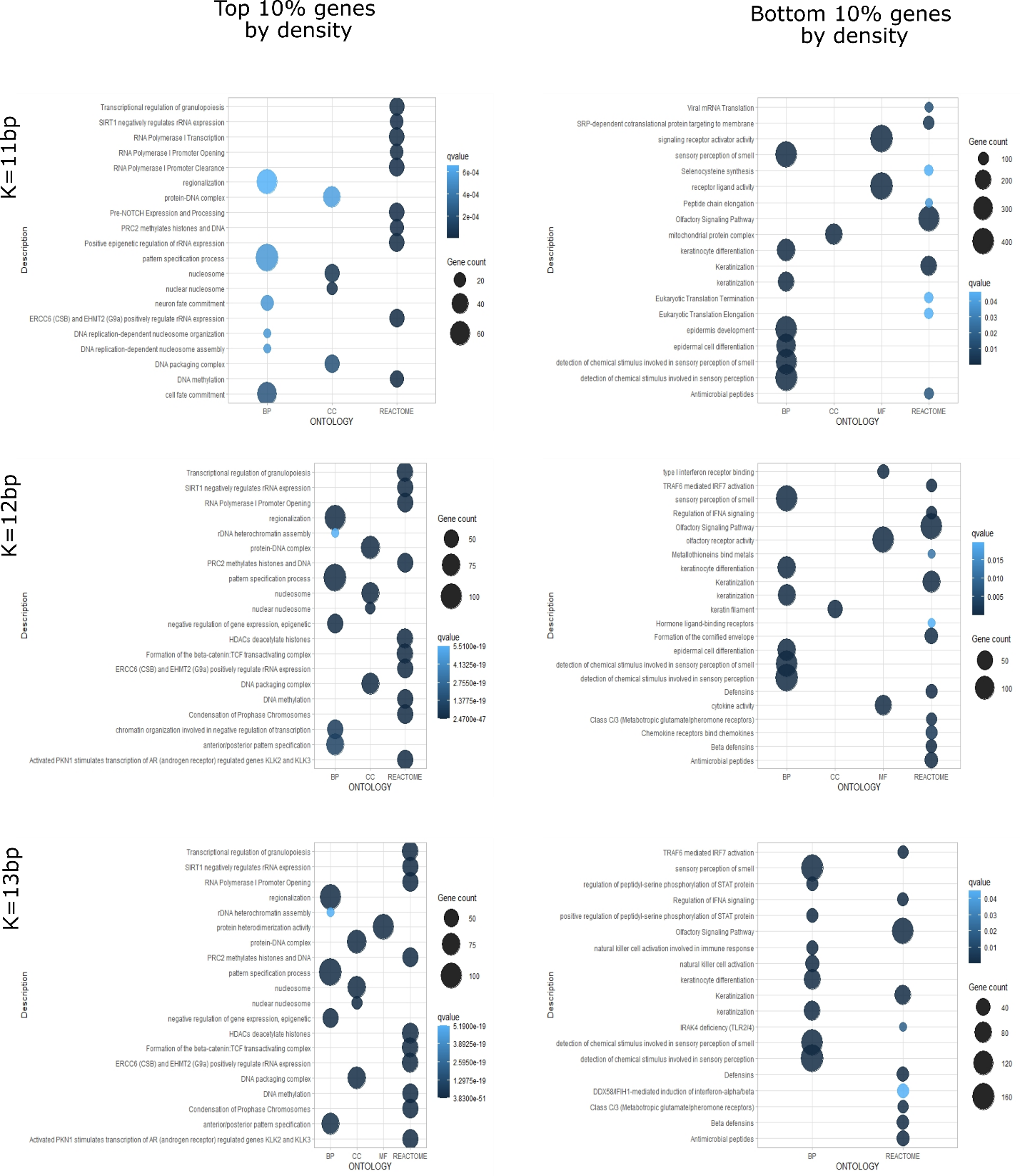


**Fig. S4** GO term analysis of top 10% and bottom 10% of genes according to the density of putative nullomer resurfacing mutations for nullomer lengths of K=11, K=12 and K=13 bps. GO term analysis is performed across Biological processes (BP), Molecular Functions (MF) and Cellular Compartments (CC). Pathway analysis is performed with the REACTOME database for gene set enrichment.

**
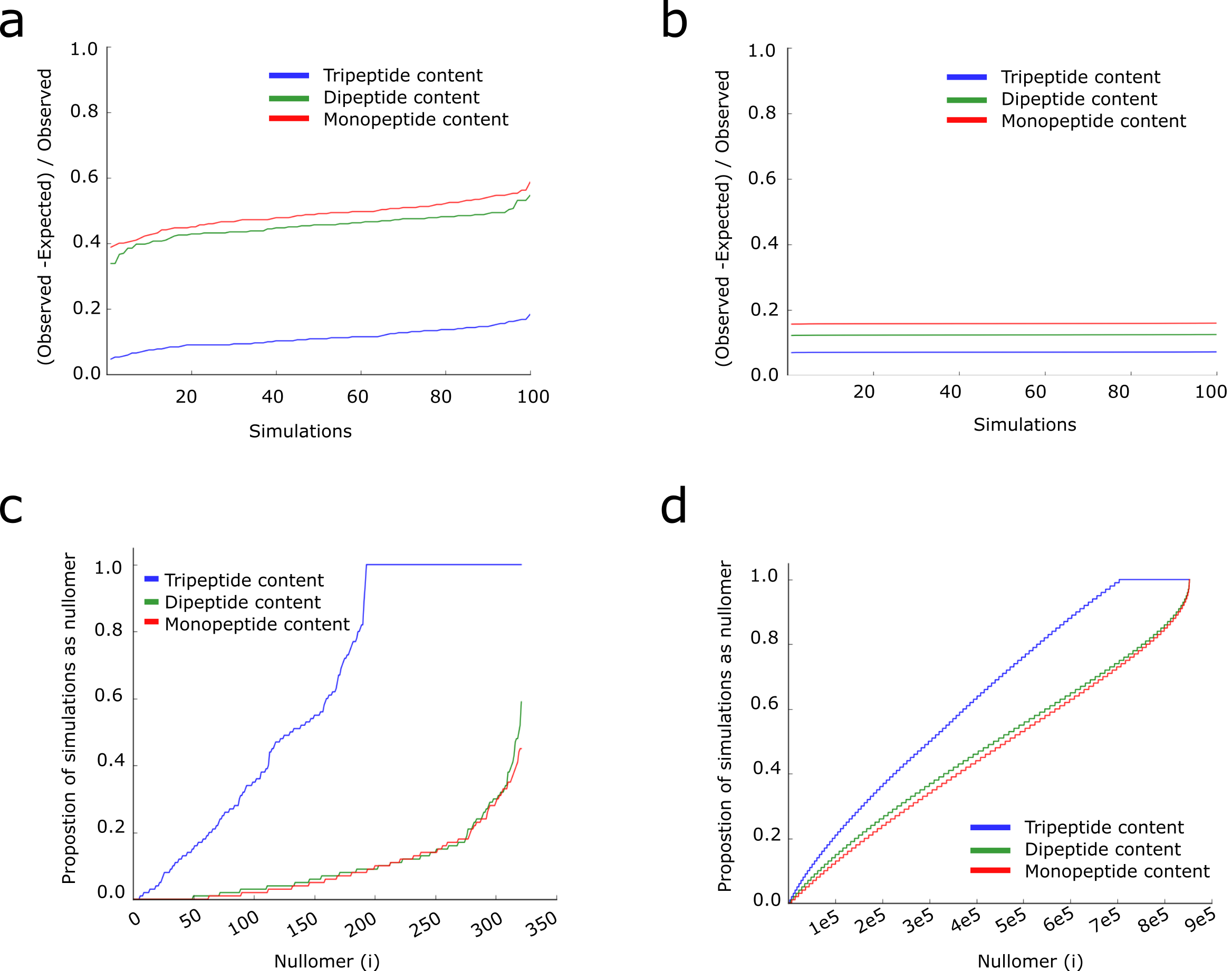
**

**Fig. S5 A higher number of nullpeptides is found in the human proteome relative to those found in simulated proteomes. a-b** Ratio of (Observed-Expected) / (Expected) nullpeptides across the simulations for 4 aa (**a**) and 5 aa (**b**). **c-d** Proportion of simulations in which each of the nullpeptides was observed for 4 aa (**c**) and 5 aa (**d**).


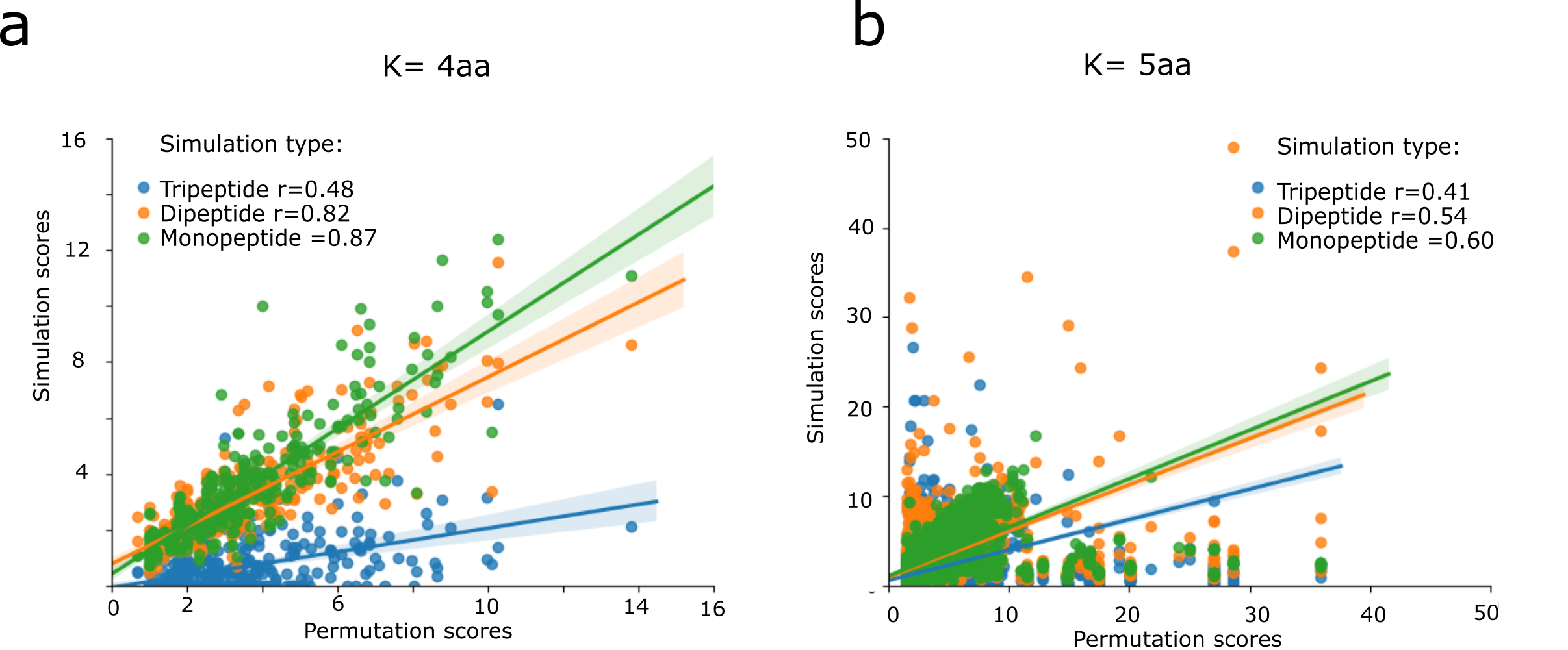


**Fig. S6 Association between permutation and simulation scores in nullpeptides. a** The legend shows the Pearson correlation between the number of occurrences in permutations and combinations for each nullpeptide of 4aa. **b** The legend shows the Pearson correlation between the number of occurrences in permutations and combinations for the 10% of nullpeptides of 5aa that were least often found as ranked by mean permutation occurrences.

**
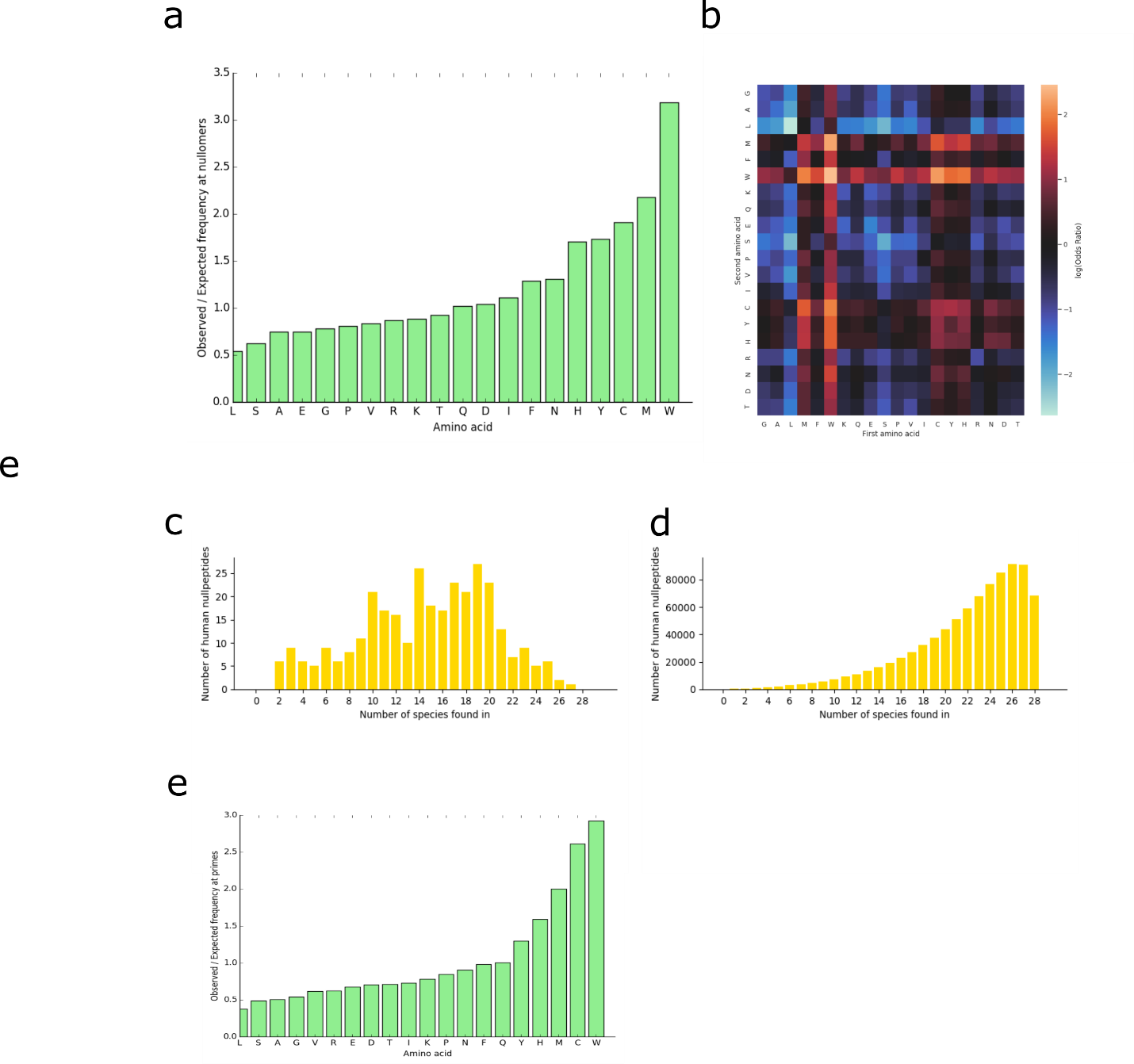
**

**Fig. S7 Human proteome nullpeptides and nullpeptide primes and their characteristics at the protein level.** **a** Enrichment of amino acids in nullpeptide sequences compared to their frequency in non-nullpeptide sequences. **b** Biases in dipeptide usage in nullpeptides relative to non-nullpeptides in the reference human proteome. **c-d** Number of species in which human nullpeptides of 4 (**c**) and 5 (**d**) amino acids length were identified. **e** Enrichment of amino acids in prime sequences relative to non-prime sequences from UniParc database.


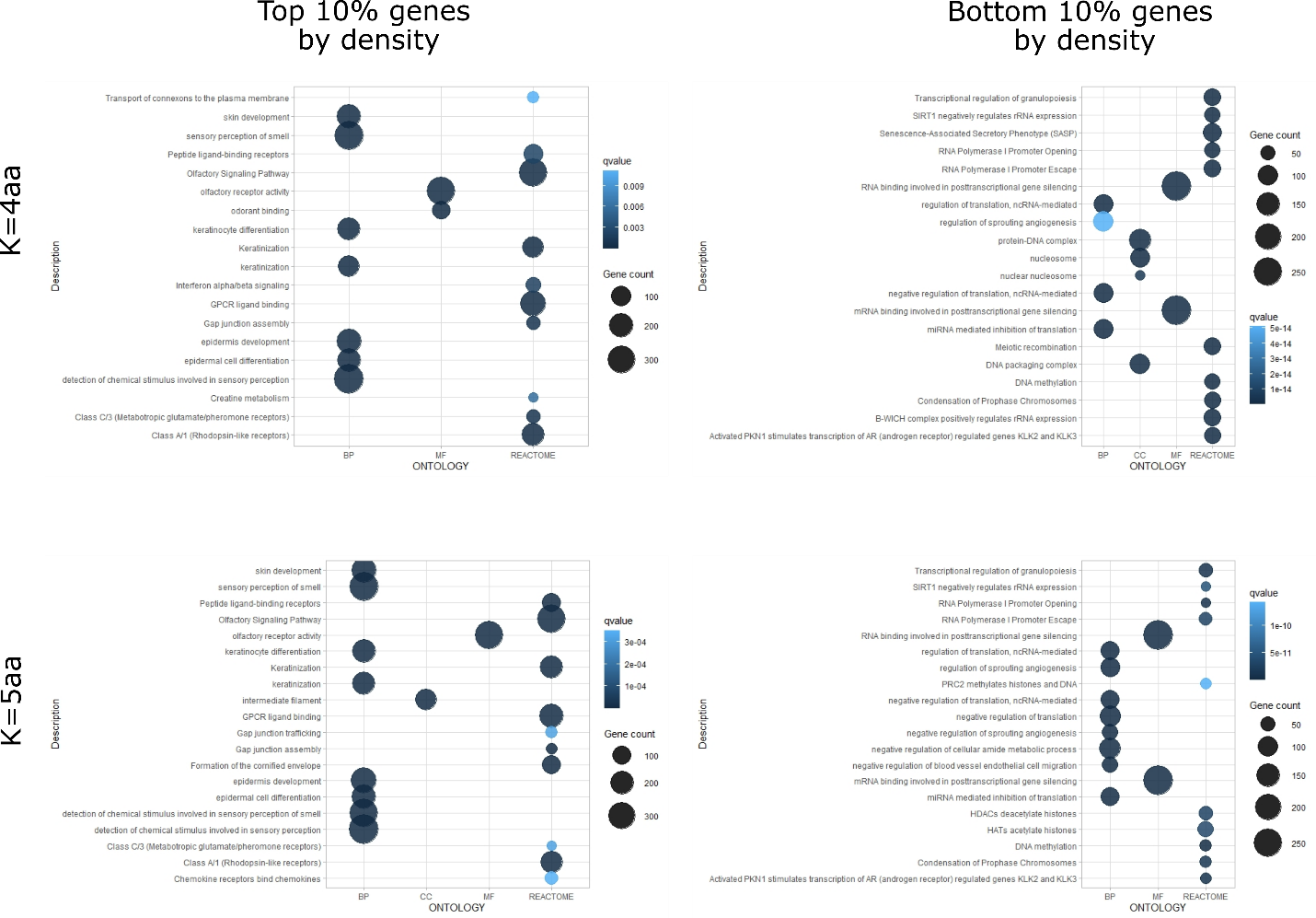


**Fig. S8** GO term analysis of top 10% and bottom 10% of genes according to the density of putative nullpeptide resurfacing mutations for nullpeptide lengths of K=4aa and K=5aa.

**
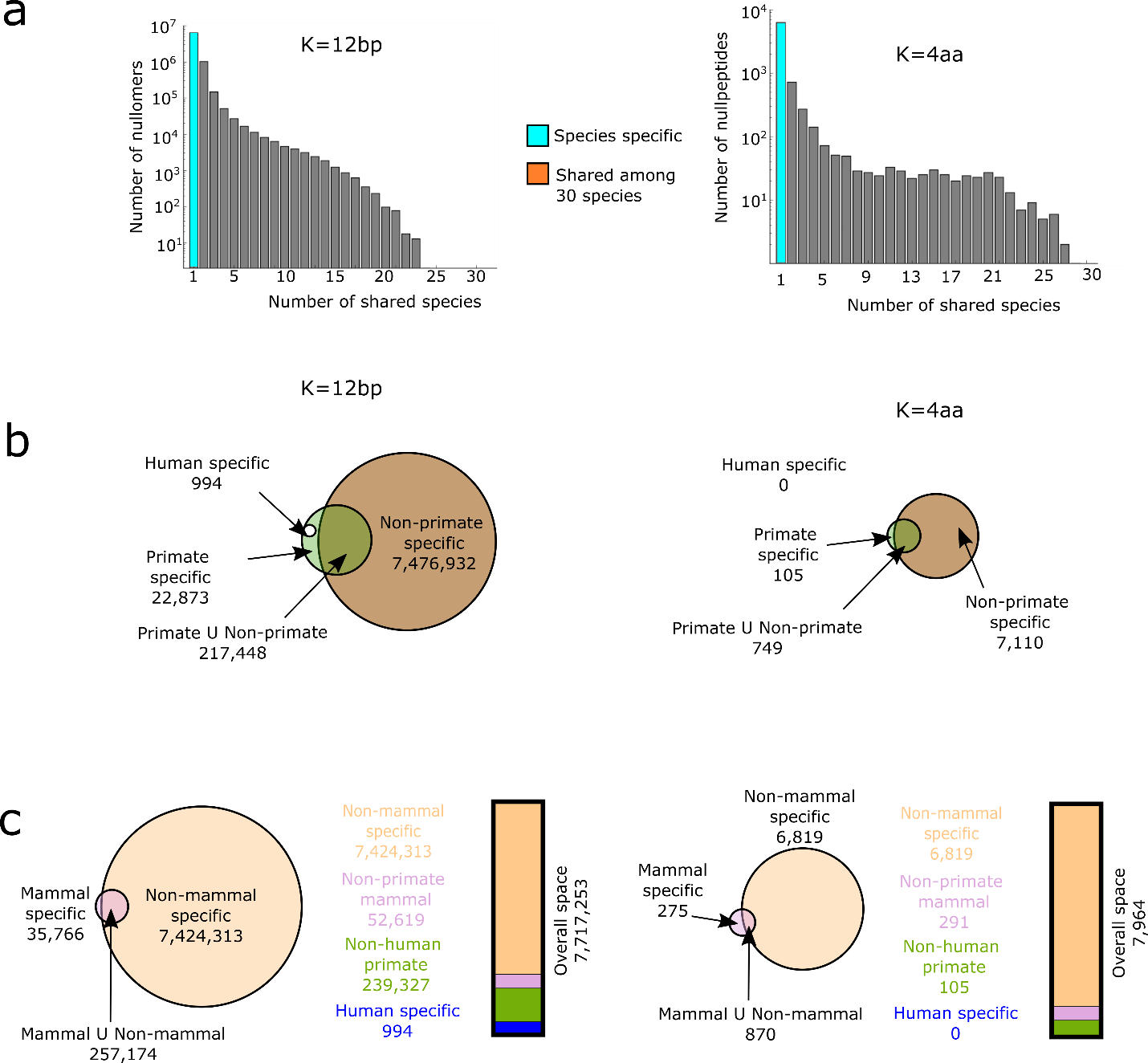
**

**Fig. S9 Characterization of nullomers and nullpeptides across species. a** Number of species sharing nullpeptides for K=12 base pairs (left) and K=4 amino acids (right). Number of species-specific nullpeptides are colored in turquoise and nullpeptides shared across species are shown in orange. **b** Venn diagrams displaying the number of nullomers (left) and nullpeptides (right) shared between humans, other primates and non-primates species analyzed. **c** Intersection between mammalian and non-mammalian species nullomers (left venn diagram) and nullpeptides (right venn diagram); adjacent to each is a staggered bar plot with cumulative number of nullomers/nullpeptides. Intersection of nullomers between humans, all other primates aside from human, all mammals beside primates and all species. The diameter of the circles and bar heights are roughly correlated to differences in numbers.

**
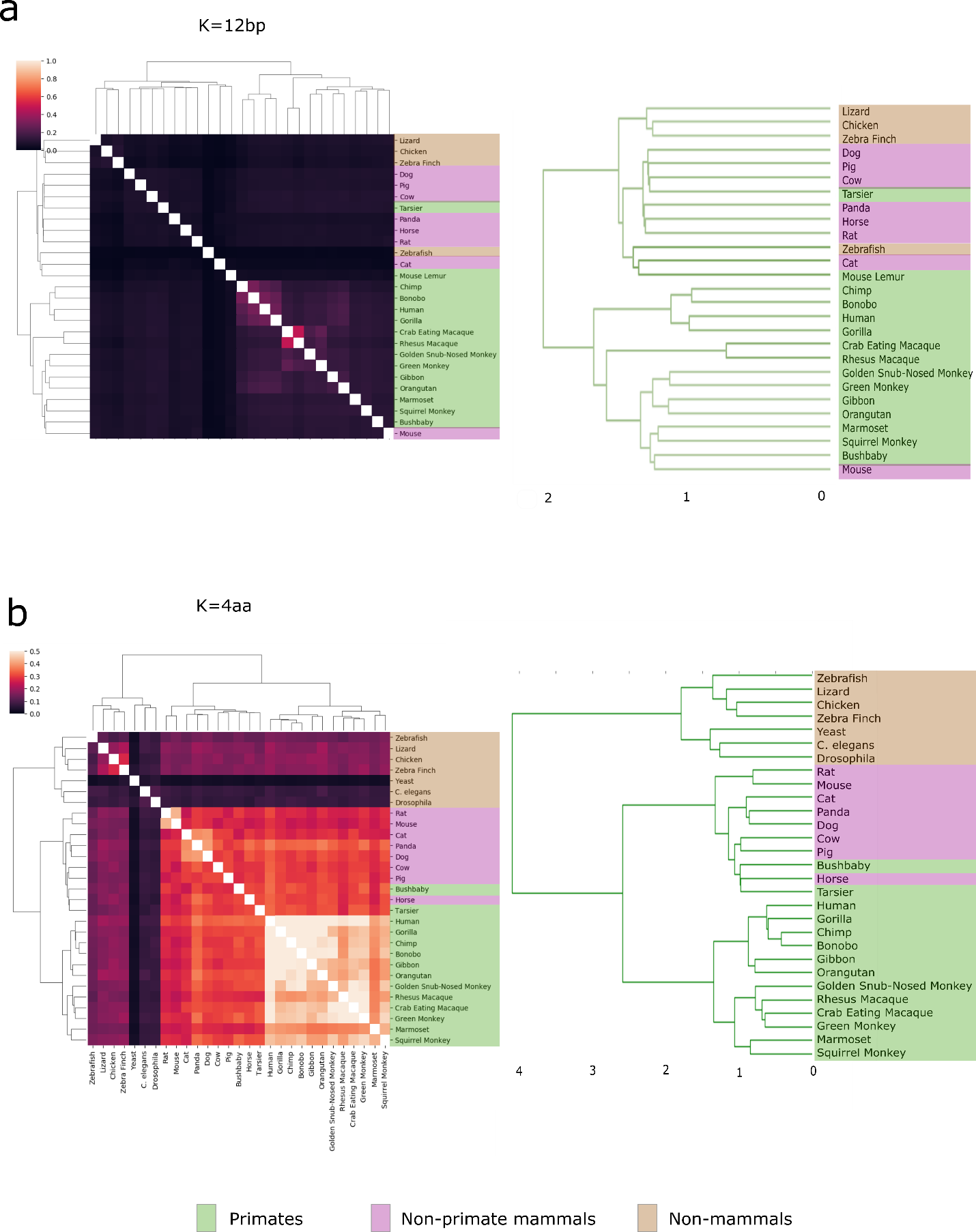
**

**Fig. S10 Evolutionary relationship of nullomers across 30 species and of nullpeptides across 29 species. a** Hierarchical clustering of nullomers (length K=12bp**)** for 30 eukaryotic species. **b** Hierarchical clustering of nullpeptides (length K=4aa) for 29 eukaryotic species. Similarity between pairs of species was calculated using the Jaccard index.
