# Supplementary material for: Absent from DNA and protein: genomic characterization of nullomers and nullpeptides across functional categories and evolution
Source: Genome Biol. 2021 Aug 25;22:245. doi: 10.1186/s13059-021-02459-z (PMC8386077; doi:10.1186/s13059-021-02459-z)
Supplement: Supplementary file 3 — Additional file 3. [file 13059_2021_2459_MOESM3_ESM.docx]

**Review history**

**First round of review**

**Reviewer 1**

Georgakopoulos-Soares and Barnea et al. analyze the distribution of nullomers and nullpeptides, DNA and protein sequences that are absent from one or more genomes and proteomes, in the human and other reference genomes. They use several metrics to support that nullomers and nullpeptides may be excluded from genomes due to negative selection. They also identify nullomers and nullpeptides in the human genome that could be formed by single mutation events (which for the purposes of this review, I’ll refer to as “proto-nullomers”), and show that nullomers can be reintroduced to extant human genomes via DNA polymorphisms. They then consider the distribution of shared nullomers across genomes, and identify an intriguing set of nullomers, which they term primes, that are absent from all of the genomes they analyzed.

Finally, they demonstrate how patterns of nullomer retention or loss can be used for phylogenetic classification.

This is an intriguing, conceptually novel study that brings attention to a phenomenon that has been somewhat neglected in comparative genomics and analyses of genome evolution. Genomes and proteomes are large, but not infinite, so it would not be surprising to identify particular sequences (especially longer sequences) that do not appear in a given genome. However, the authors’ analyses overall support that at least a subset of nullomers are restricted from genomes due to selection rather than random chance. The catalog of nullomers and nullpeptides the authors have assembled will also enable functional studies of these understudied sequences.

Despite my enthusiasm, I do have several comments on the analysis and presentation of the results:

1. I was disappointed that the authors did not consider the exclusion of nullomers in gene regulatory sequences. In their discussion, they say this would be an interesting analysis (mentioning conserved noncoding sequences specifically), but don’t pursue it here. There is now an abundance of highly resolved functional genomic data that could be exploited to identify nullomers that are absent from regulatory elements. For example the ENCODE v3 data includes DNase footprint data from hundreds of human tissue samples and cell lines. These footprints are longer than the nullomers they are considering here, and also include predicted TF binding site data. Identifying the subset of nullomers that are excluded from these sites would be interesting. I’d also like to know how frequent “proto-nullomers” are in regulatory space, whether they correspond to specific TF motifs, and how often they are converted to nullomers by mutation.
2. The potential functional impact of nullomers (in UTRs or promoters, for example) or nullpeptides is not explored in much depth. For example, the authors note that “Additionally, we found that the WCMNW pentapeptide could be generated only by one single nucleotide substitution throughout the human CCDS.” This raises the question of which proteins are sensitive to nullpeptide formation due to variation? Do they converge on specific functions, and/or do such proto-nullpeptides tend to fall in particular protein-coding domains? Are variants that convert proto-nullomers/proto-nullpeptides to nullomers/nullpeptides more likely to lead to repeat reactivation or gene disruption?
3. The presentation of the results and methods is a bit confusing, and needs to be clarified. This is particularly important for this paper, since the authors are analyzing the *absence* of sequences

from the genome. This requires the reader to – in a way – think “backwards” and as such they could get tripped up. I point out specific examples below.

- 1. The terminology “nullomer conservation” is a bit confusing. I take it to mean that the same nullomer is absent from the specified genomes, but some readers may not get this.
  2. It would help in Table 1 to show the proportion of nullomers by the size of each genomic compartment (e.g., the fraction of the genome covered). The exonic fraction has ~2x as many nullomers than the intronic fraction despite exons covering a much smaller proportion of genome space. I take this as support for the selection argument (since nullomers in exons might be especially toxic), but it would help to push the point home a bit more.
  3. I’m confused as to why there are more nullomers in the exonic and CCDS compartments compared to the whole genome. Does “genome” mean intergenic space, or is it the entire genome? Is this entirely due to splicing, i.e., nullomers that span splice junctions?
  4. The authors don’t provide a clear justification for the nullomer and nullpeptide length cutoffs they chose.
  5. I’m not sure that the nullpeptide and cross-species nullomer  scores are effective measures of negative selection. The 3 score does not seem to take the number of nullomers observed in a given genome into account. For example, if a nullomer was excluded from 28 of 29 genomes, but observed in 1 genome, does the metric take into account whether that nullomer was observed once or 100 times? Neither *M* nor *n* are explicitly defined so it’s hard to tell.

This metric also doesn’t seem to take genome size into account. Yeast, fly, worm have small genomes compared to other species.

I’m also concerned about the nullpeptide 1 score. If I understand it correctly, it’s based on permuting (i.e., shuffling) the nullpeptide sequence. I’m not convinced this is the right approach. Selection against nullpeptide generation in proteins would manifest as selection against variants in individual codons that would result in a nullpeptide. The shuffling procedure does not capture this. An alternative could be to consider the frequency of proto-nullpeptides in the proteome and explore the potential variants at nonsynonymous positions that could generate nullpeptides, and then compare that to actual human variation at those positions. The variant occurrence and allele frequency distributions should be low even compared to what would be expected for nonsynonymous variants in general.

- 1. The description of the simulation results is confusing as well. The authors are talking about enrichments of nullomers in the genome compared to simulated data, even though nullomers are by definition absent from the genome. If I understand the analysis, the simulations are meant to compare rates of nullomer absence in simulated data versus the real genome, so “enrichment” in this case means nullomers in the real genome are more often observed *as sequences* in the simulated genomes. This needs to be clarified or many readers will be hopelessly lost.
  2. I’m confused about how the distribution of potential single variants that can regenerate nullomers suggests selection. If nullomers are toxic (for some unknown functional reason), proto-nullomers may still be somewhat toxic. In that case, selection would favor additional mutations that degrade them further, in which case they would no longer be proto-nullomers. Are nullomers that have corresponding proto-nullomers in the genome actually at the weak end of the selection distribution, and higher-order nullomers are extremely disfavored? Some discussion on this point would be helpful.

Also, at k=13, does the fact that there are so many observed human variants that create nullomers argue against constraint? It would be useful to know the frequency distribution of nullomers, not just the variants (including nullomers that arise due to more than one variant).

The figures are a bit rough, hard to read and need to be cleaned up prior to publication: Figure 2d. At k=11, why is the genome-wide value below zero?

The colors in Figure 2e are hard to distinguish from each other. Also, why do nullomers at k=15 drop in frequency at 29 species? Is this a function of phylogenetic distance (where a nullomer appears in a distant species, such as yeast, fly or worm, but not the others)?

In Figure 3 c and d, the authors need to make it clear that panel c is showing all potential mutations (including indels) and panel d is showing observed variants in human genomes.

In Figure 4a, why does the x axis start at 0?

The genome sizes in Figure 6 seem off – the human genome is not 1e6 kb. Why are the distributions in Figure 6b approximately U-shaped?

**Reviewer 2**

The nullomer analysis has been of interest for many years, because many have been interested in this topic of missing k-Myers. After the initial report of nullomers and ascribing a deadly role for them, it was shown that a deficit of k-mers in non-functional regions can easily arise by CpG hyper mutations. That is, the observed pattern was largely trivial, and driven by mutation in non-functional regions that comprise 95% of the human genome. Later it was shown that a more Detailed CpG analysis would be useful, but nothing changed fundamentally. Anyway, this was nit unexpected because genome is a complex place with many short and long range mutational and interaction always differences.  

In my opinion, this article makes much less impressive contributions than those published in PLoS One in 2016 (cited ref#2) and earlier article (cited ref #3). Current authors add the layer of the effect of purifying selection to this, which will obviously eliminate some nucleotide and peptide k-mers. One wonders why that is surprising. Showing some k-mer enrichment by using null distribution from genome shuffling is not biologically meaningful, as it does not present a mechanism (e.g., CpG hyper mutations) and because random genome shuffling distributions do not always produce null distributions.  Of course some amino acid changes at each position are detrimental, which will create some nullomers at some some value of k. So, what does that tell us beyond what is already known. Overall. this work is a descriptive bioinformatics exercise with no new mechanistic insight and an attempt to tells us the obvious that some peptides are less frequent than expected against a null distribution derived based in many assumptions of site independence, mutational homogeneity, codon usage bias heterogeneity, and equality of selective pressures across species. Removing the impact of so many null possibilities is not possible, certainly not by constructing random samples from the genome.

- Are the methods appropriate to the aims of the study, are they well described, and are necessary controls included? If not, please specify what is required.
Null distributions are not appropriate.

- Are the conclusions adequately supported by the data shown? If not, please explain
The study of tautological, as negative selection will create deficit of peptides. No new mechanism is provided about why any nullomers is absent.

- Are sufficient details provided to allow replication and comparison with related analyses that may have been performed? If not, please specify what is required.
N/a.
- Does the work represent a significant advance over previously published studies?
No, as noted above. Extension to coding regions just complicates the inference.
- Is the paper of broad interest to others in the field, or of outstanding interest to a broad audience of biologists?
No.

**Reviewer 3**

In this work the authors propose a study on nullomers and nullpeptides, short DNA or amino acid sequences that are absent from a genome or a proteome, respectively.
The study focused on human nullomers even if also other (29) eukaryotic organisms and related nullomers and  nullpeptides were analyzed.
Interestingly the authors also computed nullomer peptides (up to 6 aa) absent from all known protein sequences, namely nullomer primes.
Obtained results mainly aim at providing significant insights on the nature of absent sequences (nullomers): are they the consequence of negative selection or are they simply the consequence of the deployment of CG nucleotides, according to the hypermutability model, so a process mainly driven by chance?
Through three different functions, evaluating, to some extent, the probability of a nullomer to be absent, the authors claim that a negative selection acted on them.
A new aspect introduced in the work regards the analysis of nullomers on different genomic regions  (such as coding, promoters intergenic etc. sequences).
The authors also investigated the relationships between nullomers and SNPs and finally they proposed a model to phylogeny based on absent sequences.

The work presented in this manuscript clearly indicates that a huge amount of data were analyzed and several contexts concerning nullomers were investigated. The paper propose several aspects, presenting related  results, that could be of interest in this context nevertheless some steps of the implemented algorithms are not clearly described and some analysis were already performed in previous works, moreover, in my opinion, the manuscript lacks a clear discussion about the  significance of obtained results. Some result data should be clearly linked to the conclusions the authors claim, in particular regarding negative selection nature of nullomers.
For example the performed analysis concerning Phi1 function is for sure interesting but it is not explained how obtained results could contribute to asses a negative selection on nullomers.

Analysis related to phi2, to some extent, and phi3 were already presented in previous works [2] as well as the phylogenetic analysis based on nullomers.


Major issues

   1. According to [1] prime sequences are defined as: "We term the short sequences that do not occur in a particular species "nullomers," and those that have not been found in nature at all "primes."
The authors refer to primes in the same way (line 30): "By analyzing all protein sequences across the tree of life, we further identify 36,081 peptides up to six amino acids in length that do not exist in any known organism, termed primes." but (in line 60) "A more extreme case of evolutionary exclusion are nullomer primes, kmers that are found absent from all examined species [1] .", and (line 316): "We next set out to identify nullomers that do not exist in any of the 30 organisms, termed as nullomer primes" that is to say sequences absent in the 30 analyzed organisms and not in all known genomes/proteomes.

   2. The authors refer to nullomers "assigned to different functional categories" and it seems like those sequences occur in those functional regions instead of not occurring in those regions, in my opinion it should be clearly explained, to avoid misunderstanding, that nullomers related to those regions do not occur in those regions while they could occur when considering the whole genome.

   3. Line 180:
The authors claim: "For 13bp nullomers, there are 13 possible deletions (14.29% of mutations), 39 possible substitutions (42.86% of possible mutations) and 39 possible insertions (42.86% of mutations)." Why do the authors consider 39 possible insertions ? After each of the 13bp each of the four nucleotides can be inserted and not only 3, so 13*4 = 52.

   4. Line 149: "Combined, our scoring metrics (φN) show that nullomers are under selective pressure."
This is a major issue of the work.  phiN is not defined in the methods and there is not a clear formalization expressing how to put together the three measures, providing a link between data obtained in the work and the conclusion that nullomers are under negative selection.
I am pretty confident that  those data could contribute to this aim but it not clearly formalized in the manuscript.

   5. The authors should discuss in more detail the differences found in the analyzed regions. For example considering coding sequences trivially there are stronger constraints because of the codon structure (and codon usage) in those regions.

   6. It is interesting and it would deserve to be discussed in detail the finding reported in line 185-187 "Substitutions were further analyzed and we found that A->C, T->C and G->C are the most frequent substitution types (Fig. S2a-c)." Those substitutions lead to the appearance of nullomers so it is reasonable to hypothesize that reverse mutations occurred in the evolution process to make nullomers absent sequences. All those identified substitutions involve C nucleotide, in particular C > T is the mutation that characterizes the ipermutability model (see Sved J, Bird A. 1990. The expected equilibrium of the cpg dinucleotide in vertebrate genomes under a mutation model. Proc Natl Acad Sci USA. 87(12):4692-4696) leading to depletion of CG nucleotide.

   7. Regarding the 30 eukaryotic organisms considered in this study it is worth noting that at least two of them Caenorhabditis elegans and Saccharomyces cerevisiae (commonly named Yeast) are significantly far in the phylogenetic tree of life from other species and they have a very different genome size (~100 Mbp and ~12 Mbp respectively) when compared to other considered organisms (human genome for example ~3 Gbp). The size of genome deeply impacts on the number of nullomers: the greater the genome size the higher the number of present sequences the lower the number of nullomers.
This could result in a bias in the Jaccard index since if hypothetically Yeast had a number of nullomers 100 times higher than a given species S then the Jaccard distance between the two species would not be able to be smaller than 99/100 (1-1/100), even if all nullomers of S were also nullomers of Yeast. In other words the two species  Caenorhabditis elegans and Saccharomyces cerevisiae would be far from all other species even if they shared nullomers.  
So Jaccard is not a suitable Index in the case you have to compare sets of sequences showing very different sizes.      

   8. In the Caption of Figure 7: Evolutionary relationship of nullomers and nullpeptides across 30 species. Figure 7 panel b is referred to 29 species; the same occurs in Fig S7.

   9. The hierarchical clusters depicted in Fig7 and Fig S7 both A and B panels are not consistent, clusters in the left and right panels are different. For example in Fig 7 Panel A for k = 15bp  Zebrafish falls in the cluster of Yeast, C. elegans and Drosophila in the right hierarchical clustering but in the cluster with Lizard and Chicken in the right panel.
The color associated to Primates, Non-Primates - Mammals and Non Mammals are not consistent (for example in Fig S7 both A and B cat is assigned to Primates in the left panel while it is correctly assigned to Non-Primates Mammals in the right panel).
   
   10. Line 501 regarding formula of Phy1 it is not reported that k is the length of nullomers and it is not explained why Ani is diveded by 3k, that should be the number of possible sequences obtained by single mutations.

   11. In the section "Higher order nullomers as a ranking criterion" (line 152) the authors introduced high order nullomers but high order nullomers were already introduced by Vergni and Santoni 2016, no reference is provided.

   12. The authors should discuss and mention that phylogenetic trees based on nullomers were already introduced in previous works (see [2] - Vergni and Santoni 2016; Garcia SP, Pinho AJ, Rodrigues JM, Bastos CA, Ferreira PJ. Minimal absent words in prokaryotic and eukaryotic genomes. PLoS ONE. 2011; 6(1):16065 doi: 10.1371/journal.pone.0016065), the authors extended the phylogenetic analysis to 30 species. Moreover it was clearly showed in [2] that phylogenetic trees based on high order nullomers are significantly more effective than those built on simple nullomers "The trees T2 and T4, based on first order nullomers of size 14, show an overall higher accuracy with respect to T1 and T3, based on simple nullomers of size11, indicating that higher order nullomers seem to be more conserved among close species."

   13. Concerning phi2 function it is not clear what do the authors mean by "controlling for mononucleotide, dinucleotide and trinucleotide" in the 100 shuffled sequences, obtained through Ushuffle package.
Are the frequencies of mono-di-trinucleotide conserved in different shuffled sequences? How many simulation with the same monucleotide frequencies? How many with dinucleotide and trinucleotide frequencies?
Moreover a similar analysis, to some extent, was performed in [2] where "random sequences of the same length of the human genome either with the same nucleotide frequencies (nu) or with the same dinucleotide frequencies (di)" were generated for mono and di-nucleotides.
The authors should discuss and compare obtained results with those obtained in [2].

   14. In figure 2 panels c all identified nullomers for length 10-15 were put together, since the most  part of nullomers are of size 15 (for example for genic regions 6,141,882 nullomers for 15 bp, 39,268 for 14 bp and only 10 for size 10-13)  , the histogram showed in panel c is practically almost the same of that of panel d related to 15.  


Minor issues

Concerning Figure 3 panel b I would suggest to swap x and y axis to make it more clear.

Concerning Figure 2 panel e I would suggest to change the colors since it is not easy to distinguish different colors that are close to each other.

Figure 1 panel a seems not to add any relevant or further information.

**Authors’ response to reviewers**

We thank the reviewers for their excellent comments which we feel have significantly improved our article. Below is a point by point response to these comments.

**Reviewer #1**

Georgakopoulos-Soares and Barnea et al. analyze the distribution of nullomers and nullpeptides, DNA and protein sequences that are absent from one or more genomes and proteomes, in the human and other reference genomes. They use several metrics to support that nullomers and nullpeptides may be excluded from genomes due to negative selection. They also identify nullomers and nullpeptides in the human genome that could be formed by single mutation events (which for the purposes of this review, I’ll refer to as “proto-nullomers”), and show that nullomers can be reintroduced to extant human genomes via DNA polymorphisms. They then consider the distribution of shared nullomers across genomes, and identify an intriguing set of nullomers, which they term primes, that are absent from all of the genomes they analyzed. Finally, they demonstrate how patterns of nullomer retention or loss can be used for phylogenetic classification.

This is an intriguing, conceptually novel study that brings attention to a phenomenon that has been somewhat neglected in comparative genomics and analyses of genome evolution. Genomes and proteomes are large, but not infinite, so it would not be surprising to identify particular sequences (especially longer sequences) that do not appear in a given genome. However, the authors’ analyses overall support that at least a subset of nullomers are restricted from genomes due to selection rather than random chance. The catalog of nullomers and nullpeptides the authors have assembled will also enable functional studies of these understudied sequences.

Despite my enthusiasm, I do have several comments on the analysis and presentation of the results:

1. I was disappointed that the authors did not consider the exclusion of nullomers in gene regulatory sequences. In their discussion, they say this would be an interesting analysis (mentioning conserved noncoding sequences specifically), but don’t pursue it here. There is now an abundance of highly resolved functional genomic data that could be exploited to identify nullomers that are absent from regulatory elements. For example the ENCODE v3 data includes DNase footprint data from hundreds of human tissue samples and cell lines. These footprints are longer than the nullomers they are considering here, and also include predicted TF binding site data. Identifying the subset of nullomers that are excluded from these sites would be interesting. I’d also like to know how frequent “proto-nullomers” are in regulatory space, whether they correspond to specific TF motifs, and how often they are converted to nullomers by mutation.

We thank the reviewer for their comment and completely agree that analyzing nullomers in gene regulatory elements, not just promoters, would be extremely interesting. Following the suggestion by the reviewer, we analyzed nullomers in ENCODE functional annotations and DNase footprinting datasets. For functional annotations, we have summarized nullomers in the human genome’s non-coding regulatory regions as illustrated in the [ENCODE cCRE](https://genome.ucsc.edu/cgi-bin/hgTrackUi?hgsid=1051867129_mfItAKxBQaFNNXMnbhuU9bPy4ZlA&c=chr17&g=encodeCcreCombined) track over hg38 build (<https://api.wenglab.org/screen_v13/fdownloads/GRCh38-ccREs.bed>) (adding REFs for ENCODE cCRE]. This provides the following regulatory functional categories: Promoter-like, proximal enhancer, distal enhancer, CTCF binding sites and the DNase H3K4me3 signature. Using ENCODE’s annotation, we find that promoter-based nullomers are found first at shorter length (K=12bp) than nullomers in non-adjacent gene regulatory regions like enhancers and CTCF sites, mentioned in the revised text. We expanded on that when we performed the permutation and simulation analysis on the ENCODE annotated genetic sub-compartments in the new version of Figure 1C and 2E and the relevant result sections.


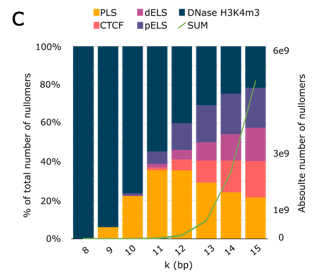


As certain cis-regulatory elements, in particular enhancers are cell-type specific, we expanded our analysis to also include nullomer characterization in the regulatory regions of K562 and HepG2 cell lines, both commonly used cell types in ENCODE and as such have extensive functional genomics datasets available. We performed a similar type of analysis in these cell lines and found the nullomers that are specific to the regulatory regions within each of these cell lines. The results for HepG2 and K562 cell lines are provided below and in Fig S1.


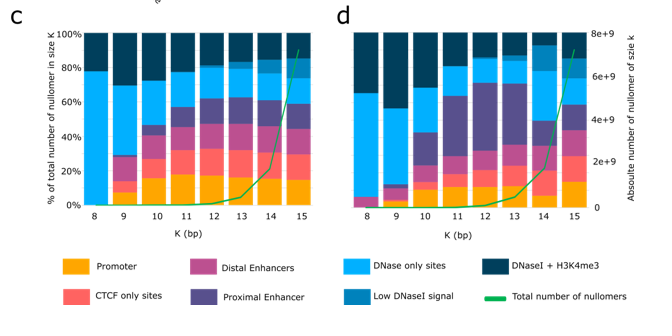


As for DNase footprinting data, this is a great suggestion and we completely agree that it can provide a great opportunity to study proto-nullomer mutations. We have extensively analyzed them in this revised version. Briefly, we used the consensus footprints from the ENCODE v3 consortium. As controls, we used equal sized regions to the left and right of each footprint, from which we calculated the density of proto-nullomer mutations in the footprints and in the flanking regions. We found that DNase footprint data are enriched for proto-nullomer mutations relative to control regions. The results are provided below for k=11 to k=13.


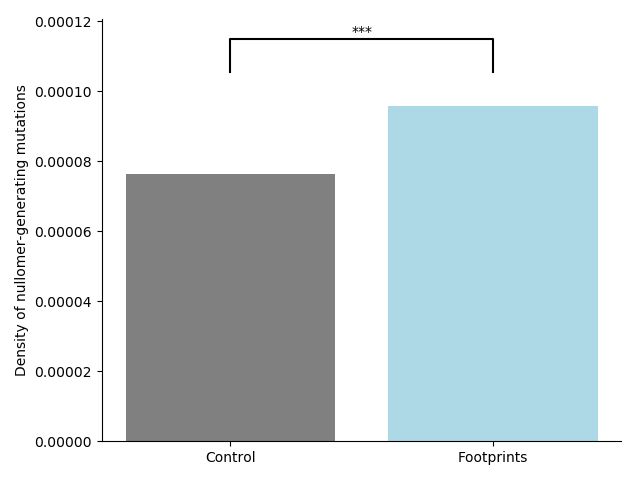

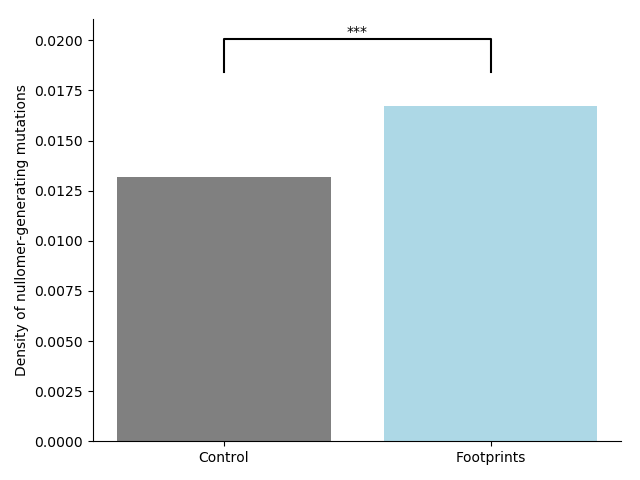

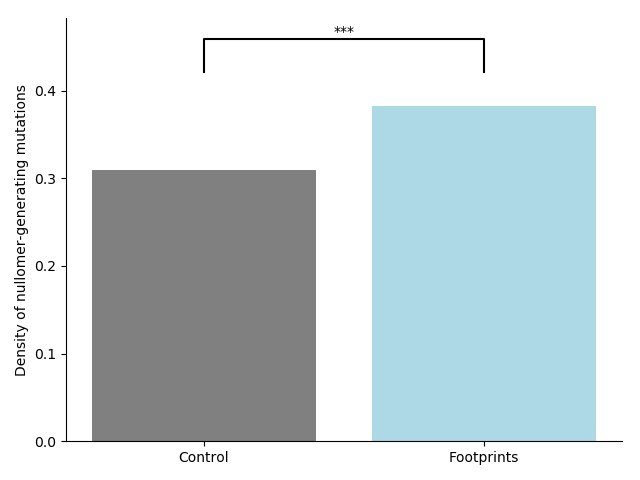


K=11 K=12 K=13

Figure: Density of proto-nullomer mutations in DNase footprinting sites and in adjacent control regions, for nullomer lengths of K=11-13bp.

Binomial tests with Bonferroni correction were performed (p-value<0.0001) in all cases. We also provide below the frequency with which common variants (VAF>=0.05) generate nullomers at DNase footprinting sites for 12mers and 13mers (for 11mers we did not have sufficient data, since the number of total nullomers is low (104) and therefore the number of total mutations is also low).


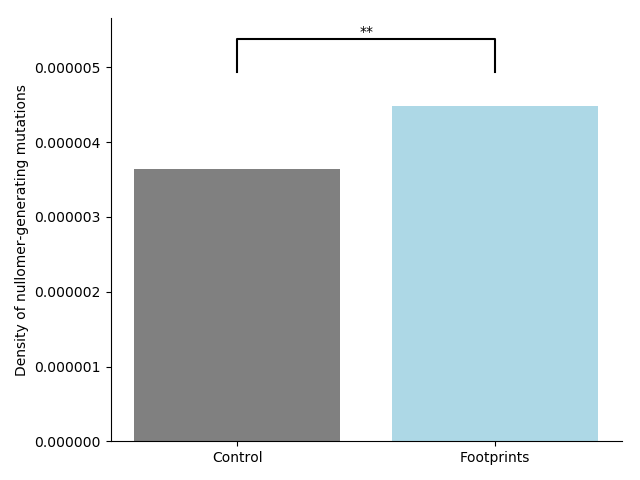

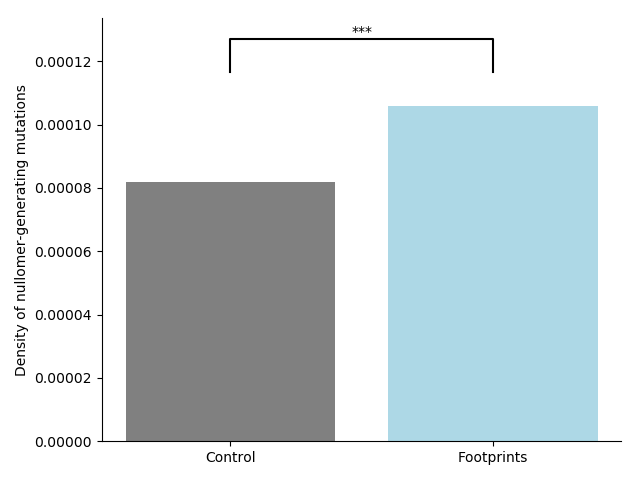


K=12 K=13

Figure: Density of proto-nullomers found as common variants in DNase footprinting sites and in adjacent control regions, for nullomer lengths of K=12-13bp.

Binomial tests with Bonferroni correction were performed (p-value<0.01 for ** and <0.0001 for ***).

2. The potential functional impact of nullomers (in UTRs or promoters, for example) or nullpeptides is not explored in much depth. For example, the authors note that “Additionally, we found that the WCMNW pentapeptide could be generated only by one single nucleotide substitution throughout the human CCDS.” This raises the question of which proteins are sensitive to nullpeptide formation due to variation? Do they converge on specific functions, and/or do such proto-nullpeptides tend to fall in particular protein-coding domains? Are variants that convert proto-nullomers/proto-nullpeptides to nullomers/nullpeptides more likely to lead to repeat reactivation or gene disruption?

We performed a GO-term analysis for three different categories: a) CCDS regions, b) genic regions, c) protein regions. For a-b, we measured the density of putative nullomer mutations across the CCDS regions or across the genic region of each gene, while for c we measured the density of putative nullpeptide mutations across each protein sequence. For the analysis of a-c, we analyzed the top 10% of genes according to their protonullomer mutation density (the number of pronullomer mutations normalized to gene / CCDS / protein length), as well as the bottom 10% of genes according to the same feature (genes with zero protonullomer mutation in them are excluded from this analysis). We found that the top 10% of GENIC and CCDS protonullomer dense genes are most significantly involved in chromatin structure and epigenetic regulation, which was absent from the bottom 10%. This indicates the extent of conservation against the resurfacing of nullomers in genes involved in cell deterministic processes. We have added the following paragraph on nullomer mutations in CCDS regions:

“We wanted to assess whether specific functional categories are associated with genes that have higher and lower putative nullomer resurfacing mutations. We measured the density of putative nullomer resurfacing mutations in CCDS regions for each gene and performed GO term analysis. We found that genes with the highest density of nullomer materializing mutations were associated with epigenetic regulatory processes and DNA molecule organization, whereas genes with the lowest density of nullomer materializing mutations were associated with processes such as cell to cell contact, detection of chemical stimuli, receptors and axonogenesis among other (Fig. 3d, Fig. S4).”

Analyzing the enriched biological process, molecular functions and pathways within protonullomer dense genes vs non dense gene supports the hypothesis of a strong negative selection against the resurfacing of nullomer sequences. Any change to a component of the chromatin structure and the epigenetic regulatory machinery could have far reaching detrimental effects on the cell and exhibit low tolerance for change. Nullomers resurfacing in what we had defined as protonullomer dense genes, either as a single nullomer or in groups, likely define markers for deleterious processes.

Below are the genic nullomer resurfacing 11mer mutations with the GO term analysis across Biological process (BP) / Molecular Functions (MF) / Cellular Compartments (CC) (using enrichGO) and using the REACTOME DB for pathway related gene set enrichment (top 10% left panels and bottom right panels).


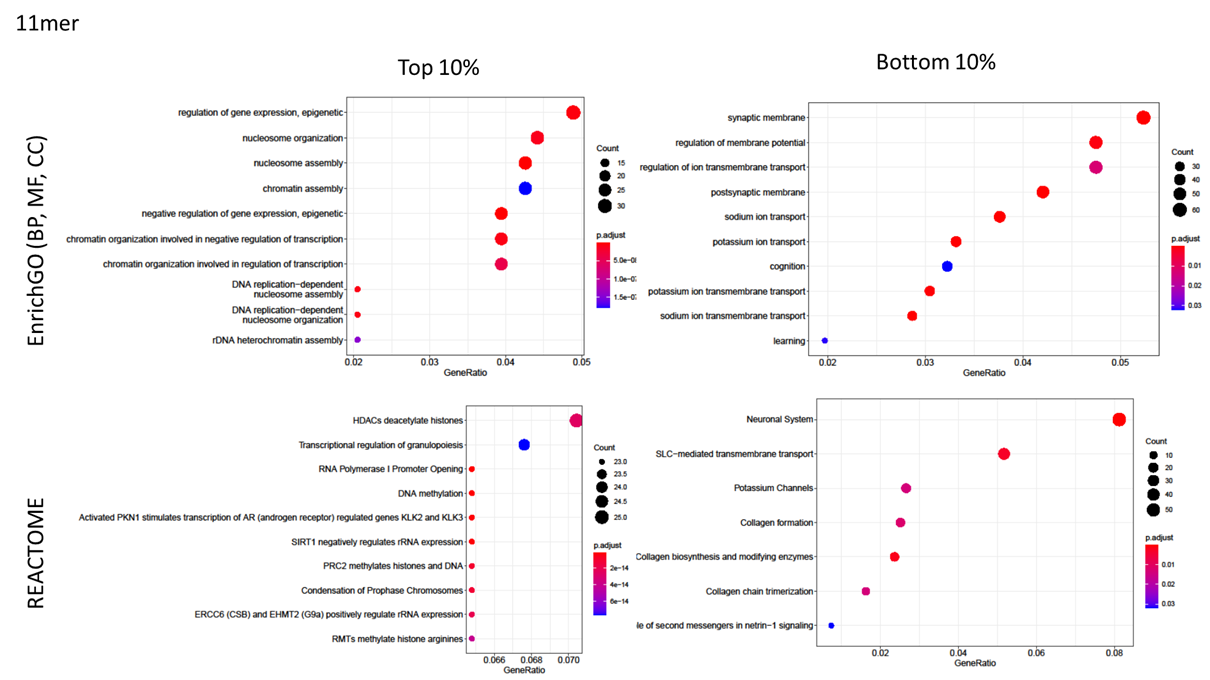


Below are the genic nullomer resurfacing 12mer mutations with the GO term analysis across BP / MF / CC (using enrichGO) and using the REACTOME DB for pathway related gene set enrichment (top 10% left panels and bottom right panels).


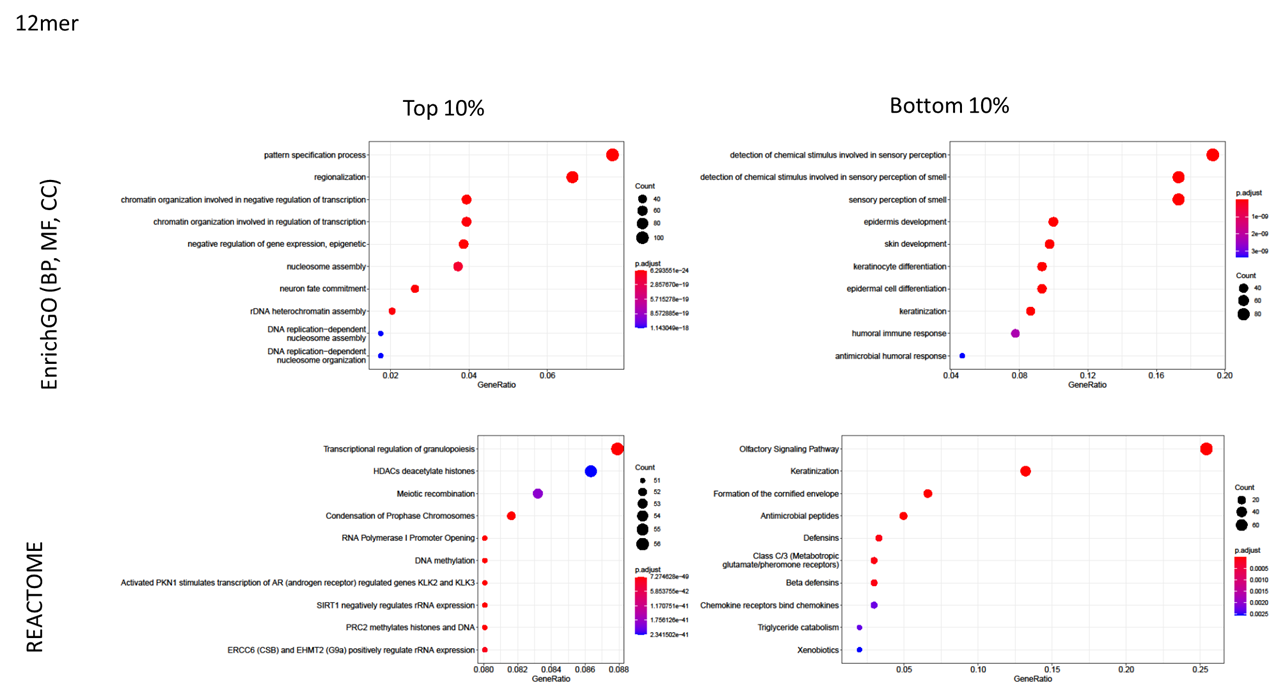


Below are the genic nullomer resurfacing 13mer mutations with the GO term analysis across BP / MF / CC (using enrichGO) and using the REACTOME DB for pathway related gene set enrichment (top 10% left panels and bottom right panels).


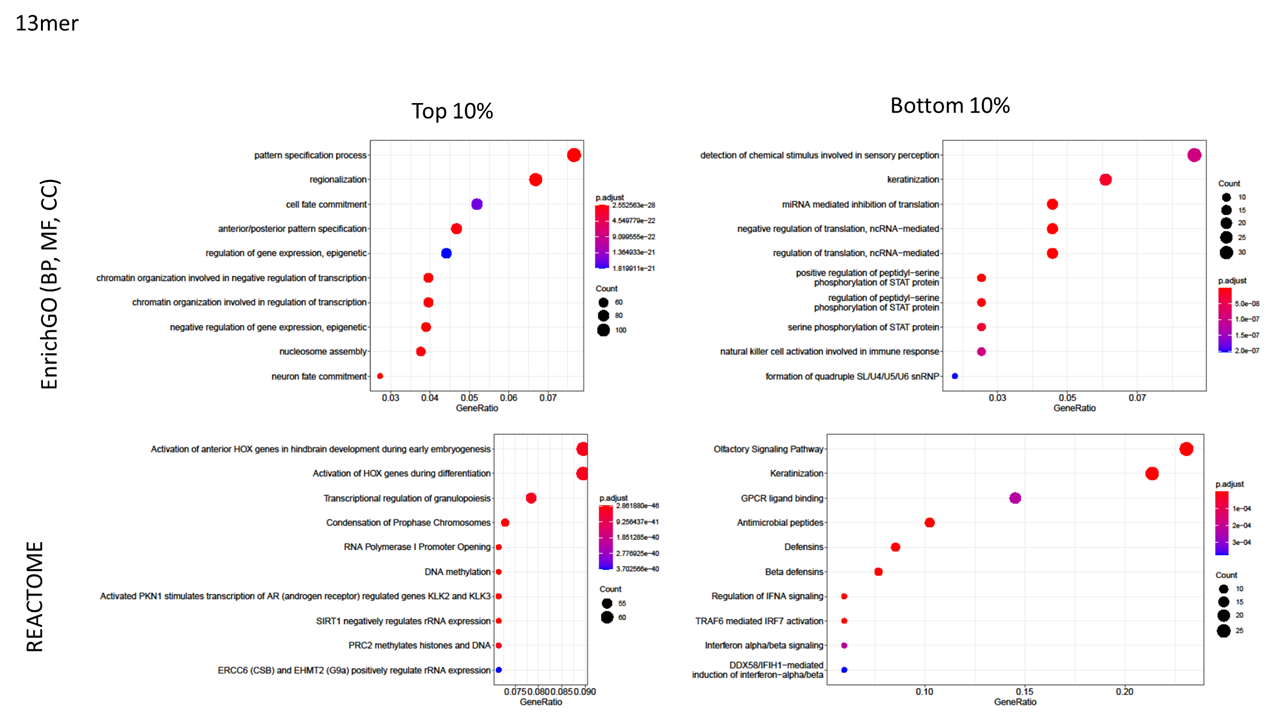


Below are the CCDS nullomer resurfacing 11mer mutations with the GO term analysis across BP / MF / CC (using enrichGO) and using the REACTOME DB for pathway related gene set enrichment (top 10% left panels and bottom right panels).

11mer TOP 10%


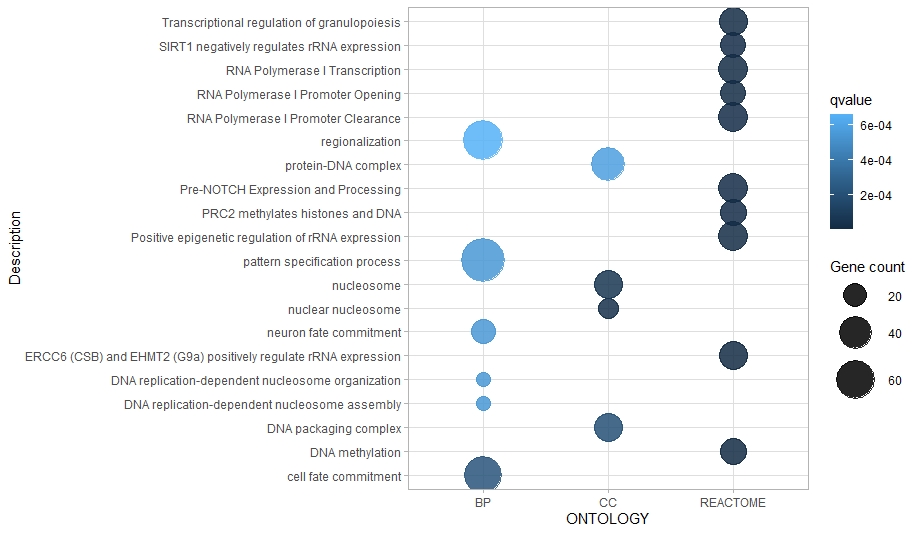


11mer bottom 10%


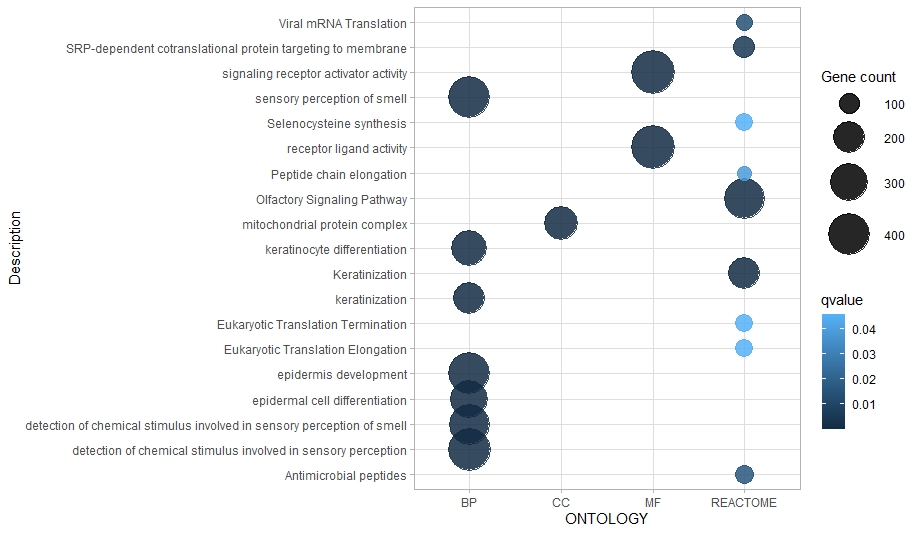


Below are the CCDS nullomer resurfacing 12mer mutations with the GO term analysis across BP / MF / CC (using enrichGO) and using the REACTOME DB for pathway related gene set enrichment (top 10% panels and bottom 10% panels).

12mer top 10%


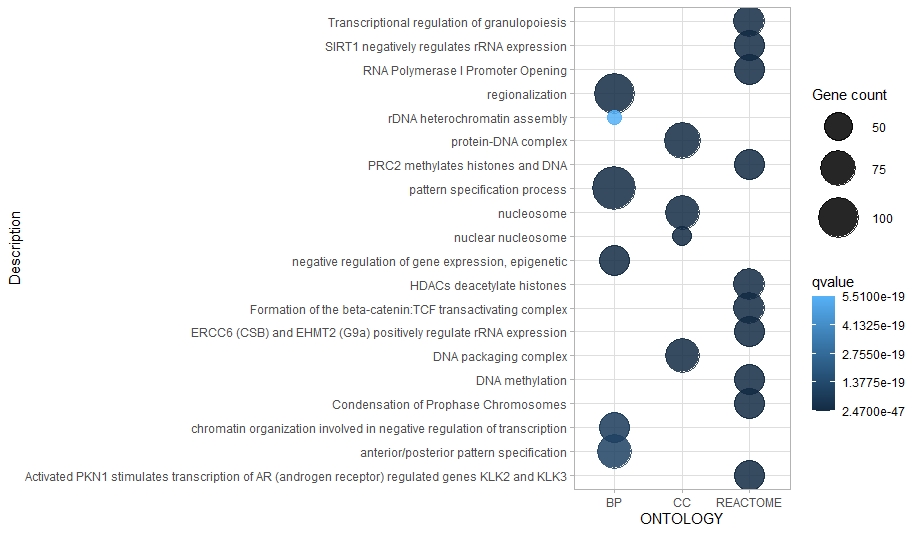


12mer bottom 10%


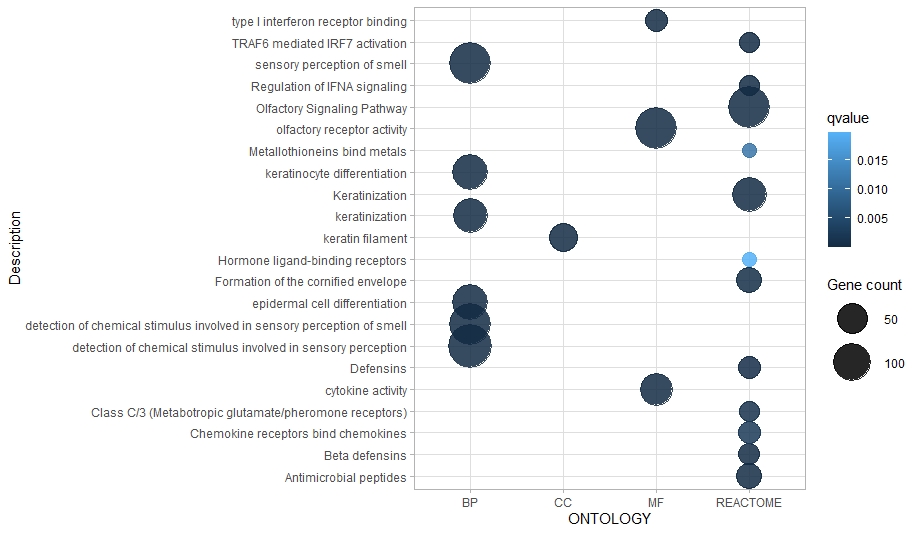


Below are the CCDS nullomer resurfacing 13mer mutations with the GO term analysis across BP / MF / CC (using enrichGO) and using the REACTOME DB for pathway related gene set enrichment (top 10% panels and bottom 10% panels). The results for the top 10% of the genes, based on the nullomer resurfacing mutational density, for nullomer length of K=13bp:


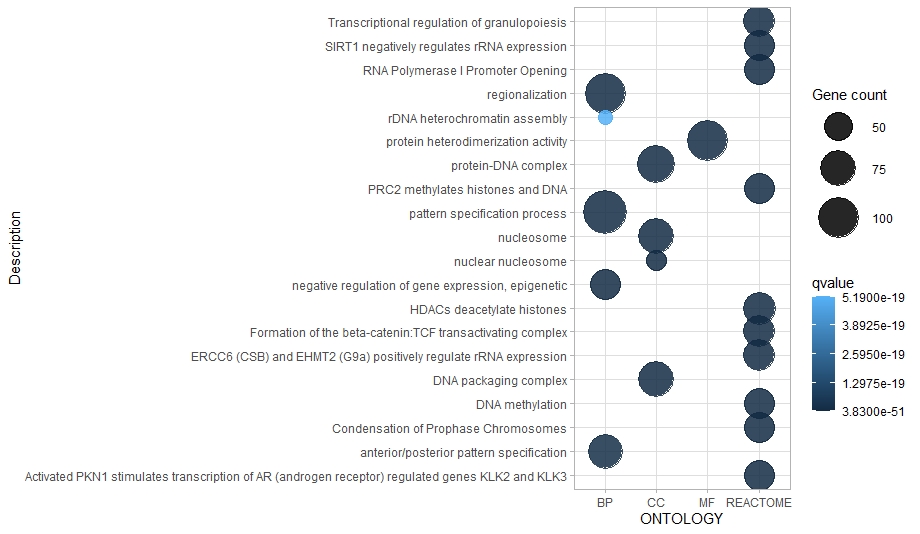


The results for the bottom 10% of the genes, based on the nullomer resurfacing mutational density:
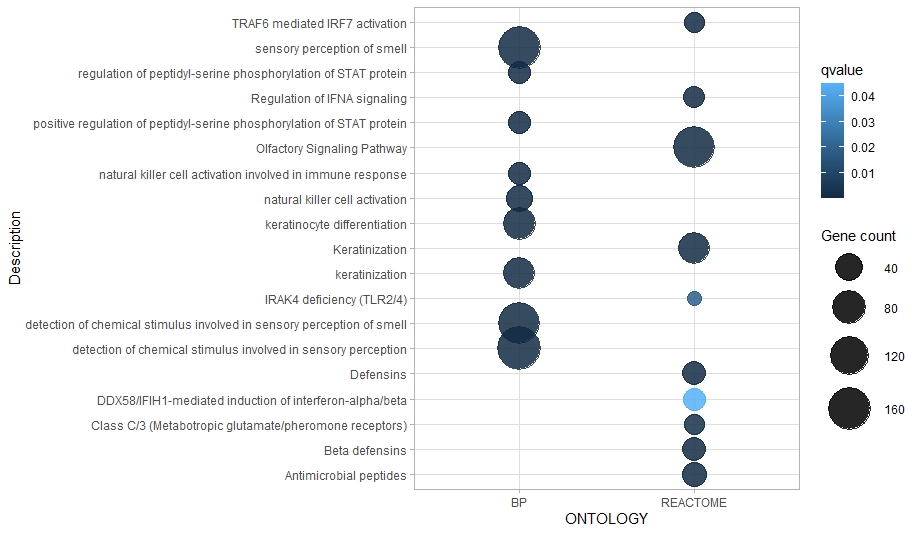


We also conducted the same GO term analysis on top 10% and bottom 10% of genes according to the putative nullpeptide mutational density. More specifically, we calculated the possible amino changes across each protein that can cause the resurfacing of a nullpeptide and calculated the density over the protein length for each gene. Below are the putative nullpeptide resurfacing 11mer mutations with the GO term analysis across BP / MF / CC (using enrichGO) and using the REACTOME DB for pathway related gene set enrichment (top 10% of genes panels and bottom 10% of genes panels).


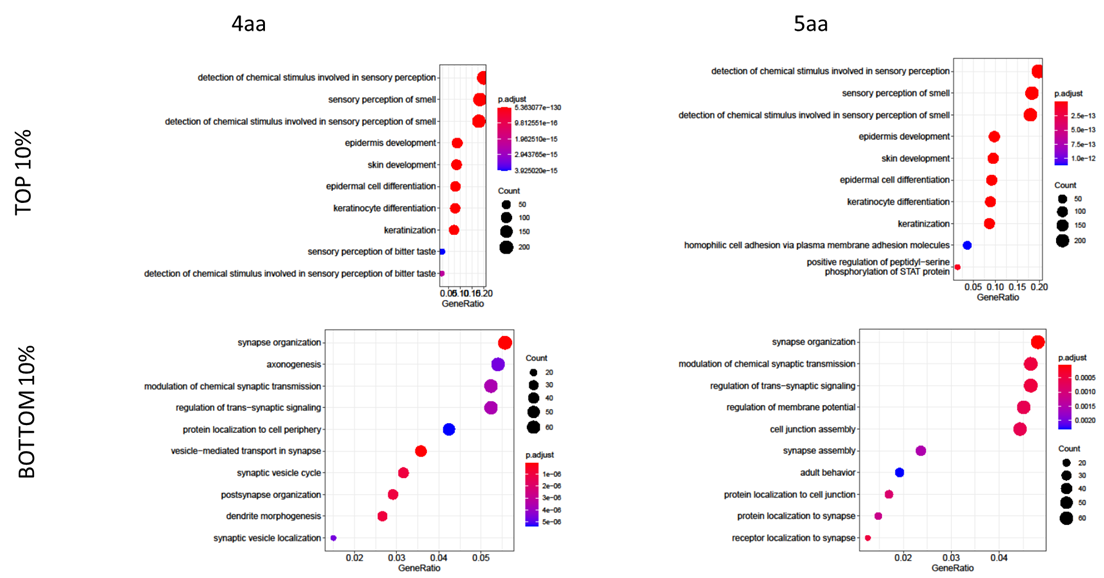


Using the REACTOME pathway enrichment analysis tool.


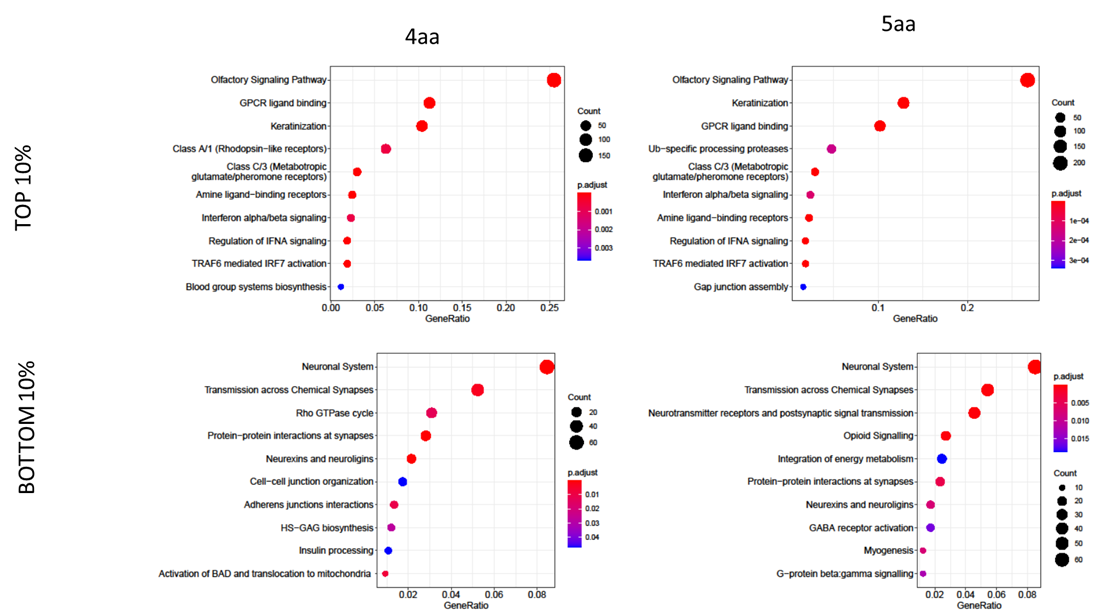


The identification of a small set of nullomer-materializing mutations found in Alu repeats, could suggest an Alu inactivation mechanism, in which case the resurfacing of nullomers at those repeats could re-activate them. The most profound example was that a single nullomer sequence could appear through one base-pair indels or substitutions across more than 120,000 positions in the human genome, but was never observed. We are currently working to experimentally investigate the underlying mechanisms through a variety of complex experiments.

3. The presentation of the results and methods is a bit confusing, and needs to be clarified. This is particularly important for this paper, since the authors are analyzing the absence of sequences from the genome. This requires the reader to – in a way – think “backwards” and as such they could get tripped up. I point out specific examples below.

a. The terminology “nullomer conservation” is a bit confusing. I take it to mean that the same nullomer is absent from the specified genomes, but some readers may not get this.

We thank the reviewer for this comment and completely agree that this can be confusing. To address this throughout, we have changed “nullomer conservation” to explicitly state that a sequence is defined as a nullomer for genome X and genome Z, instead of saying it is conserved between them.

From:

”It was also shown that some nullomers are conserved between closely related species, for example gorilla, chimp, and mouse”

To:

“It was also shown that some nucleotide or amino acid sequences are still missing when examining closely related species, for example gorilla, chimp, and mouse.”

From:

“To identify nullomers that are conserved across evolution and in specific classes, order or species, we repeated our analyses on 29 additional eukaryotic species.”

To:

“To identify nullomers that are persistently missing across evolution and in specific classes, order or species, we repeated our analyses on 29 additional eukaryotic species.”

b. It would help in Table 1 to show the proportion of nullomers by the size of each genomic compartment (e.g., the fraction of the genome covered). The exonic fraction has ~2x as many nullomers than the intronic fraction despite exons covering a much smaller proportion of genome space. I take this as support for the selection argument (since nullomers in exons might be especially toxic), but it would help to push the point home a bit more.

We have changed the table to also reflect the size of the search space and report the size of each genomic compartment.

c. I’m confused as to why there are more nullomers in the exonic and CCDS compartments compared to the whole genome. Does “genome” mean intergenic space, or is it the entire genome? Is this entirely due to splicing, i.e., nullomers that span splice junctions?

Genome in the context of the paper means the whole genome including intergenic and genic regions. There are more nullomers in CCDS and exonic regions due to a smaller search space (<2% of the genome). To clarify that we expect a larger number of nullomers due to smaller sequence space in the genomic subcompartments we added the following sentence:

“We next characterized nullomers in different functional categories. These include genic regions [mRNA sequence from transcription start site (TSS) to transcription end site (TES)], consensus coding sequences (CCDS), exons (both coding and noncoding), introns, 5’UTR, 3’UTR and promoters (defined as -2500 to +500 around the TSS) (see Methods). **Because these regions are subsets of the whole genome, the number of nullomers identified is larger.**”

As also mentioned in the previous comment, we have added to Table 1 the size of the search space for each category.

d. The authors don’t provide a clear justification for the nullomer and nullpeptide length cutoffs they chose.

The lower length we select as the length at which nullomers and nullpeptides start to appear. The upper length limit we selected as the largest kmer length, for which the number of kmers is higher than the number of nullomers. For larger lengths, the majority of possible kmers do not appear in the genome, in which case it is harder to characterize the subset of nullomers that are biologically relevant.

We have now added the following sentence to reflect this:

“The shortest nullomer length studied was the minimal length at which nullomers appeared. The upper nullomer length was selected as the largest kmer length for which the number of kmers found in the genome is higher than the number of nullomers. For larger lengths, the majority of possible kmers do not appear in the genome, making it harder to characterize the subset of nullomers that are biologically relevant.”

e. I’m not sure that the nullpeptide and cross-species nullomer j scores are effective measures of negative selection. The j3 score does not seem to take the number of nullomers observed in a given genome into account. For example, if a nullomer was excluded from 28 of 29 genomes, but observed in 1 genome, does the metric take into account whether that nullomer was observed once or 100 times? Neither M nor n are explicitly defined so it’s hard to tell. This metric also doesn’t seem to take genome size into account. Yeast, fly, worm have small genomes compared to other species. I’m also concerned about the nullpeptide j1 score. If I understand it correctly, it’s based on permuting (i.e., shuffling) the nullpeptide sequence. I’m not convinced this is the right approach. Selection against nullpeptide generation in proteins would manifest as selection against variants in individual codons that would result in a nullpeptide. The shuffling procedure does not capture this. An alternative could be to consider the frequency of proto-nullpeptides in the proteome and explore the potential variants at nonsynonymous positions that could generate nullpeptides, and then compare that to actual human variation at those positions. The variant occurrence and allele frequency distributions should be low even compared to what would be expected for nonsynonymous variants in general.

Following this comment, in our revised version, we provided much more detail and clarification on our methodology. For the evolutionary score φ3 - we inserted definitions of “M” and “n” in the methods section:

“where M is the number of species that include nullomer N and n is the total number of species examined.”

We believe that the number of species in which a nullomer is absent from is more appropriate than the number of total occurrences of a nullomer across the 29 other species, which does not take into account the possibility of a nullomer being highly prevalent in only one very distant species. We had examined the variability of nullomer occurrences between species and frequently found outliers, meaning 1/29 of species presented with a large number of a Nullomer(i) occurrences. Therefore, we concluded that using the number of occurrences in φ3 in this paper would be less informative than the current method with which it is computed.

We agree with the reviewer that common variants could be used to filter out nullomers that are most likely not under selection pressure. We have now added the estimation of negative selection for the resurfacing of nullomers through common variants in the human population. Similarly, we have performed the same process for nullpeptides, finding the set of nullpeptides that can emerge due to common variants and which therefore are less likely to be under selection.

We have now performed the analysis of estimating the proportion of nullomers that resurface in the human population due to population variants and have added the following paragraph:

“We estimated the proportion of nullomers that resurface in the human population through common variants (aggregate probability of resurfacing >0.05). We find that for 12mers 35,972 nullomers do not appear through population variants representing 81% of the nullomers for this length. Similarly, for 13mers, we find that 2,126,810 of the nullomers do not appear, representing 91% of nullomers for that length. We also estimate the likelihood of nullomers resurfacing with a probability threshold of >0.01 and find that 70% of 12mer nullomers do not resurface, while for 13mer nullomers 84% do not resurface.”

The same analysis is performed for nullpeptides for which we have now added the following paragraph:

“We estimated the proportion of nullpeptides that resurface in the human population through common variants (aggregate probability of resurfacing >0.05). We find that for 4aa nullpeptides 307 do not appear through population variants representing 97% of the nullpeptides for this length. Similarly, for 5mers, we find that 603,910 of the nullomers do not appear, representing 98% of nullomers for that length. We also estimate the likelihood of nullpeptides resurfacing with a probability threshold of >0.01 and find that 94% of 4aa nullpeptides do not resurface, while for 5aa nullpeptides 96% do not resurface.”

f. The description of the simulation results is confusing as well. The authors are talking about enrichments of nullomers in the genome compared to simulated data, even though nullomers are by definition absent from the genome. If I understand the analysis, the simulations are meant to compare rates of nullomer absence in simulated data versus the real genome, so “enrichment” in this case means nullomers in the real genome are more often observed as sequences in the simulated genomes. This needs to be clarified or many readers will be hopelessly lost.

We thank the reviewer for pointing this out and apologize for the confusion. We went through the article and found 15 mentions of “enrichment”. We have now either revised our statement or we clearly define the enrichment prior to providing the results. Below we provide the changes in the manuscript:

From: “We next characterize all possible single base pair mutations that can lead to the appearance of a nullomer in the human genome, observing an enrichment for specific nullomer sequences in transposable elements, likely due to their suppression.”

To: “We next characterize all possible single base pair mutations that can lead to the appearance of a nullomer in the human genome, observing a higher number of mutations than expected by chance for specific nullomer sequences in transposable elements, likely due to their suppression.”

From: “While the absence of these sequences could be coincidental, studies of mammalian genomes have revealed a much larger enrichment of nullomers than what would be expected by chance”

To: “While the absence of these sequences could be coincidental, studies of mammalian genomes have shown that a larger number of >10bp genetic sequences are identified as being nullomers than what would be expected by chance.”

From: “All three tiers estimate deviations from the number of the expected occurrences for each kmer motif (not necessarily a nullomer) in order to identify kmers that were enriched or depleted.”

To: “All three tiers estimate deviations from the number of the expected occurrences for each kmer motif (not necessarily a nullomer) in order to identify kmers that occur more or less frequently than expected by chance.”

From: “Simulation scores (φ2) showed a significant nullomer enrichment in the human genome for every nullomer length between K=12-15.”

To: “Simulation scores (φ2) showed a higher number of nullomers in the human genome for every nullomer length between K=12-15, than expected by chance.”

From: “We also performed the same analysis separating the different nullomer lengths, finding that shorter nullomers displayed a higher enrichment in the genomic sub-compartments.”

To: “We also performed the same analysis separating the different nullomer lengths, finding larger differences between the expected and the observed number of nullomers for shorter nullomers in the genomic sub-compartments.”

From: “The expected relative to observed number of nullpeptides showed an enrichment consistent with the results at the DNA level.”

To: “The observed number of nullpeptides was significantly higher than expected based on the simulations, consistent with the results at the DNA level.”

From: “For nullpeptide length of 4aa, the enrichments were 1.96-fold, 1.84-fold and 1.22-fold relative to simulations controlling for mono- di- and tri-peptide content and for 5aa the enrichments were 1.19-fold, 1.14-fold and 1.08-fold relative to mono- di- and tri-peptide controls.”

To: “We defined the enrichment as the number of nullpeptides observed over the average number of nullomers identified across the proteome simulations. For nullpeptide length of 4aa, the enrichments were 1.96-fold, 1.84-fold and 1.22-fold relative to simulations controlling for mono- di- and tri-peptide content and for 5aa the enrichments were 1.19-fold, 1.14-fold and 1.08-fold relative to mono- di- and tri-peptide controls.”

From: “Past work has analyzed the occurrence of 7bp sequences in 11,257 whole human genomes to identify constrained noncoding regions and shows that they are enriched for pathogenic variation [17]. While there are no existing 7bp nullomers, it would be interesting to see whether these constrained sequences are also more enriched for nullomers and if so, how might these missing sequences affect their function.”

To: “Past work has analyzed the occurrence of 7bp sequences in 11,257 whole human genomes to identify constrained noncoding regions and shows that they are enriched for pathogenic variation [17]. While there are no existing 7bp nullomers, it would be interesting to see whether these constrained sequences are also more constrained against nullomers and if so, how might these missing sequences affect their function, when introduced.”

From: “metric score φ2, associated enrichment of nullomers relative to simulations in the genome and various functional categories across K=10-15 bp.”

To: “metric score φ2, number of occurrences of nullomers in the genome and various functional categories relative to their occurrences in simulations across K=10-15 bp.”

From: “metric score φ2, associated enrichment of nullomers relative to simulations in the genome and various functional categories across K=10-15 bp, as a function of nullomer length for K=10-15bp.”

To: “metric score φ2, number of occurrences of nullomers in the genome and various functional categories relative to their occurrences in simulations across K=10-15 bp, as a function of nullomer length for K=10-15bp.”

From: “metric score φ2 associated enrichment of nullpeptides relative to simulations controlled for mono-, di- and tripeptide content of the proteome plotted as a function of nullomer length for K=10-15bp.”

To: “metric score φ2 number of occurrences of nullpeptides in the proteome relative to their occurrences in simulations controlled for mono-, di- and tripeptide content of the proteome plotted as a function of nullomer length for K=10-15bp.”

From: “Fig. S3 Nullpeptides are enriched in the human proteome relative to simulated proteomes.”

To: “Fig. S3 A higher number of nullpeptides is found in the human proteome relative to those found in simulated proteomes.”

From: “We found that CCDS, exonic, 5’UTR and promoter regions were the most enriched for variant-associated nullomers, followed by 3’UTR, genic and intronic.”

To: “We found that CCDS, exonic, 5’UTR and promoter regions had the highest density of variant-associated nullomers, followed by 3’UTR, genic and intronic.”

From: “We found that the two most enriched categories for variants that materialize nullomers were 5’UTR and CCDS regions.”

To: “We found that the two categories with the highest density of variants that materialize nullomers were 5’UTR and CCDS regions.”

g. I’m confused about how the distribution of potential single variants that can regenerate nullomers suggests selection. If nullomers are toxic (for some unknown functional reason), proto-nullomers may still be somewhat toxic. In that case, selection would favor additional mutations that degrade them further, in which case they would no longer be proto-nullomers. Are nullomers that have corresponding proto-nullomers in the genome actually at the weak end of the selection distribution, and higher-order nullomers are extremely disfavored? Some discussion on this point would be helpful. Also, at k=13, does the fact that there are so many observed human variants that create nullomers argue against constraint? It would be useful to know the frequency distribution of nullomers, not just the variants (including nullomers that arise due to more than one variant).

We thank the reviewer for this comment. We agree that variants would be disfavored in nullomeric sequences under negative selection. We find that for a subset of nullomers, there are thousands of possible positions (not variants in the human genome) in which they could resurface, but they are never seen in the genome. These might imply a silencing mechanism. Functional experiments would be required to validate these observations. Since possible variants and actual variants are different (possible mutations, not necessarily being found in the population versus variants in humans) we now clarify and explain these further.

We performed the analysis of estimating the proportion of nullomers that resurface in the human population due to population variants, which we find to be a minority subset. The majority of nullomers either do not resurface or resurface with a probability <0.05 in the human population. We have added the following analysis and text:

“We estimated the proportion of nullomers that resurface in the human population through common variants (aggregate probability of resurfacing >0.05). We find that for 12mers 35,972 nullomers do not appear through population variants representing 81% of the nullomers for this length. Similarly, for 13mers, we find that 2,126,810 of the nullomers do not appear, representing 91% of nullomers for that length. We also estimate the likelihood of nullomers resurfacing with a probability threshold of >0.01 and find that 70% of 12mer nullomers do not resurface, while for 13mer nullomers 84% do not resurface. Therefore, we conclude that the majority of nullomers for lengths K=11 to K=13 do not appear from common variants, which likely reflects selection constraints against those sequences and which is in accordance with our earlier observations.”

We have also added text to the discussion, including an expansion of higher-order nullomers being likely extremely disfavored and how nullomers that appear in the population through common variants are likely not constrained.

“We also observed that the vast majority of nullomers and nullpeptides do not resurface or resurface with a low probability in the human population through common variants. The subset of nullomers and nullpeptides that frequently resurface due to common variants are likely not deleterious.”

h. The figures are a bit rough, hard to read and need to be cleaned up prior to publication: Figure 2d. At k=11, why is the genome-wide value below zero? The colors in Figure 2e are hard to distinguish from each other. Also, why do nullomers at k=15 drop in frequency at 29 species? Is this a function of phylogenetic distance (where a nullomer appears in a distant species, such as yeast, fly or worm, but not the others)? In Figure 3 c and d, the authors need to make it clear that panel c is showing all potential mutations (including indels) and panel d is showing observed variants in human genomes. In Figure 4a, why does the x axis start at 0? The genome sizes in Figure 6 seem off – the human genome is not 1e6 kb. Why are the distributions in Figure 6b approximately U-shaped?

We thank the reviewer for this comment and have tried to address all these comments.

Figure 2d: We used a logarithmic scale and thus the genome-wide value at k=11 being below 0 reflects the ratio of Observed/Expected being below 1. We have now explained the log transformation in the figure legend for clarity.

Figure 2e (now Figure 2f): This figure shows what proportion of nullomers that are identified in humans are also identified as nulllomers in other species. For k=15 we observe a peak at 27-28 species and a slight drop at 29 species. The graph indicates that for k=15 there are slightly more human nullomers that are not nullomers in 1-2 species of our list that nullomers that are absent from all 29 species in our list). We have clarified this in the manuscript, by amending the legend as follows:

“Proportion of nullomers identified in humans that are also identified as nulllomers in other species for nullomer lengths k=11-15 bp. Nullomers that would be placed in the same bin have the same φ3 score.”

We agree that the previous color scheme was difficult to read and have amended the colors to make the different bars more pronounced.

Figure 3c-d: We have corrected this by adding clear headlines in the figures. We also split this between Figure 3c (all potential mutations) and Figure 4a (observed in human genomes)

Figure 3c is provided below:


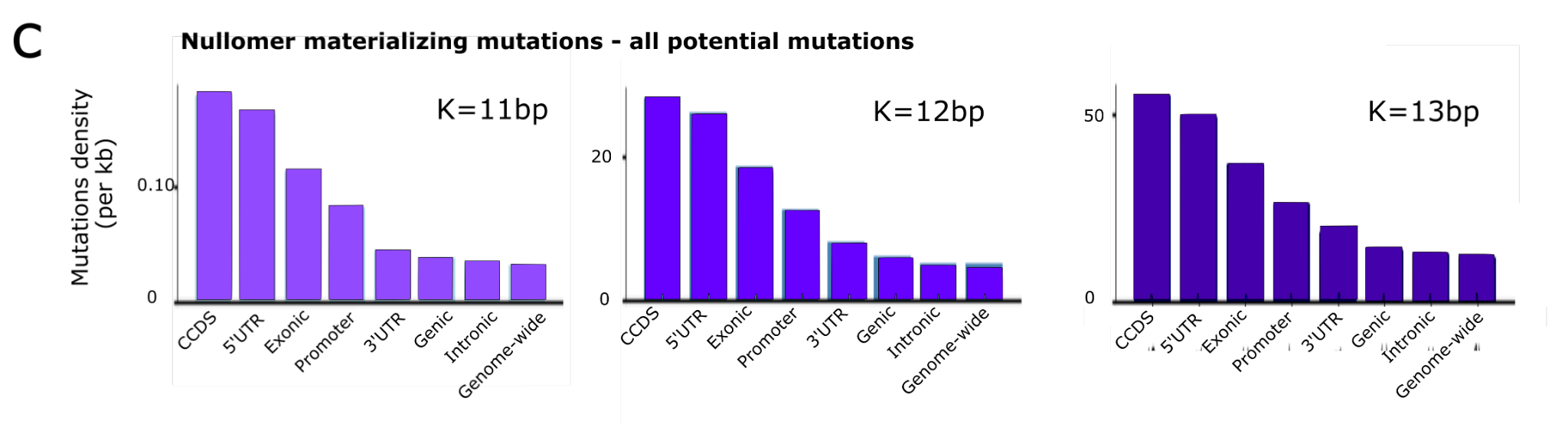


Figure 4a is provided below:


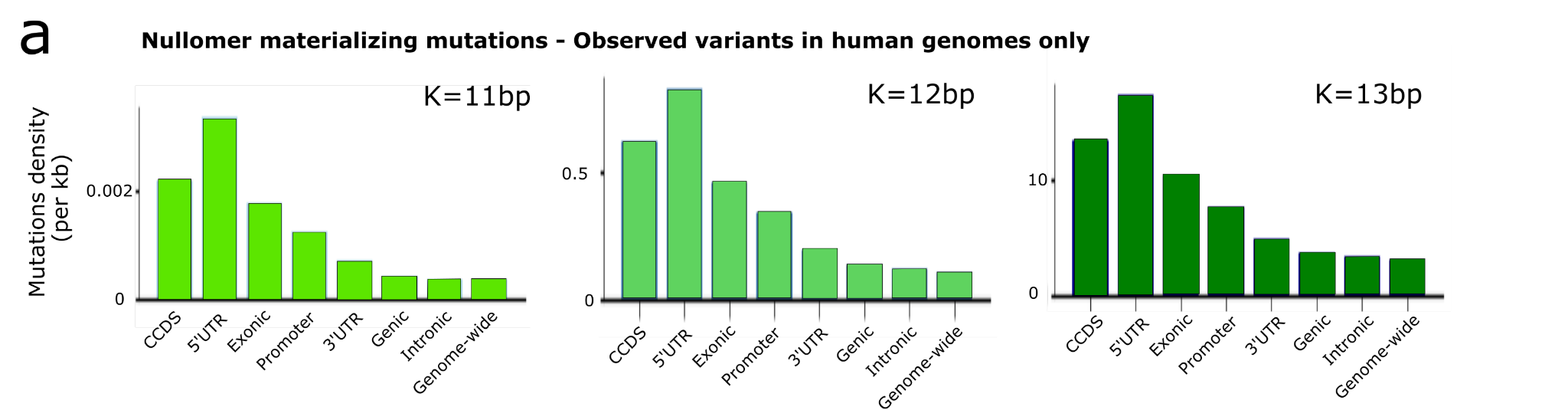


Figure 4b:


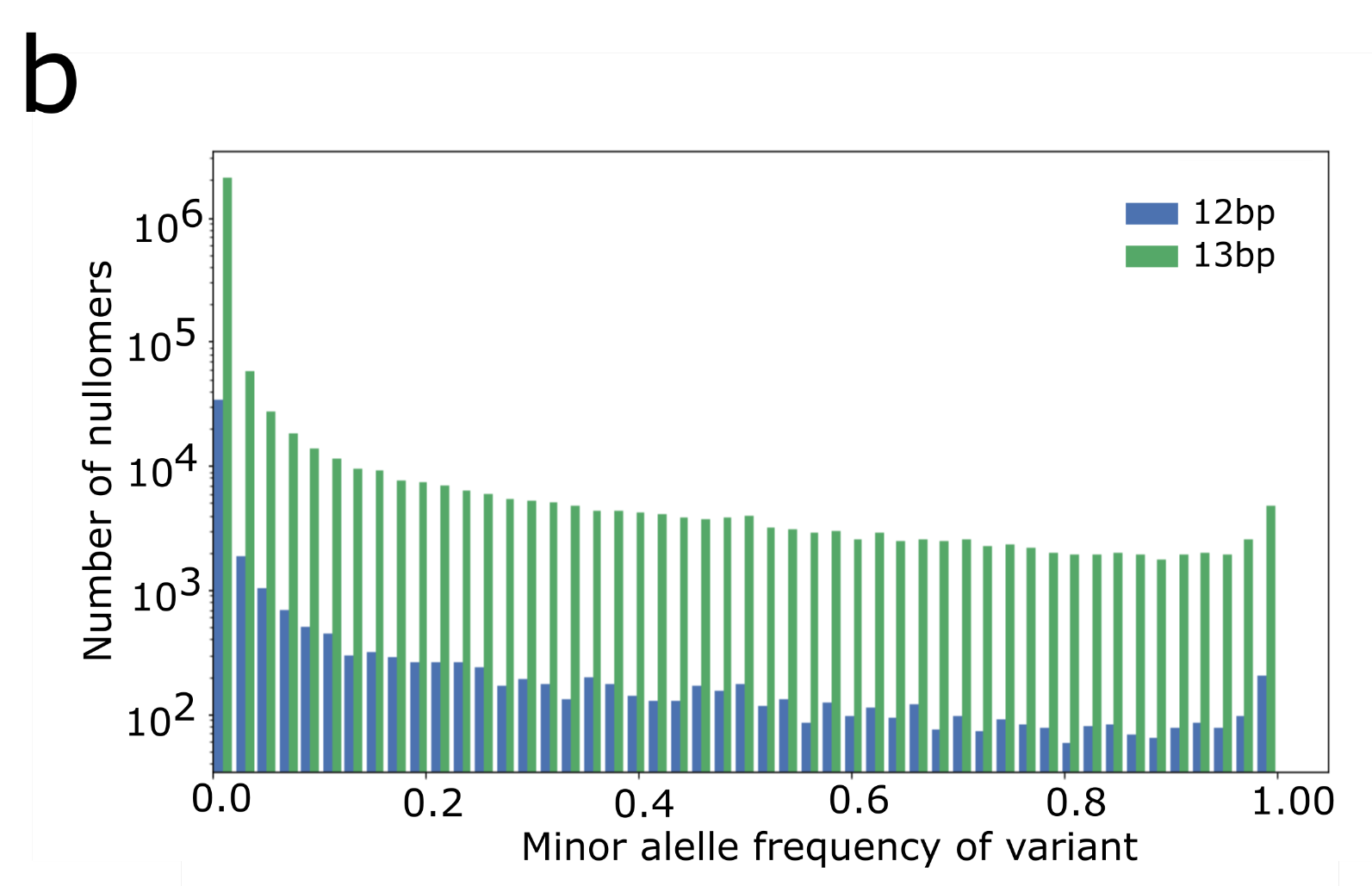


For the first bin, it goes to zero because it also includes extremely rare variants and singletons.

Figure 6: We corrected the issue with the additional y-axis to represent the correct genome sizes.

**Reviewer #3**

In this work the authors propose a study on nullomers and nullpeptides, short DNA or amino acid sequences that are absent from a genome or a proteome, respectively. The study focused on human nullomers even if also other (29) eukaryotic organisms and related nullomers and nullpeptides were analyzed. Interestingly the authors also computed nullomer peptides (up to 6 aa) absent from all known protein sequences, namely nullomer primes. Obtained results mainly aim at providing significant insights on the nature of absent sequences (nullomers): are they the consequence of negative selection or are they simply the consequence of the deployment of CG nucleotides, according to the hypermutability model, so a process mainly driven by chance? Through three different functions, evaluating, to some extent, the probability of a nullomer to be absent, the authors claim that a negative selection acted on them. A new aspect introduced in the work regards the analysis of nullomers on different genomic regions (such as coding, promoters intergenic etc. sequences). The authors also investigated the relationships between nullomers and SNPs and finally they proposed a model to phylogeny based on absent sequences.

The work presented in this manuscript clearly indicates that a huge amount of data were analyzed and several contexts concerning nullomers were investigated. The paper propose several aspects, presenting related results, that could be of interest in this context nevertheless some steps of the implemented algorithms are not clearly described and some analysis were already performed in previous works, moreover, in my opinion, the manuscript lacks a clear discussion about the significance of obtained results. Some result data should be clearly linked to the conclusions the authors claim, in particular regarding negative selection nature of nullomers. For example the performed analysis concerning Phi1 function is for sure interesting but it is not explained how obtained results could contribute to assess a negative selection on nullomers. Analysis related to phi2, to some extent, and phi3 were already presented in previous works [2] as well as the phylogenetic analysis based on nullomers.

We thank the reviewer for the outstanding comments that we have addressed below and significantly improved our revised manuscript. We also want to mention regarding the comment ‘the manuscript lacks a clear discussion about the significance of obtained results’ that we have revised our discussion to address this comment, highlighting what is novel in our study compared to past studies and putting these other studies along with our study in perspective.

**Major issues**

1. According to [1] prime sequences are defined as: "We term the short sequences that do not occur in a particular species "nullomers," and those that have not been found in nature at all "primes."

The authors refer to primes in the same way (line 30): "By analyzing all protein sequences across the tree of life, we further identify 36,081 peptides up to six amino acids in length that do not exist in any known organism, termed primes." but (in line 60) "A more extreme case of evolutionary exclusion are nullomer primes, kmers that are found absent from all examined species [1] .", and (line 316): "We next set out to identify nullomers that do not exist in any of the 30 organisms, termed as nullomer primes" that is to say sequences absent in the 30 analyzed organisms and not in all known genomes/proteomes.

We thank the reviewer for their comment and apologize that our term “primes” was confusing. Primes in the article are the 36,081 peptides are not found in any known organism using the UNIPARC database. However, we completely agree that the term “primes” should not be used when only analyzing 30 species. We have now replaced the term “primes” with the term “nullomers shared across the 30 species”.

We changed this in all instances of primes (nullomeric or nullpeptidic) when referring to the 30 species. More specifically we did the following changes:

1.”Leveraging φ3, the average occurrences in 30 species (Fig. 2e), we identified a total of 124 genome primes of 13bp length, consisting of only 0.00022% of the human genome nullomer space of the same length.”

To: “Leveraging φ3, the average occurrences in 30 species (Fig. 2e), we identified a total of 124 genome nullomers absent across the species of 13bp length, consisting of only 0.00022% of the human genome nullomer space of the same length.”

2.“We next set out to identify nullomers that do not exist in any of the 30 organisms, termed as nullomer primes, and ones that are unique to each species.”

To: “We next set out to identify nullomers that do not exist in any of the 30 organisms and ones that are unique to each species”

3. “For K=12, we did not find any shared nullomers between species. We found 124 nullomer primes at length 13bp (0.00022% of all nullomers of similar length), 272,085 primes at length 14bp (0.1014% of all nullomers of similar length) and 26,010,370 primes at length 15bp (2.4% of all nullomers of similar length).”

To: “We found 124 nullomers absent from all 30 speciies at length 13bp (0.00022% of all nullomers of similar length), 272,085 nullomers absent from all 30 species at length 14bp (0.1014% of all nullomers of similar length) and 26,010,370 nullomers absent from all 30 species at length 15bp (2.4% of all nullomers of similar length).”

4. We removed the sentence: “Nullomers that have a value of φ3=0, are noted as ‘primes’ as they are absent from all species examined.”

2. The authors refer to nullomers "assigned to different functional categories" and it seems like those sequences occur in those functional regions instead of not occurring in those regions, in my opinion it should be clearly explained, to avoid misunderstanding, that nullomers related to those regions do not occur in those regions while they could occur when considering the whole genome.

We now clarify this with the following statement:

Previous: “We assign nullomers to different functional categories (coding sequences, exons, introns, 5’UTR, 3’UTR and promoters) and show that coding sequence and promoter nullomers are most likely to be selected against.”

Changed to: “We defined region specific nullomers as sequences that do not appear in a genomic region, which could either occur as kmers in other genomic regions or be entirely absent from the human genome. We then extracted the set of nullomers in each of the following regions, which represent different functional categories, namely: coding sequences, exons, introns, 5’UTR, 3’UTR and promoters and show that coding sequence and promoter nullomers are most likely to be selected against.”

3. Line 180: The authors claim: "For 13bp nullomers, there are 13 possible deletions (14.29% of mutations), 39 possible substitutions (42.86% of possible mutations) and 39 possible insertions (42.86% of mutations)." Why do the authors consider 39 possible insertions ? After each of the 13bp each of the four nucleotides can be inserted and not only 3, so 13*4 = 52.

We thank the reviewer for identifying this error. Indeed, there are 14 possible deletions, 39 possible substitutions and 48 possible insertions. There are thirteen positions in a 12mer in which any nucleotide can be inserted, to generate a 13mer, therefore 12*4=48 possible insertions. We have now corrected the manuscript to reflect the correct estimates. We provide the revised text below:

“For 13bp nullomers, there are 13 possible deletions (13% of mutations), 39 possible substitutions (39% of possible mutations) and 48 possible insertions (48% of mutations).”

4. Line 149: "Combined, our scoring metrics (φN) show that nullomers are under selective pressure." This is a major issue of the work. phiN is not defined in the methods and there is not a clear formalization expressing how to put together the three measures, providing a link between data obtained in the work and the conclusion that nullomers are under negative selection. I am pretty confident that those data could contribute to this aim but it not clearly formalized in the manuscript.

We thank the reviewer for the comment. Indeed, we did not provide an overall metric based on them. We now provide the following metric in the revised text:

“In order to finalize our overall scoring metric for nullomers, we had employed this population variability analysis, and excluded nullomers that can emerge from human population variance. We have combined all three scores mentioned thus far, and after excluding the common variant resulting nullomers, each nullomer(i) was assigned a summarized score, which will be the basis for future experiments.”

5. The authors should discuss in more detail the differences found in the analyzed regions. For example considering coding sequences trivially there are stronger constraints because of the codon structure (and codon usage) in those regions.

We thank the reviewer for their comment.

We have added in the results the following:

We have changed Table 1 and now provide the proportion of genome each genomic element represents.

In the discussion we have added:

“The latter focused on coding sequences, exons, introns, 5’UTR, 3’UTR, promoters and other non-coding functional elements, and was unique, as only looked at nullomers within these functional units.”

“We observed that coding (CCDS) regions present a higher density of potential nullomer resurfacing mutations (Fig 3c), when compared to the other non-coding regions. This could be a result of the different mode of sequence usage, coding sequence is read in trinucleotide steps that are continuous across the entire region, while the non-coding regions are much more variable at the sequence grammar level.”

This is indicative of a higher constraint on nullomer reappearing in those regions.

6. It is interesting and it would deserve to be discussed in detail the finding reported in line 185-187 "Substitutions were further analyzed and we found that A->C, T->C and G->C are the most frequent substitution types (Fig. S2a-c)." Those substitutions lead to the appearance of nullomers so it is reasonable to hypothesize that reverse mutations occurred in the evolution process to make nullomers absent sequences. All those identified substitutions involve C nucleotide, in particular C > T is the mutation that characterizes the permutability model (see Sved J, Bird A. 1990. The expected equilibrium of the cpg dinucleotide in vertebrate genomes under a mutation model. Proc Natl Acad Sci USA. 87(12):4692-4696) leading to depletion of CG nucleotide.

We thank the reviewer for pointing this out and have added the following text to address this:

“These substitution types are in accordance with the mutation model presented in (Sven and Bird, 1990) and could suggest that reverse mutations occurred during evolution, in turn resulting in nullomer formation. This could partially explain the mechanism driving nullomer sequence content.”

7. Regarding the 30 eukaryotic organisms considered in this study it is worth noting that at least two of them Caenorhabditis elegans and Saccharomyces cerevisiae (commonly named Yeast) are significantly far in the phylogenetic tree of life from other species and they have a very different genome size (~100 Mbp and ~12 Mbp respectively) when compared to other considered organisms (human genome for example ~3 Gbp). The size of genome deeply impacts on the number of nullomers: the greater the genome size the higher the number of present sequences the lower the number of nullomers. This could result in a bias in the Jaccard index since if hypothetically Yeast had a number of nullomers 100 times higher than a given species S then the Jaccard distance between the two species would not be able to be smaller than 99/100 (1-1/100), even if all nullomers of S were also nullomers of Yeast. In other words the two species Caenorhabditis elegans and Saccharomyces cerevisiae would be far from all other species even if they shared nullomers. So Jaccard is not a suitable Index in the case you have to compare sets of sequences showing very different sizes.

We thank the reviewer for the comment. Indeed, the genome size difference results in large differences in the number of the nullomers found in each case. However, this is consistent with the notion that the genomes are dissimilar and is reflected in the Jaccard index. We have now included a sentence clarifying this:

“It is important to note that by definition genomes with widely different sizes will inevitably have a lower Jaccard similarity score. Although these differences make it difficult to interpret the results, we consider this consistent with their biological differences.”

8. In the Caption of Figure 7: Evolutionary relationship of nullomers and nullpeptides across 30 species. Figure 7 panel b is referred to 29 species; the same occurs in Fig S7.

We corrected this to: “Evolutionary relationship of nullomers across 30 species and of nullpeptides across 29 species.”

9. The hierarchical clusters depicted in Fig7 and Fig S7 both A and B panels are not consistent, clusters in the left and right panels are different. For example in Fig 7 Panel A for k = 15bp Zebrafish falls in the cluster of Yeast, C. elegans and Drosophila in the right hierarchical clustering but in the cluster with Lizard and Chicken in the right panel.

The color associated to Primates, Non-Primates - Mammals and Non Mammals are not consistent (for example in Fig S7 both A and B cat is assigned to Primates in the left panel while it is correctly assigned to Non-Primates Mammals in the right panel).

We thank the reviewer for pointing this out. We have now corrected both Fig S7 and Fig 7 accordingly.

10. Line 501 regarding formula of Phy1 it is not reported that k is the length of nullomers and it is not explained why Ani is divided by 3k, that should be the number of possible sequences obtained by single mutations.

We thank the reviewer for identifying this inaccuracy. We have now clarified the formula by rewriting it in the Methods section as:


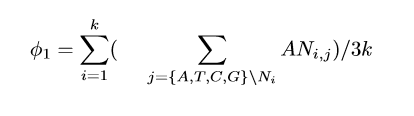


where k is the length of the nullomer N, $N_{i}$ is the i-th bp of nullomer $N$ *and* $AN_{i,j}$ is the number of appearances of the kmer that results by substituting the i-th bp of $N$ by $j$.

11. In the section "Higher order nullomers as a ranking criterion" (line 152) the authors introduced high order nullomers but high order nullomers were already introduced by Vergni and Santoni 2016, no reference is provided.

We thank the reviewer for identifying this omission. We have now clarified this and added the appropriate reference which is provided below.

“An alternate approach to prioritize nullomers that are more likely to have a functional consequence is the characterization of higher order nullomers, as shown previously (Vergni and Santoni, 2016).”

12. The authors should discuss and mention that phylogenetic trees based on nullomers were already introduced in previous works (see [2] - Vergni and Santoni 2016; Garcia SP, Pinho AJ, Rodrigues JM, Bastos CA, Ferreira PJ. Minimal absent words in prokaryotic and eukaryotic genomes. PLoS ONE. 2011; 6(1):16065 doi: 10.1371/journal.pone.0016065), the authors extended the phylogenetic analysis to 30 species. Moreover it was clearly showed in [2] that phylogenetic trees based on high order nullomers are significantly more effective than those built on simple nullomers "The trees T2 and T4, based on first order nullomers of size 14, show an overall higher accuracy with respect to T1 and T3, based on simple nullomers of size11, indicating that higher order nullomers seem to be more conserved among close species."

We thank the reviewer for this comment and apologize for omitting this. We have now added the following sentence to reflect the aforementioned work:

“In accordance with previous work that has shown clustering of species based on nullomers and first order nullomers (Vergi and Santonni 2016; Garcia et al., 2011), we obtained an overall expected tree structure which accordingly clustered together primates, mammals and all the other organisms (Fig. 7a, Fig. S7).”

13. Concerning phi2 function it is not clear what do the authors mean by "controlling for mononucleotide, dinucleotide and trinucleotide" in the 100 shuffled sequences, obtained through Ushuffle package. Are the frequencies of mono-di-trinucleotide conserved in different shuffled sequences? How many simulation with the same monucleotide frequencies? How many with dinucleotide and trinucleotide frequencies? Moreover a similar analysis, to some extent, was performed in [2] where "random sequences of the same length of the human genome either with the same nucleotide frequencies (nu) or with the same dinucleotide frequencies (di)" were generated for mono and di-nucleotides. The authors should discuss and compare obtained results with those obtained in [2].

We apologize for the lack of clarity in how we performed the simulations. Yes, the frequencies of each nucleotide are conserved in mononucleotide shuffles (N=100), dinucleotide frequencies are conserved in dinucleotide shuffles (N=100) and trinucleotide frequencies are conserved in trinucleotide shuffles (N=100). Therefore, for the genome nullomer analysis we simulated the human genome 100 times controlling for mononucleotide composition, 100 times controlling for dinucleotide composition and 100 times controlling for trinucleotide composition, in each chromosome. For each simulated genome we extracted all the nullomers from which we derived the expected number of nullomers in each simulation. The same process was performed in genomic subcompartments. We have now added the following sentences to explain this further:

“As a second metric, we performed simulations controlling for mono (n=100) / di (n=100) / trinucleotide (n=100) content of each regulatory component to estimate the number of occurrences of nullomers by chance in the human genome and its sub-compartments. Ιn each simulation, each sequence was shuffled controlling for mono / di / trinucleotide content.”

“Our second score metric (φ2) is based on a 100-fold Monte Carlo simulation permuting each chromosome of the human genome, or each sequence in the simulated subcompartment, controlling for mononucleotide, dinucleotide or trinucleotide content for each simulation (n=100 simulations were performed in each case).”

Differences with the results in [2] are due to the underlying model that was implemented. In the previous model a Poisson model was used, in which the assumption might not be accurate, and in which they found less nullomers than expected in their model. In our model on simulations, which controls for the dinucleotide content of every sequence or chromosome we find more nullomers than expected. We have now added the following statement to reflect that:

“Simulation scores (φ2) showed a higher number of nullomers in the human genome for every nullomer length between K=12-15, than expected by chance (Fig. 2d), which contradict previous results obtained with a Poisson model (Vergi and Santonni 2016).”

In addition, we provide the code that performs the simulations so that other researchers can reproduce our analysis.

How many simulations with the same monucleotide frequencies? How many with dinucleotide and trinucleotide frequencies?

Simulation of the human genome (and of each genomic sub-compartment) for mononucleotide frequencies was performed a hundred times. In each simulation a new genome was generated and for each chromosome the mononucleotide frequencies were the same as those of the original genome (the same process for dinucleotide and trinucleotide frequencies was followed). Similarly, for genomic subcompartments e.g. promoters, each sequence was simulated, generating a new sequence with the same mono / di / trinucleotide content and then nullomer extraction was performed across each simulated set of sequences and the process was repeated 100 times.

We have now revised the following sentences to make this clear:
“As a second metric, we performed simulations controlling for mono (n=100) / di (n=100) / trinucleotide (n=100) content of each regulatory component to estimate the number of occurrences of nullomers by chance in the human genome and its sub-compartments.”

Changed to: “Our second score metric (φ2) is based on a 100-fold Monte Carlo simulation permuting each chromosome of the human genome, or each sequence in the simulated subcompartment, controlling for mononucleotide, dinucleotide or trinucleotide content for each simulation (n=100 simulations were performed in each case).”

14. In figure 2 panels c all identified nullomers for length 10-15 were put together, since the most part of nullomers are of size 15 (for example for genic regions 6,141,882 nullomers for 15 bp, 39,268 for 14 bp and only 10 for size 10-13), the histogram showed in panel c is practically almost the same of that of panel d related to 15.

We have now put Figure 2c in supplementary material (supplementary figure 1) and presented previous Figure 2d, which provides the same information split by length.

**Minor issues**

Concerning Figure 3 panel b I would suggest to swap x and y axis to make it more clear.

We now provide here and in Figure 3b the updated panels.


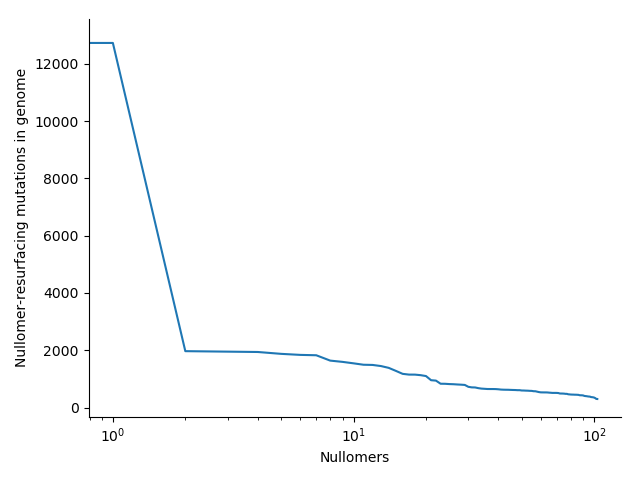

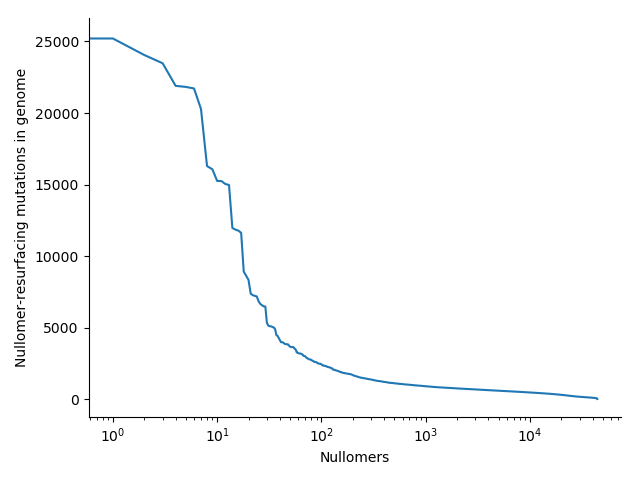

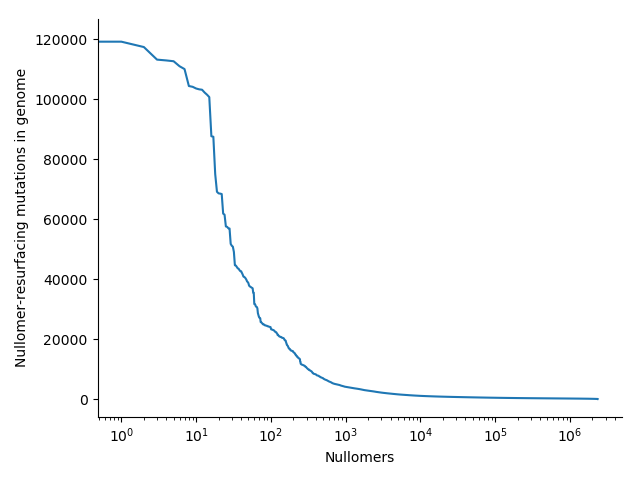


Concerning Figure 2 panel e I would suggest to change the colors since it is not easy to distinguish different colors that are close to each other.

We have now changed the colors and widened the figure panel accordingly. We provide the panel below also.


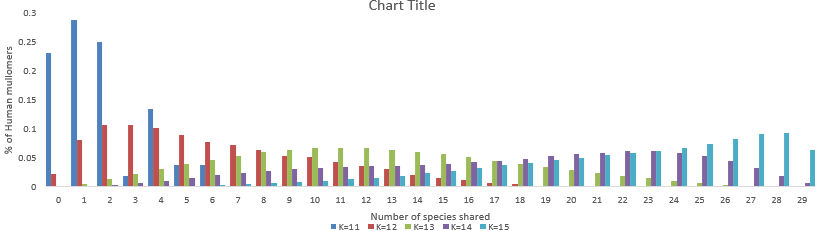


Figure 1 panel a seems not to add any relevant or further information.

We removed this panel.

**Second round of review**

**Reviewer 1**

The reviewers have addressed my major concerns. I have no remaining critiques that should limit the paper moving forward.

**Reviewer 3**

The authors addressed most of the issues raised in first round of review process, adding significant analysis to the work.

Concerning the use of Jaccard index to evaluate the distance of genome with respect to the nullomer sequences I partially agree with the authors.
It is reasonable that two genomes, with very different genome sizes, are distant from each other in the tree but using Jaccard index they are distant from each other independently from their nullomer sequences, so does it make sense to include them in a tree based on differences among nullomer sequences?

Regarding the combined scoring metrics (φN) the authors claim in the revised version of the manuscript: “Combined, our scoring metrics (φN) show that nullomers are under selective pressure” and again “Lacking a conventional statistical measure to determine the significance of a nullomer, we created our own scoring matrix, φ N. The φ N score encompasses three tiers of ranking (Fig. 2a), described in detail in the Methods section.”

phiN is not defined in the methods section or elsewhere, what do the authors refer to as (φN) ? There is not a clear discussion and formalization expressing how to put together the three measures, providing a clear link between data obtained in the work and negative selection of nullomers.

**Authors’ response to reviewers**

Reviewer #3: The authors addressed most of the issues raised in first round of review process, adding significant analysis to the work.

We thank the reviewer for the overall positive assessment.

Concerning the use of Jaccard index to evaluate the distance of genome with respect to the nullomer sequences I partially agree with the authors. It is reasonable that two genomes, with very different genome sizes, are distant from each other in the tree but using Jaccard index they are distant from each other independently from their nullomer sequences, so does it make sense to include them in a tree based on differences among nullomer sequences?

We thank the reviewer for their comment. We have now updated this figure panel and the associated supplementary figure panel to remove distantly related species, namely yeast, C. elegans and Drosophila.

Regarding the combined scoring metrics (φN) the authors claim in the revised version of the manuscript: "Combined, our scoring metrics (φN) show that nullomers are under selective pressure" and again "Lacking a conventional statistical measure to determine the significance of a nullomer, we created our own scoring matrix, φ N. The φ N score encompasses three tiers of ranking (Fig. 2a), described in detail in the Methods section." phiN is not defined in the methods section or elsewhere, what do the authors refer to as (φN) ? There is not a clear discussion and formalization expressing how to put together the three measures, providing a clear link between data obtained in the work and negative selection of nullomers.

We thank the reviewer for their comment. We have now provided a formal definition for φN and added the following statement in the materials and methods to be more clear: “Furthermore, for each of the metrics above, we created a sorted list of nullomers according to their score in ascending order, as we postulate that a lower score in one of the three metrics indicates a higher likelihood of negative selection. In order to identify the nullomers that are most likely candidates for negative selection, we defined the aggregate metric N as the average rank of Nullomeri in the three sorted lists. We hypothesize that the nullomers with the lowest N score are the ones under strong negative evolutionary pressure.

〖φN〗_(〖Nullomer〗_i ) =RANK(〖φ1〗_(〖Nullomer〗_i ) )+ RANK(〖φ2〗_(〖 Nullomer〗_i ) )+ RANK(〖φ3〗_(〖Nullomer〗_i ))

In case several nullomers share the same value at a specific score their ranking will be the same as well per that specific scoring metric. “
